# Supplementary material for: Aminoacyl tRNA synthetases as malarial drug targets: a comparative bioinformatics study
Source: Malar J. 2019 Feb 6;18:34. doi: 10.1186/s12936-019-2665-6 (PMC6366043; doi:10.1186/s12936-019-2665-6)
Supplement: Supplementary file 1 — Additional file 1. A table showing the data set used in the study with Blast details and crystal structures retrieved from the Protein Data Bank. The species, E-value, identity, accession number, PDB ID and sequence lengths are given. [file 12936_2019_2665_MOESM1_ESM.pdf]

Additional file 1 - Table 1: Class I aminoacyl synthetase sequence details.

Species/Protein	E-value	Identity	Accession code	PDB ID	Coverage
**Methionine aminoacyl synthetase**					
Plasmodium falciparum	0	100.0	Q8IJ60		889
Plasmodium malariae	0	78.1	A0A1C3KE62		880
Plasmodium ovale	0	76.9	A0A1A8YPD8		914
Plasmodium knowlesi	0	76.5	B3L2B7		885
Plasmodium vivax	0	75.6	A0A0J9WEH2		886
Plasmodium fragile	0	75.5	A0A0D9QHP9		885
Plasmodium yoelii	0	70.3	A0A078K512		898
Plasmodium berghei	0	69.1	A0A0Y9UYU4		898
Homo sapiens	4.6e-13	24.3	P56192	4BL7,4BVX	900
Toxoplasma gondii	0	42.0	S7VYI1		976
Cryptosporidium andersoni	4.00E-164	48.0	A0A1J4MM36		556
Escherichia coli	4.5E-32	23.2	P00959	3H9C, 1F4L,3H9C	677
Saccharomyces cerevisiae	7.5E-35	24.3	P00958	2HSN	751
**Leucine aminoacyl synthetase**					
Plasmodium falciparum	0	100.0	Q8IBB3		1481
Plasmodium malariae	0	46.6	A0A1A8VSK8		1456
Plasmodium ovale	0	44.9	A0A1A8VTV7		1382
Plasmodium yoelii	0	44.9	V7PGP1		1321
Plasmodium knowlesi	0	44.7	B3L108		1291
Plasmodium berghei	0	45.6	A0A077XBW6		1203
Plasmodium vivax	7.4e-113	53.2	A0A0J9TZP3		1257
Plasmodium fragile	4.2e-109	51.7	A0A0D9QR21		1270
Homo sapiens	-----	-----	Q9P2J5	2WFD	1176
Escherichia coli	-----	-----	P07813	4AS1,4ARI,4ARC	860
**Arginine aminoacyl synthetase**					
Plasmodium falciparum	0	100.0	Q8I5M2	5JLD	590
Plasmodium malariae	0	76.2	A0A1D3TEU0		588
Plasmodium vivax	0	76.4	A0A0J9TME5		591
Plasmodium ovale	0	74.7	A0A1C3L5C5		588
Plasmodium fragile	0	74.4	A0A0D9QJI4		594
Plasmodium knowlesi	0	75.0	B3LCI0		588
Plasmodium berghei	0	73.0	A0A077XII2		588
Plasmodium yoelii	0	72.4	V7PSR5		588
Homo sapiens	1.8e-125	36.7	P54136	4Q2T, 4Q2X, ZAJ,4Q2Y	660
Cryptosporidium andersoni	2.00E-168	42.0	B6A914		603
Escherichia coli	1.4E-104	32.6	P11875	4OBY	577
Saccharomyces cerevisiae	1.9E-46	28.3	Q05506	1F7V,1BS2,1F7U	607
**Valine aminoacyl synthetase**					
Plasmodium falciparum	0	100.0	Q8IKL5		1090
Plasmodium malariae	0	78.8	A0A1D3SPV8		1089
Plasmodium yoelii	0	77.8	V7PVF4		1077
Plasmodium fragile	0	78.3	A0A0D9QNU6		1046
Plasmodium vivax	0	77.1	A0A0J9TS21		1071
Plasmodium knowlesi	0	76.6	B3L9E5		1070
Plasmodium berghei	0	76.1	A0A077XIK7		1077
Plasmodium ovale	0	75.5	A0A1A8WWY3		1138
Homo sapiens	------	-----	P26640		1264
Cryptosporidium andersoni	0	47.0	A0A1J4MSQ0		1046
Cryptosporidium parvum	0	45.0	Q9Y1L6		1050
Dictyostelium discoideum	0	43.1	Q86KU2		10072
**Glutamate aminoacyl synthetase**					
Plasmodium falciparum	0	100	Q8IDK7		863
Plasmodium fragile	0	64.5	A0A0D9QKM1		802
Plasmodium ovale	0	65.6	A0A1C3KUT6		789
Plasmodium knowlesi	0	64.6	B3L8A1		802

Plasmodium vivax	0	63.7	A0A0J9SRD3		787
Plasmodium yoelii	0	64.0	V7PWS4		804
Plasmodium berghei	0	64.2	A0A077XJI3		803
Plasmodium malariae	0	61.6	A0A1C3L0G3		802
Homo sapiens	5.6e-151	39.2	P07814	5A5H,5A1N,4K87,5BMU	1512
Toxoplasma gondii	0	49.0	S8F214		849
Cryptosporidium andersoni	0	57.0	A0A1J4MSL8		562
Saccharomyces cerevisiae	9.3E-159	38.5	P46655		708
**Isoleucine aminoacyl synthetase**					
Plasmodium falciparum	0	100.0	Q8IDZ9		1272
Plasmodium ovale	0	71.8	A0A1C3KVD6		1189
Plasmodium malariae	0	68.0	A0A1C3L0V5		1365
Plasmodium yoelii	0	68.8	Q7RNG8		1294
Plasmodium berghei	0	69.8	A0A077XJ63		1275
Plasmodium knowlesi	0	68.4	B3L8Q4		1190
Plasmodium fragile	0	66.5	A0A0D9QHQ1		1082
Plasmodium vivax	0	67.5	A0A0J9VFT0		1206
Homo sapiens	----	-----	P41252		1262
Toxoplasma gondii	0	44.0	A0A086QSP8		1242
Cryptosporidium ubiquitum	0	48.0	A0A1J4MJA8		1099
Escherichia coli	8.90E-07	24.6	P07813		860
**Tryptophan aminoacyl synthetase**					
Plasmodium falciparum	0	100.0	Q8IDW3	4J76,4J75,4JFA	632
Plasmodium knowlesi	0	79.9	B3L8M2		651
Plasmodium vivax	0	80.5	A0A0J9S986		645
Plasmodium ovale	0	79.6	A0A1C3KV46		648
Plasmodium fragile	0	78.4	A0A0D9QIG0		658
Plasmodium malariae	0	75.0	A0A1C3L0K6		679
Plasmodium yoelii	0	74.6	V7PT19		666
Plasmodium berghei	0	74.4	A0A077XJ93		662
Homo sapiens	2.5e-130	50.4	P23381		471
Toxoplasma gondii	0	44.0	A0A086QHU2		689
Cryptosporidium andersoni	1.00E-168	43.0	A0A1J4MNU6		598
Escherichia coli	0.13	37.1	P00954		334
Saccharomyces cerevisiae	1E-123	48.5	Q12109		432
**Glutamine aminoacyl synthetase**					
Plasmodium falciparum	0	100.0	Q8IE10		918
Plasmodium malariae	0	59.4	A0A1A8W8L1		922
Plasmodium yoelii	0	60.9	V7PXA4		887
Plasmodium ovale	0	65.9	A0A1C3KVA4		844
Plasmodium berghei	0	59.8	A0A077XKG1		852
Plasmodium knowlesi	0	59.4	B3L8R5		863
Plasmodium vivax	0	57.7	A0A1G4H184		878
Plasmodium fragile	0	64.8	A0A0D9QKQ9		847
Homo sapiens	2.2e-108	38.3	P47897	4YE6,4YE9,4YE8	775
Toxoplasma gondii	0	52.0	A0A086QTE2		860
Saccharomyces cerevisiae	6,5E-112	35.9	P13188		809
Escherichia coli	4.3E-172	44.8	P00962		554
**Tyrosine aminoacyl synthetase**					
Plasmodium falciparum	0	100.0	Q8IAR7	3VGJ	373
Plasmodium malariae	0	85.8	A0A1A8VP49		372
Plasmodium knowlesi	0	80.5	B3KZ67		385
Plasmodium berghei	0	81.8	A0A077XEQ8		372
Plasmodium fragile	0	80.8	A0A0D9QTW8		380
Plasmodium vivax	0	80.5	A0A0J9U5P1		380
Plasmodium ovale	0	77.5	A0A1A8VJM4		374
Plasmodium yoelii	0	82.0	A0A078K7H1		372
Homo sapiens	3e-403	30.0	P54577	1Q11,4QBT,4Q93	528
Saccharomyces cerevisiae	7.3E-31	30.7	P36421		394
Bacillus subtilis	0,0000024	23.6	P21656		330
**Cysteine aminoacyl synthetase**					
Plasmodium falciparum	0	100.0	Q8IJP3		677
Plasmodium malariae	0	76.0	A0A1D3PAE8		678
Plasmodium vivax	0	71.9	A0A0D9QHH7		699

Plasmodium fragile	0	71.6	A0A0J9TZ62		686
Plasmodium knowlesi	0	71.0	B3L3U5		695
Plasmodium berghei	0	73.7	A0A077XEI5		668
Plasmodium ovale	0	71.8	A0A1D3TGR8		704
Plasmodium yoelii	0	73.1	A0A078KDT0		665
Homo sapiens	-----	----	P49589		748
Toxoplasma gondii	0	52.0	S8EQ47		898
Cryptosporidium hominis	0	50.0	A0A0S4TGX8		581
Escherichia coli	4.5E-73	41.5	P21888		461

Additional file 1 - Table 2: Class II aminoacyl synthetase sequence details

**Species/Protein**	**E-value**	**Identity**	**Accession code**	**PDB ID**	**Coverage**
**Threonine aminoacyl synthetase**					
Plasmodium falciparum	0	100.0	Q8IIA4		1013
Plasmodium ovale	0	65.8	A0A1D3TI17		903
Plasmodium malariae	0	65.8	A0A1A8VYM6		890
Plasmodium knowlesi	0	60.3	B3L565		888
Plasmodium fragile	0	64.8	A0A0D9QSY6		875
Plasmodium berghei	0	58.9	A0A077XDL8		890
Plasmodium yoelii	0	57.9	A0A078K4L5		935
Plasmodium vivax	0	65.3	A0A1G4GXN4		924
Homo sapiens	9.9e-176	53.3	P26639	4TTV,4P3N,4HWT	723
Toxoplasma gondii	0	50.0	A0A151H0C0		846
Staphylococcus aureus	1.1e-100	38.3	Q8NW68	1NYQ,1NYR	645
Escherichia coli	3.7e-108	41.3	P0A8M3	1EVL,1TKE,1TKY,4HWS,1QF6	642
Cryptosporidium andersoni	0	49.0	A0A1J4MNI4		743
**Alanine aminoacyl synthetase**					
Plasmodium falciparum	0	100.0	Q8ID31		1408
Plasmodium ovale	0	72.6	A0A1A8W127		1274
Plasmodium malariae	0	70.7	A0A1A8W685		1182
Plasmodium knowlesi	0	71.8	B3L6U2		1180
Plasmodium fragile	0	71.7	A0A0D9QQK3		1184
Plasmodium berghei	0	63.5	A0A077XGH3		1218
Plasmodium yoelii	0	64.0	Q7RK23		1218
Plasmodium vivax	0	72.4	A0A1G4GZF2		1197
Homo sapiens	0	36.8	P49588	4XEM, 5T76,4XEO,5T5S	968
Toxoplasma gondii	0	44.0	V4Z4M5		1280
Cryptosporidium hominis	0	44.0	A0A0S4TIH6		**989**
Cryptosporidium ubiquitum	0	-----	A0A1J4MG14		991
Escherichia coli	-----	-----	P00957		876
**Serine aminoacyl synthetase**					
Plasmodium falciparum	0	100.0	Q8IBS3		539
Plasmodium malariae	0	68.6	A0A1A8VNA1		571
Plasmodium fragile	0	70.5	A0A0D9QLI8		555
Plasmodium knowlesi	0	70.4	B3L070		597
Plasmodium ovale	0	69.5	A0A1C3KNB7		560
Plasmodium vivax	0	68.7	A0A1G4GS18		616
Plasmodium yoelii	0	69.1	V7PG45		534
Plasmodium berghei	0	68.8	A0A077XBK7		536
Homo sapiens	3.00E-120	38.7	P49591	4L87,3VBB,4RQF,4RQE	514
Toxoplasma gondii	2.00E-169	46.0	S7WG10		482
Cryptosporidium parvum	9.00E-145	44.0	Q5CVB3		454
Candida albicans	-----	-----	Q9HGT6		462
Trypanosoma brucei	-----	-----	Q384V4		479
**Lysine aminoacyl synthetase**					
Plasmodium falciparum	0	100.0	Q8IDJ8	4PG3,4H02	583
Plasmodium malariae	0	82.8	A0A1C3L068		583
Plasmodium knowlesi	0	81.4	B3L892		585
Plasmodium fragile	0	81.9	A0A0D9QKL2		585
Plasmodium vivax	0	81.0	A0A0J9WC33		585

Plasmodium berghei	0	80.4	A0A077XH86		578
Plasmodium yoelii	0	79.8	V7PTW4		581
Plasmodium ovale	0	80.0	A0A1D3TJZ1		601
Homo sapiens	0	51.6	Q15046-2	4YCU,4DPG	597
Toxoplasma gondii	0	60.0	A0A125YM18		658
Escherichia coli	2.8E-114	42.7	P0A8N5		505
**Glycine aminoacyl synthetase**					
Plasmodium falciparum	0	100.0	Q8ILP6		889
Plasmodium malariae	0	64.0	A0A1A8W9X0		884
Plasmodium knowlesi	0	64.0	B3LAH4		830
Plasmodium berghei	0	62.1	A0A077YEW9		812
Plasmodium yoelii	0	61.1	V7PKZ3		806
Plasmodium fragile	0	60.8	A0A0D9QRV8		857
Plasmodium ovale	0	61.2	A0A1A8W3I2		806
Plasmodium vivax	0	63.3	A0A0J9S894		842
Homo sapiens	8.2e-137	43.9	P41250	2Q5I, 5E6M	739
Cryptosporidium ubiquitum	0	48.0	A0A1J4MED4		655
Toxoplasma gondii	0	50.0	S7UYX0		771
Thermus thermophillus	1.6E-21	30.3	P56206		506
**Histidine aminoacyl synthetase**					
Plasmodium falciparum	0	100.0	Q8IL22		1132
Plasmodium ovale	0	59.4	A0A1D3TLI4		1074
Plasmodium malariae	0	58.4	A0A1D3SQX7		1069
Plasmodium yoelii	0	57.2	V7PBA0		1032
Plasmodium berghei	0	57.7	A0A077XI40		1068
Plasmodium vivax	0	55.9	A0A0J9TR61		1067
Plasmodium knowlesi	0	55.9	B3L9W6		1056
Plasmodium fragile	0	54.7	A0A0D9QPZ2		1056
Homo sapiens	1.6e-89	48.0	P12081	4G84,4PHC	**509**
Cryptosporidium parvum	4.00E-103	46.0	Q5CVH7		977
Trypanosoma cruzi	1.3E-22	27.3	Q4DA54		478
**Aspartate aminoacyl synthetase**					
Plasmodium falciparum	0	100.0	Q8I2B1		626
Plasmodium ovale	0	79.6	A0A1D3KWQ4		648
Plasmodium malariae	0	71.8	A0A1D3JKS8		640
Plasmodium vivax	0	70.8	A0A0J9TKH9		631
Plasmodium knowlesi	0	73.5	B3KZP7		628
Plasmodium yoelii	0	75.8	V7PM27		681
Plasmodium berghei	0	77.3	A0A077X659		686
Plasmodium fragile	0	76.6	A0A0D9QTT7		582
Homo sapiens	2.8e-133	48.0	P14868	4J15	501
Toxoplasma gondii	3.00E-156	45.0	S8F9A0		773
Cryptosporidium parvum	7.00E-153	49.0	A3FPN1		532
Escherichia coli	1.8E-11	25.6	P21889		590
Saccharomyces cerevisiae	1.3E-127	41.0	P04802		557
**Phenylalanine aminoacyl synthetase**					
Plasmodium falciparum	0	100.0	Q8I5A1		556
Plasmodium berghei	0	48.5	A0A077XKI5		608
Plasmodium malariae	0	48.6	A0A1D3TF68		635
Plasmodium yoelii	2.00E-172	48.5	Q7RFF0		589
Plasmodium knowlesi	1.7e-169	45.1	A0A1A7VGI2		563
Plasmodium ovale	2.5e-166	43.9	A0A1A9A0Y8		627
Plasmodium vivax	3.6e-151	42.8	A0A1K9YHP8		539
Plasmodium fragile	1.7e-160	43.6	A0A0D9QSX6		561
Homo sapiens	5.1e-13	25.5	O95363	5MGW,3TEG,3TEG	451
**Asparagine aminoacyl synthetase**					
Plasmodium falciparum	0	100.0	O96198		610
Plasmodium ovale	0	76.7	A0A1A8YJG9		612
Plasmodium fragile	0	76.9	A0A0D9QIE9		581
Plasmodium knowlesi	0	76.4	B3L0N7		586
Plasmodium vivax	0	75.7	A0A0J9TZK6		584
Plasmodium malariae	0	76.1	A0A1A8VU61		595
Plasmodium berghei	0	76.7	A0A077X7L3		575
Plasmodium yoelii	0	75.5	V7PCK4		576
Toxoplasma gondii	0	56.0	S8EYC0		676

Cryptosporidium ubiquitum	0	50.0	A0A1J4M9Q6		499
Salmonella typhi	1.3E-150	46.1	P58696		466
Homo sapiens	2.9E-38	32.6	O43776	4ZYA	548
**Proline aminoacyl synthetase**					
Plasmodium falciparum	----	100.0	Q8I5R7	4WI1,4OLF,4NCX,4YDQ	746
Plasmodium malariae	0	81.4	A0A1A8WCF8		747
Plasmodium ovale	0	78.1	A0A1A8X0X1		776
Plasmodium knowlesi	0	78.0	A0A1A7VHC7		715
Plasmodium yoelii	0	76.8	V7PQB2		728
Plasmodium fragile	0	78.9	A0A0D9QI05		748
Plasmodium vivax	0	78.3	A0A0J9SMR8		773
Plasmodium berghei	0	76.2	A0A077XI12		755
Homo sapiens	1.00E-167	48.1	P07814	4K86	1512
Toxoplasma gondii	0	51.0	S7V2A6		830
Cryptosporidium andersoni	0	50.0	A0A1J4MHX7		691
Thermus thermophillus	4.4E-120	44.3	Q5SM28		477
