# Supplementary material for: Aminoacyl tRNA synthetases as malarial drug targets: a comparative bioinformatics study
Source: Malar J. 2019 Feb 6;18:34. doi: 10.1186/s12936-019-2665-6 (PMC6366043; doi:10.1186/s12936-019-2665-6)
Supplement: Supplementary file 2 — Additional file 2. Homology model validation results obtained for Verify 3D, QMEAN and ProSA webservers. The z-DOPE scores for each model and the templates used for modelling are also shown. [file 12936_2019_2665_MOESM2_ESM.pdf]

Additional file 2: Model validation results from Vetify3D, PROSA and QMEAN for TyrRS, TrpRS, ArgRS, ProRS, LysRS and MetRS homology models.

Additional file 2A: Model validation results for TyrRS

Model	Verify3D score	PROSA	QMEAN Value	z-DOPE Score
5USF.pdb	100.0	-13.23	-0.47	-1.20
PyTyrRS	81.99	-7.9	-3.34	-1.14
PvTyrRS	85.26	-7.55	-2.63	-0.83
PoTyrRS	81.02	-6.15	-3.68	-0.93
PmTyrRS	84.00	-8.42	-2.25	-1.28
PkTyrRS	92.01	-8.17	-2.49	-1.23
PfrTyrRS	94.08	-8.53	-3.35	-1.16
PbTyrRS	80.38	-7.75	-3.21	-1.07
HsTyrRS	88.01	-8.2	-1.21	-1.54

Additional file 2B: Model validation results for TrpRS

Model	Verify3D score	PROSA	QMEAN Value	z-DOPE Score
4J75.pdb	99.73	-8.86	-0.40	-2.47
PfTrpRS	99.22	-9.25	-0.52	-2.12
PvTrpRS	91.71	-6.77	-3.12	-1.64
PoTrpRS	99.74	-8.93	-0.98	-1.92
PmTrpRS	87.78	-7.43	-3.77	-1.34
PkTrpRS	97.28	-6.82	-2.33	-2.72
PfrTrpRS	93.92	-6.85	-3.74	-3.95
PyTrpRS	96.53	-5.74	-2.65	-2.90
PbTrpRS	94.79	-6.06	-3.71	-1.53
1R6T.pdb	90.55	-10.61	-0.24	-1.86
HsTrpRS	89.23	-9.47	-1.91	-1.41

Additional file 2C: Model validation results for ArgRS

Model	Verify3D score	PROSA	QMEAN Value	z-DOPE Score
5JLD.pdb	90.71	-9.85	-1.26	-1.80
PyArgRS	92.08	-11.37	-2.29	-1.30
PvArgRS	94.86	-10.95	-1.68	-1.30
PoArgRS	90.53	-11.67	-1.70	-1.31
PmArgRS	96.73	-11.62	-1.87	-1.27
PkArgRS	93.63	-11.28	-1.77	-1.36
PfrArgRS	94.21	-10.75	-2.25	-1.30
PbArgRS	93.80	-11.22	-2.28	-1.38
PfArgRS	96.91	-11.41	-1.32	-1.31
4ZAJ.pdb	85.96	-11.79	-0.48	-1.76
HsArgRS	96.26	-11.91	-0.91	-1.51

Additional file 2D: Model validation results for ProRS

Model	Verify3D score	PROSA	QMEAN Value	z-DOPE Score
4NCX.pdb	93.30	-8.50	-0.87	-1.47
PfProRS	95.37	-9.97	-1.52	-1.29
PyProRS	87.61	-9.25	-2.93	-1.23
PvProRS	87.60	-8.86	-3.53	-1.01
PoProRS	87.70	-8.68	-3.92	-0.96
PmProRS	83.93	-8.48	-3.12	-1.08
PkProRS	91.88	-8.72	-1.74	-1.28
PfrProRS	81.55	-8.77	-3.14	-1.04
PbProRS	86.06	-9.25	-2.41	-1.11
HsProRS	89.74	-9.86	-1.09	-0.84

Additional file 2E: Model validation results for LysRS

Model	Verify3D score	PROSA	QMEAN Value	z-DOPE Score
4DPG.pdb	88.53	-9.36	-0.62	-0.97
PfLysRS	99.94	-9.01	-1.08	-1.01
PyLysRS	82.24	-9.56	-1.09	-0.78
PvLysRS	77.60	-10.01	-0.86	-0.81
PoLysRS	84.13	-9.52	-1.18	-0.86
PmLysRS	83.00	-9.76	-1.58	-0.70
PkLysRS	90.20	-9.81	-1.10	-0.89
PfrLysRS	92.00	-9.83	-0.92	-0.80
PbLysRS	84.40	-9.71	-1.37	-0.79
HsLysRS	83.83	-9.73	-0.39	-0.84

Additional file 2F: Model validation results for MetRS

Model	Verify3D score	PROSA	QMEAN Value	z-DOPE score
4DLP.pdb	100.00	-11.78	0.56	-1.97
PfMetRS	-10.30	-10.30	-2.39	-1.31
PvMetRS	89.02	-10.06	-2.49	-1.34
PyMetRS	82.29	-10.78	-2.64	-1.33
PoMetRS	86.63	-9.90	-3.29	-1.38
PmMetRS	83.03	-10.07	-2.89	-1.29
PkMetRS	88.82	-10.06	-2.65	-1.36
PfrMetRS	84.70	-10.22	-3.04	-1.32
PbMetRS	87.50	-10.58	-3.08	-1.31
HsMetRS	92.41	-10.85	-4.01	-1.19
