# Supplementary material for: Aminoacyl tRNA synthetases as malarial drug targets: a comparative bioinformatics study
Source: Malar J. 2019 Feb 6;18:34. doi: 10.1186/s12936-019-2665-6 (PMC6366043; doi:10.1186/s12936-019-2665-6)
Supplement: Supplementary file 3 — Additional file 3. Motifs discovered for the 20 aminoacyl tRNA synthetase families using MEME software. The default motif width of 6–50 residues was used. The Mast tool was used to identify overlapping motifs. The number of motifs run for each family varied and motif conservation was presented as number of sites divided by total number of class sequences and results displayed as heatmaps. Motif conservation increases from blue to red. [file 12936_2019_2665_MOESM3_ESM.pdf]

Additional file 3: Motifs discovered for the 20 aminoacyl synthetase families using MEME software. The number of motifs run for each family varied and motif conservation was calculated as number of sites/total number of class sequences and results displayed as heatmaps as shown below. Motif conservation increases from blue to red.

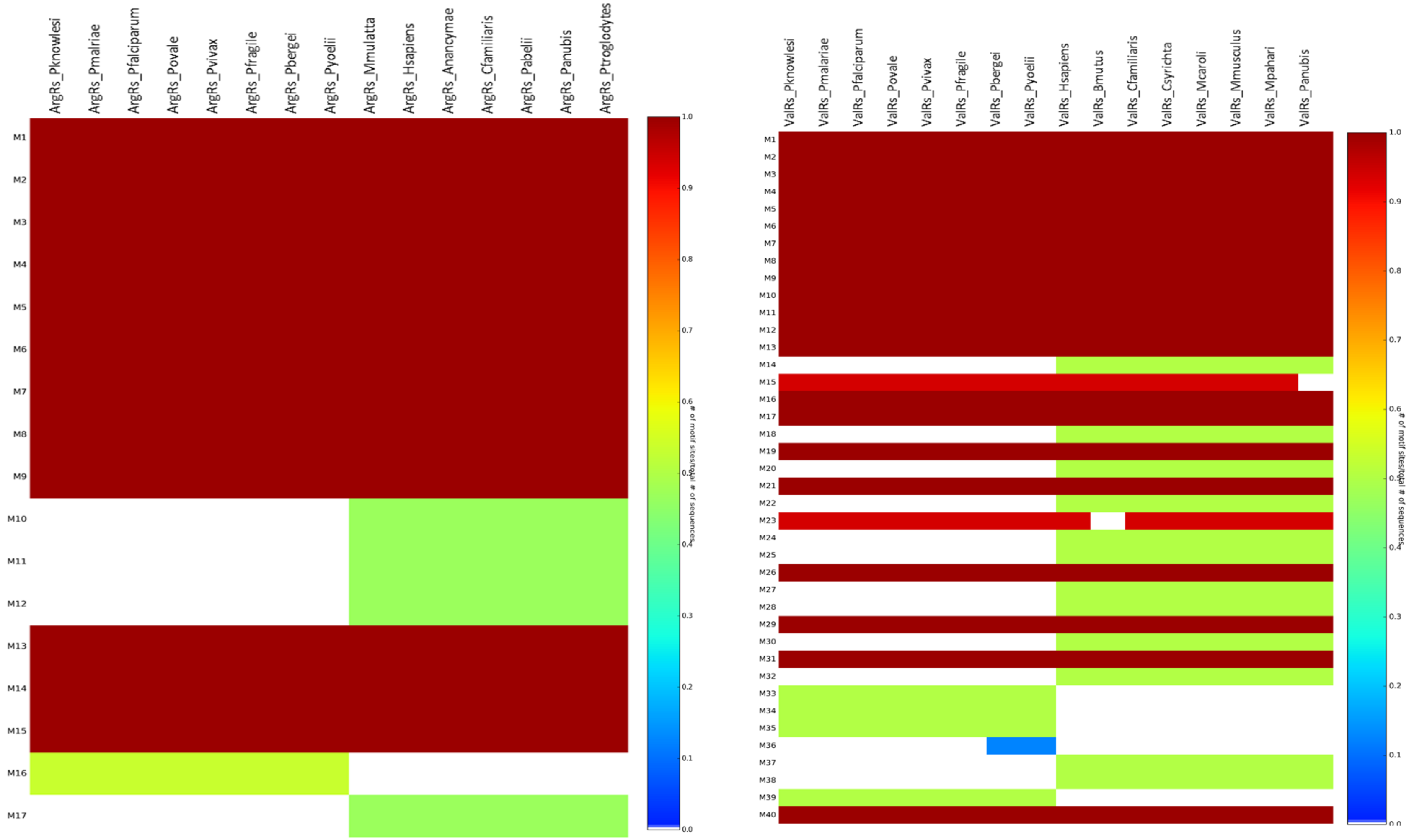

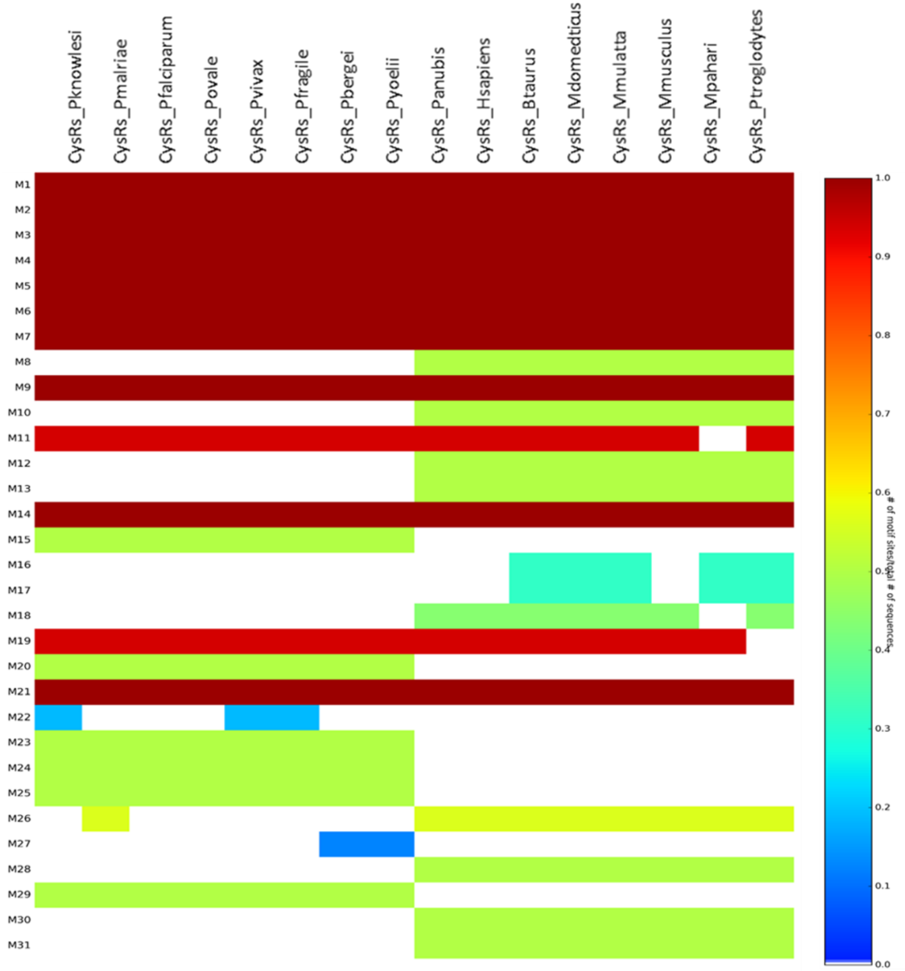
CysRs_Pknowlesi
CysRs_Pmalriae
CysRs_Pfalciparum
CysRs_Povale
CysRs_Pvivax
CysRs_Pfragile
CysRs_Pbergei
CysRs_Pyoelii
CysRs_Panubis
CysRs_Hsapiens
CysRs_Btaurus
CysRs_Mdomedticus
CysRs_Mmulatta
CysRs_Mmusculus
CysRs_Mpahari
CysRs_Ptroglodytes
M1
M2
M3
M4
M5
M6
M7
M8
M9
M10
M11
M12
M13
M14
M15
M16
M17
M18
M19
M20
M21
M22
M23
M24
M25
M26
M27
M28
M29
M30
M31
1.0
0.9
0.8
0.7
0.6
0.4
0.3
0.2
0.1
0.0
# of motif sites/total # of sequences

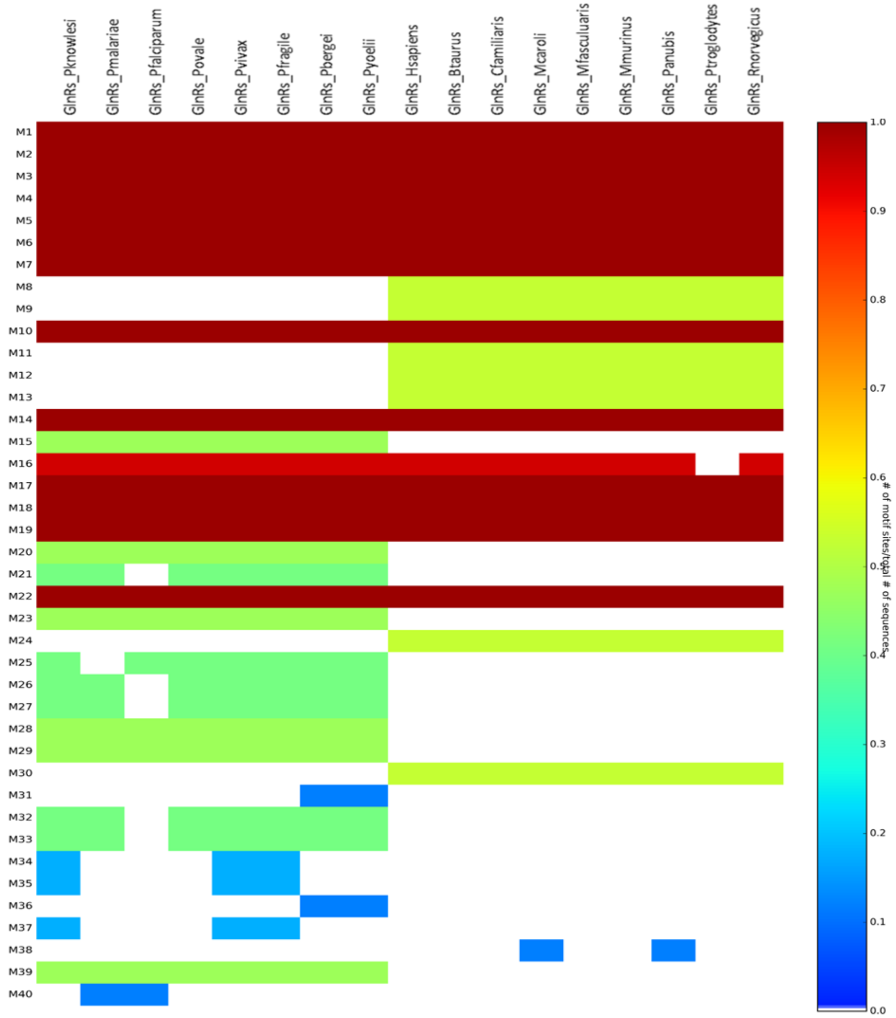
GlnRs_Pknowlesi
GlnRs_Pmalariae
GlnRs_Pfalciparum
GlnRs_Povale
GlnRs_Pvivax
GlnRs_Pfragile
GlnRs_Pbergei
GlnRs_Pyoelii
GlnRs_Hsapiens
GlnRs_Btaurus
GlnRs_Cfamiliaris
GlnRs_Mcaroli
GlnRs_Mfasculuaris
GlnRs_Mmurinus
GlnRs_Panubis
GlnRs_Ptroglodytes
GlnRs_Rnorvegicus
M1
M2
M3
M4
M5
M6
M7
M8
M9
M10
M11
M12
M13
M14
M15
M16
M17
M18
M19
M20
M21
M22
M23
M24
M25
M26
M27
M28
M29
M30
M31
M32
M33
M34
M35
M36
M37
M38
M39
M40
1.0
0.9
0.8
0.7
0.6
0.4
0.3
0.2
0.1
0.0
# of motif sites/total # of sequences

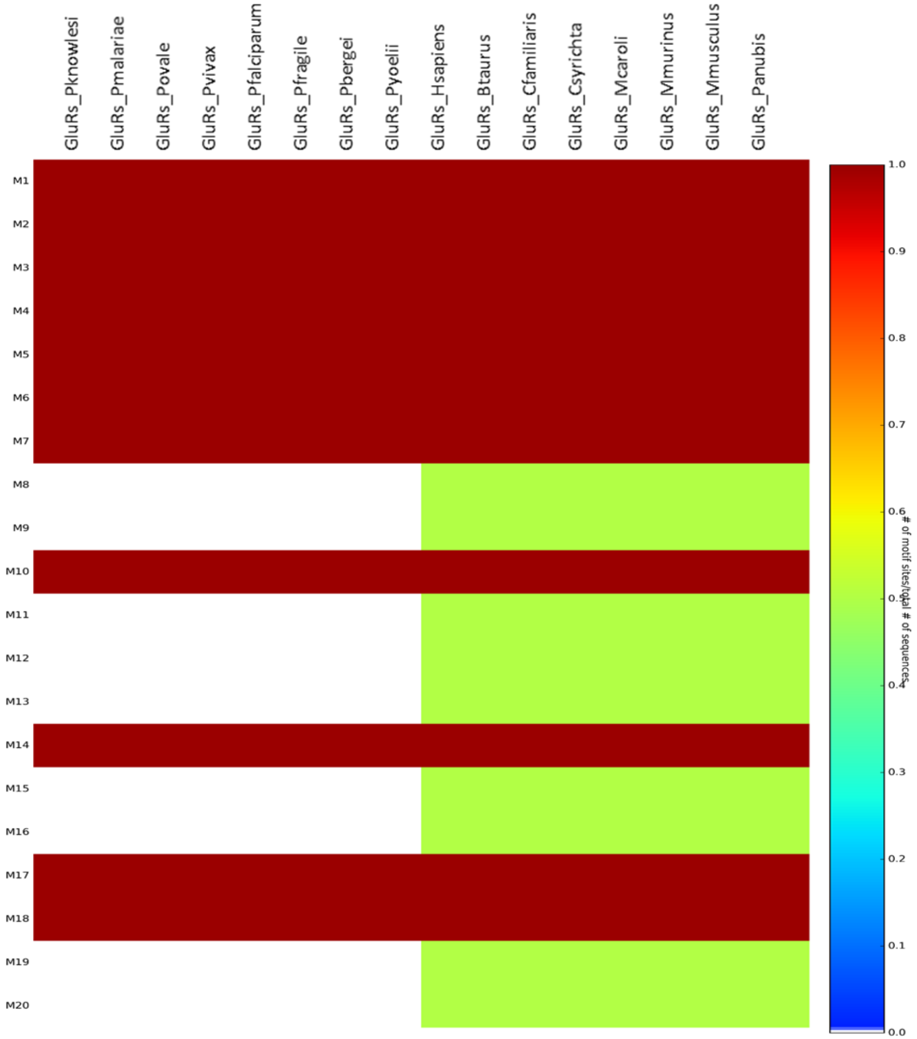
GluRs_Pknowlesi
GluRs_Pmalariae
GluRs_Povale
GluRs_Pvivax
GluRs_Pfalciparum
GluRs_Pfragile
GluRs_Pbergei
GluRs_Pyoelii
GluRs_Hsapiens
GluRs_Btaurus
GluRs_Cfamiliaris
GluRs_Csyrichta
GluRs_Mcaroli
GluRs_Mmurinus
GluRs_Mmusculus
GluRs_Panubis
M1
M2
M3
M4
M5
M6
M7
M8
M9
M10
M11
M12
M13
M14
M15
M16
M17
M18
M19
M20
1.0
0.9
0.8
0.7
0.6
0.5
0.4
0.3
0.2
0.1
0.0
# of motif sites/total # of sequences

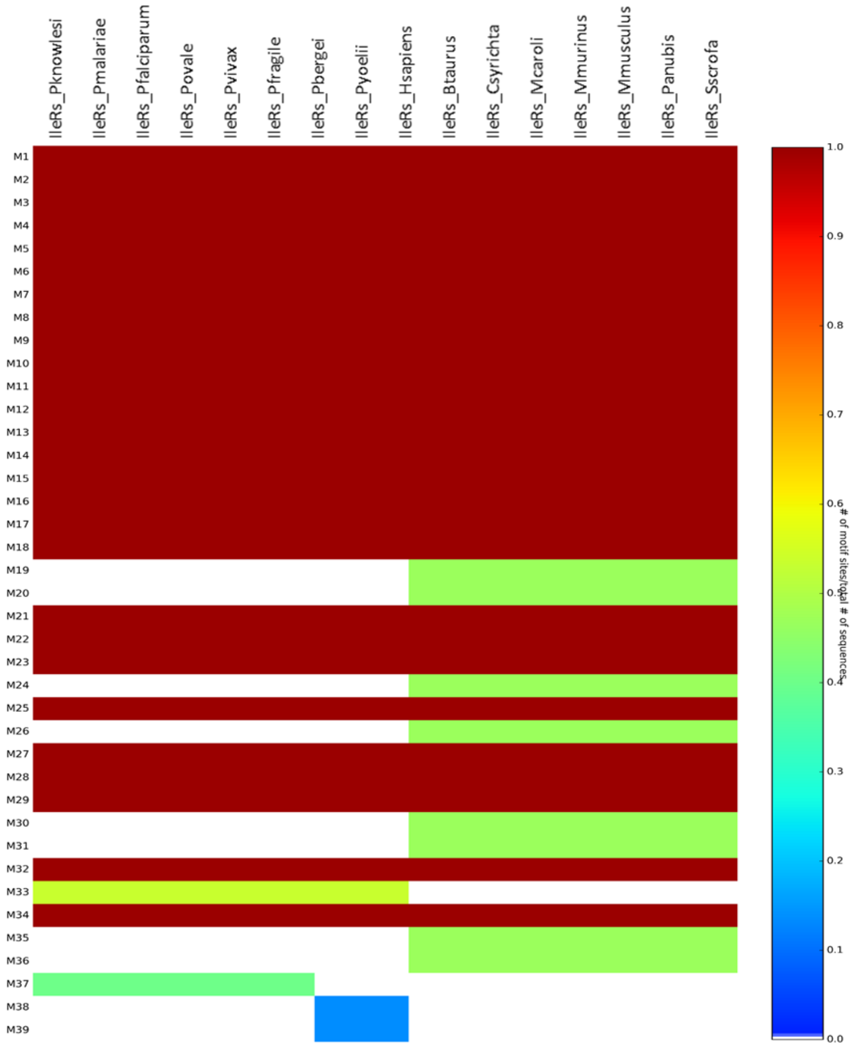
IleRs_Pknowlesi
IleRs_Pmalariae
IleRs_Pfalciparum
IleRs_Povale
IleRs_Pvivax
IleRs_Pfragile
IleRs_Pbergei
IleRs_Pyoelii
IleRs_Hsapiens
IleRs_Btaurus
IleRs_Csyrichta
IleRs_Mcaroli
IleRs_Mmurinus
IleRs_Mmusculus
IleRs_Panubis
IleRs_Sscrofa
M1
M2
M3
M4
M5
M6
M7
M8
M9
M10
M11
M12
M13
M14
M15
M16
M17
M18
M19
M20
M21
M22
M23
M24
M25
M26
M27
M28
M29
M30
M31
M32
M33
M34
M35
M36
M37
M38
M39
1.0
0.9
0.8
0.7
0.6
0.5
0.4
0.3
0.2
0.1
0.0
# of motif sites/total # of sequences

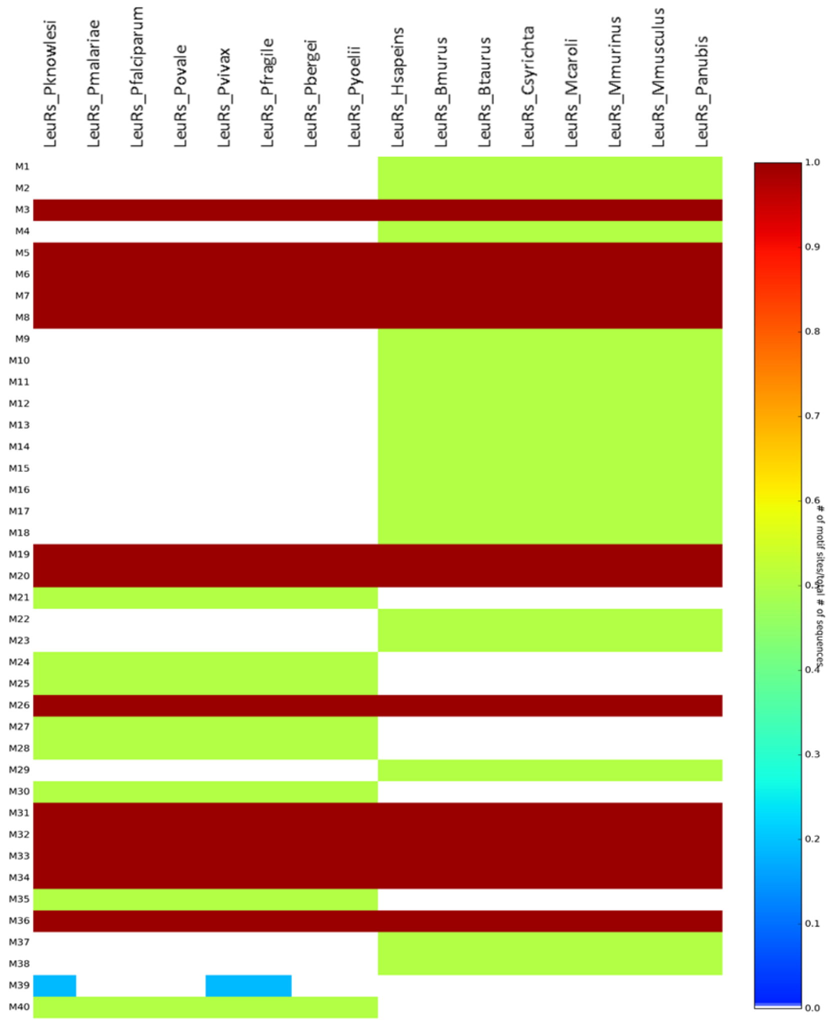
LeuRs_Pknowlesi
LeuRs_Pmalariae
LeuRs_Pfalciparum
LeuRs_Povale
LeuRs_Pvivax
LeuRs_Pfragile
LeuRs_Pbergei
LeuRs_Pyoelii
LeuRs_Hsapeins
LeuRs_Bmurus
LeuRs_Btaurus
LeuRs_Csyrichta
LeuRs_Mcaroli
LeuRs_Mmurinus
LeuRs_Mmusculus
LeuRs_Panubis
M1 M2 M3 M4 M5 M6 M7 M8 M9 M10 M11 M12 M13 M14 M15 M16 M17 M18 M19 M20 M21 M22 M23 M24 M25 M26 M27 M28 M29 M30 M31 M32 M33 M34 M35 M36 M37 M38 M39 M40
1.0 0.9 0.8 0.7 0.6 0.4 0.3 0.2 0.1 0.0
# of motif sites/total # of sequences

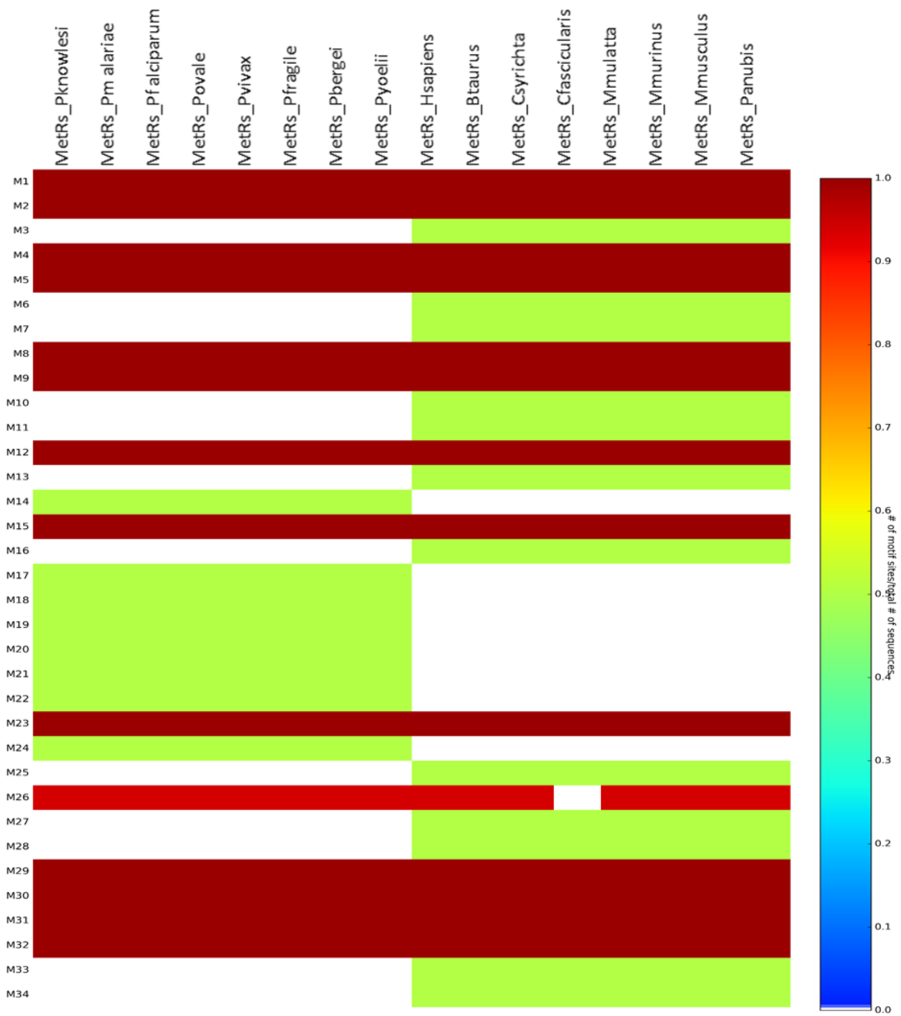
MetRs_Pknowlesi
MetRs_Pm alariae
MetRs_Pf alciparum
MetRs_Povale
MetRs_Pvivax
MetRs_Pfragile
MetRs_Pbergei
MetRs_Pyoelii
MetRs_Hsapiens
MetRs_Btaurus
MetRs_Csyrichta
MetRs_Cfascicularis
MetRs_Mmulatta
MetRs_Mmurinus
MetRs_Mmusculus
MetRs_Panubis
M1 M2 M3 M4 M5 M6 M7 M8 M9 M10 M11 M12 M13 M14 M15 M16 M17 M18 M19 M20 M21 M22 M23 M24 M25 M26 M27 M28 M29 M30 M31 M32 M33 M34
1.0 0.9 0.8 0.7 0.6 0.4 0.3 0.2 0.1 0.0
# of motif sites/total # of sequences

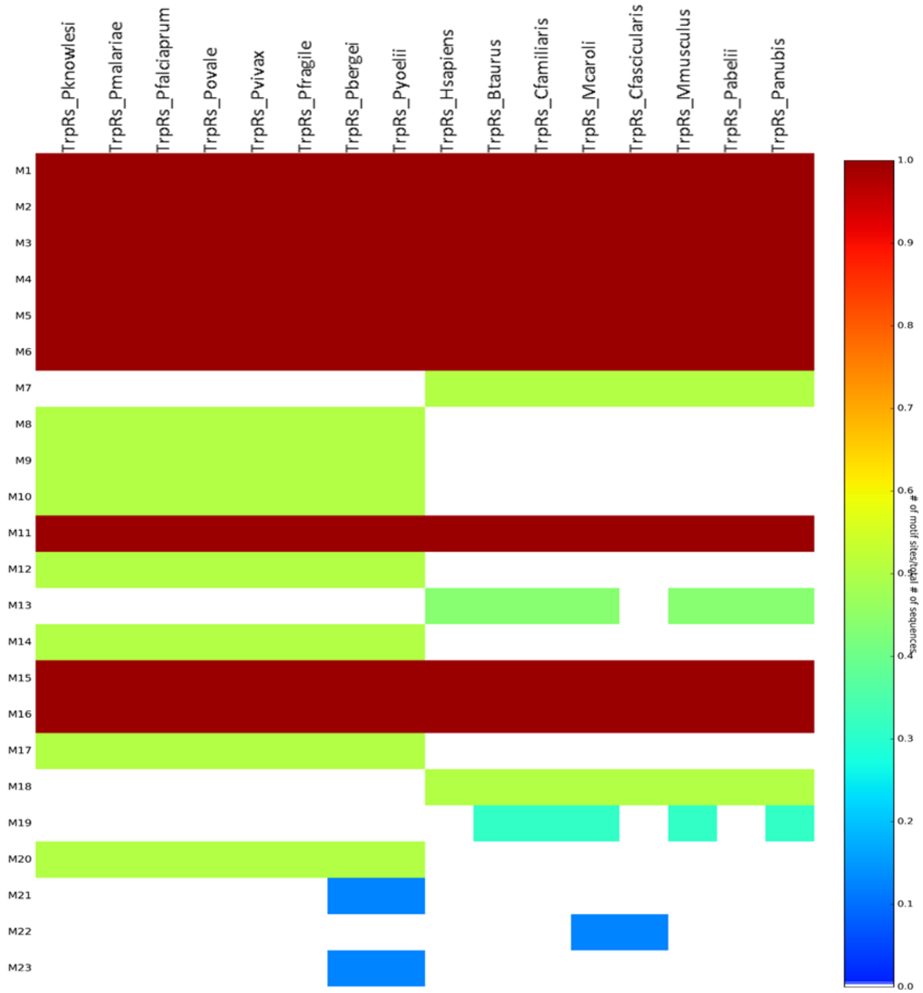
TrpRs_Pknowlesi
TrpRs_Pmalariae
TrpRs_Pfalciaprum
TrpRs_Povale
TrpRs_Pvivax
TrpRs_Pfragile
TrpRs_Pbergei
TrpRs_Pyoelii
TrpRs_Hsapiens
TrpRs_Btaurus
TrpRs_Cfamiliaris
TrpRs_Mcaroli
TrpRs_Cfascicularis
TrpRs_Mmusculus
TrpRs_Pabelii
TrpRs_Panubis
M1
M2
M3
M4
M5
M6
M7
M8
M9
M10
M11
M12
M13
M14
M15
M16
M17
M18
M19
M20
M21
M22
M23
1.0
0.9
0.8
0.7
0.6
0.4
0.3
0.2
0.1
0.0
# of motif sites/total # of sequences

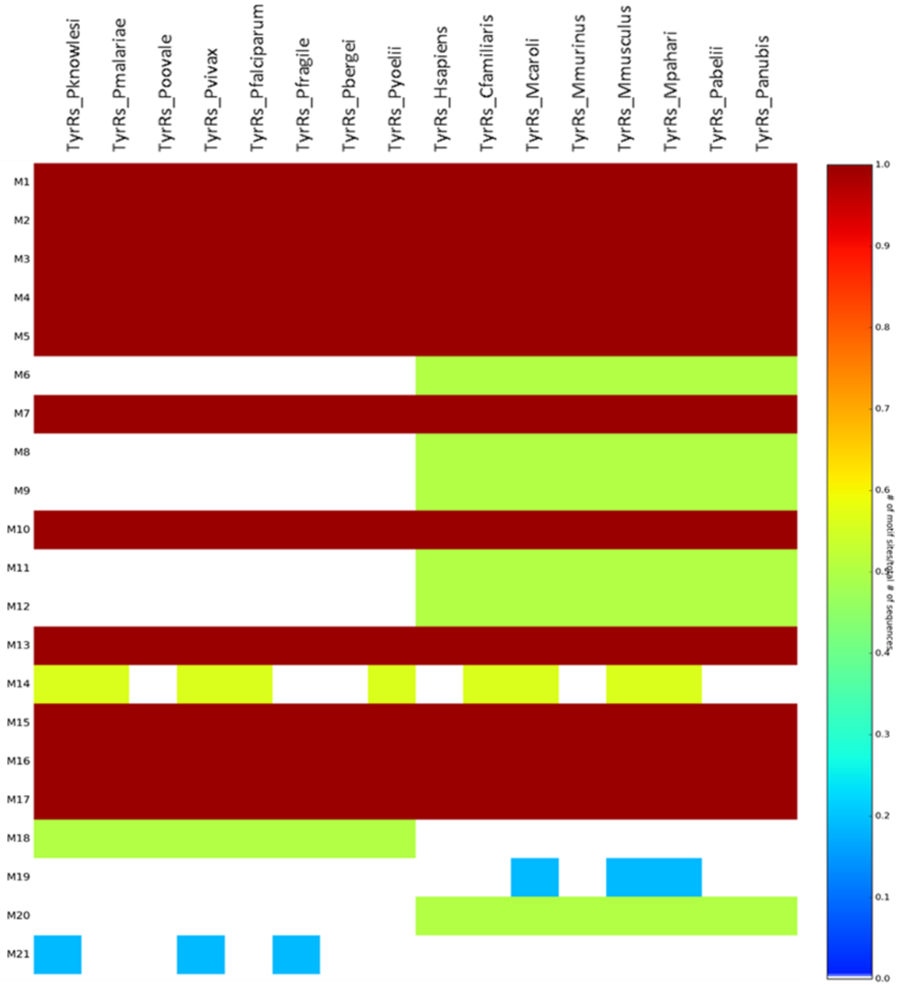
TyrRs_Pknowlesi
TyrRs_Pmalariae
TyrRs_Poovale
TyrRs_Pvivax
TyrRs_Pfalciparum
TyrRs_Pfragile
TyrRs_Pbergei
TyrRs_Pyoelii
TyrRs_Hsapiens
TyrRs_Cfamiliaris
TyrRs_Mcaroli
TyrRs_Mmurinus
TyrRs_Mmusculus
TyrRs_Mpahari
TyrRs_Pabelii
TyrRs_Panubis
M1
M2
M3
M4
M5
M6
M7
M8
M9
M10
M11
M12
M13
M14
M15
M16
M17
M18
M19
M20
M21
1.0
0.9
0.8
0.7
0.6
0.3
0.2
0.1
0.0
# of motif sites/total # of sequences

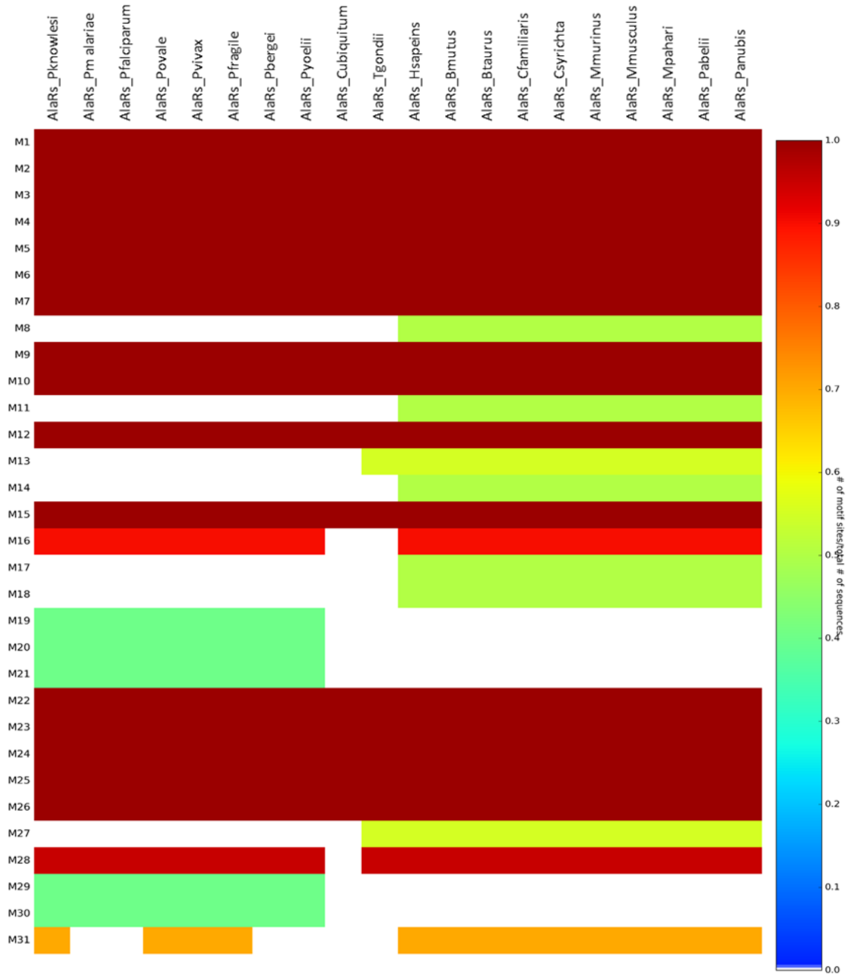
AlaRs_Pknowlesi
AlaRs_Pm alariae
AlaRs_Pfalciparum
AlaRs_Povale
AlaRs_Pvivax
AlaRs_Pfragile
AlaRs_Pbergei
AlaRs_Pyoelii
AlaRs_Cubiquitum
AlaRs_Tgondii
AlaRs_Hsapeins
AlaRs_Bmutus
AlaRs_Btaurus
AlaRs_Cfamiliaris
AlaRs_Csyrichta
AlaRs_Mmurinus
AlaRs_Mmusculus
AlaRs_Mpahari
AlaRs_Pabelii
AlaRs_Panubis
M1
M2
M3
M4
M5
M6
M7
M8
M9
M10
M11
M12
M13
M14
M15
M16
M17
M18
M19
M20
M21
M22
M23
M24
M25
M26
M27
M28
M29
M30
M31
1.0
0.9
0.8
0.7
0.6
0.5
0.4
0.3
0.2
0.1
0.0
# of motif sites/total # of sequences
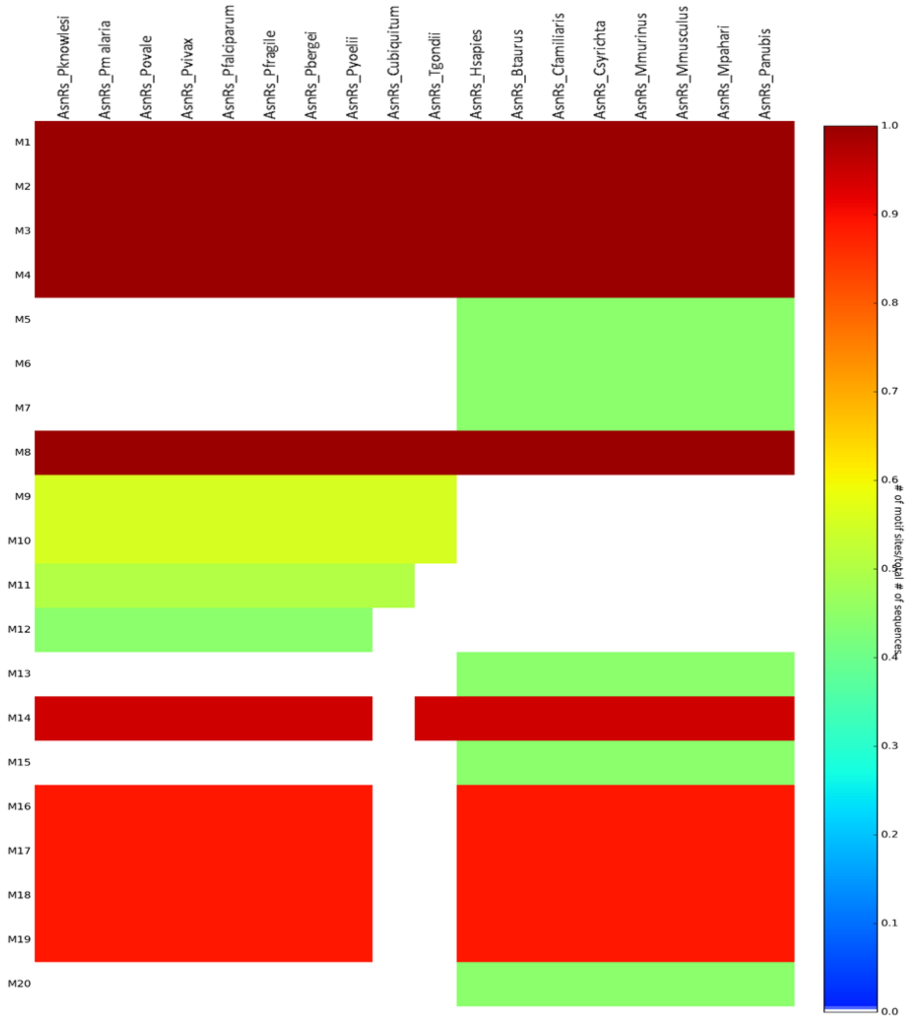
AsnRs_Pknowlesi
AsnRs_Pm alaria
AsnRs_Povale
AsnRs_Pvivax
AsnRs_Pfalciparum
AsnRs_Pfragile
AsnRs_Pbergei
AsnRs_Pyoelii
AsnRs_Cubiquitum
AsnRs_Tgondii
AsnRs_Hsapies
AsnRs_Btaurus
AsnRs_Cfamiliaris
AsnRs_Csyrichta
AsnRs_Mmurinus
AsnRs_Mmusculus
AsnRs_Mpahari
AsnRs_Panubis
M1
M2
M3
M4
M5
M6
M7
M8
M9
M10
M11
M12
M13
M14
M15
M16
M17
M18
M19
M20
1.0
0.9
0.8
0.7
0.6
0.5
0.4
0.3
0.2
0.1
0.0
# of motif sites/total # of sequences

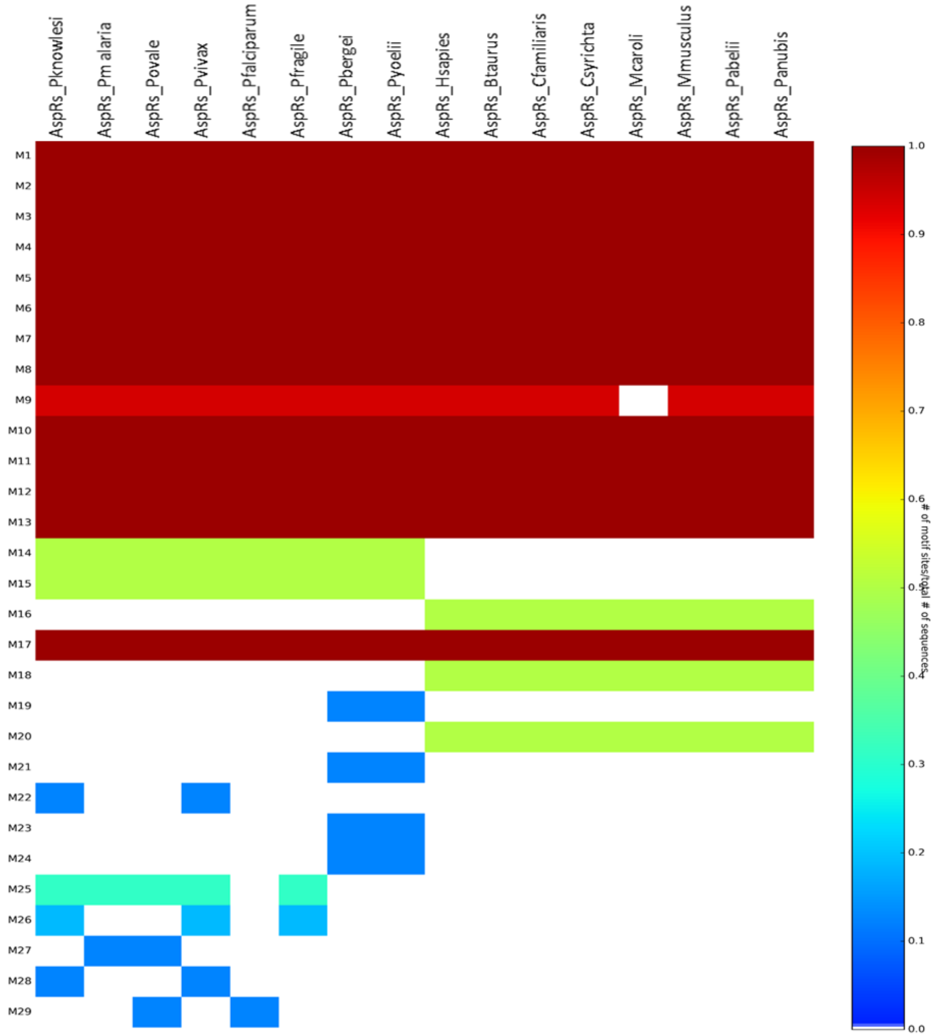
AspRs_Pknowlesi
AspRs_Pm alaria
AspRs_Povale
AspRs_Pvivax
AspRs_Pfalciparum
AspRs_Pfragile
AspRs_Pbergei
AspRs_Pyoelii
AspRs_Hsapies
AspRs_Btaurus
AspRs_Cfamiliaris
AspRs_Csyrichta
AspRs_Mcaroli
AspRs_Mmusculus
AspRs_Pabelii
AspRs_Panubis
M1
M2
M3
M4
M5
M6
M7
M8
M9
M10
M11
M12
M13
M14
M15
M16
M17
M18
M19
M20
M21
M22
M23
M24
M25
M26
M27
M28
M29
1.0
0.9
0.8
0.7
0.6
0.5
0.4
0.3
0.2
0.1
0.0
# of motif sites/total # of sequences
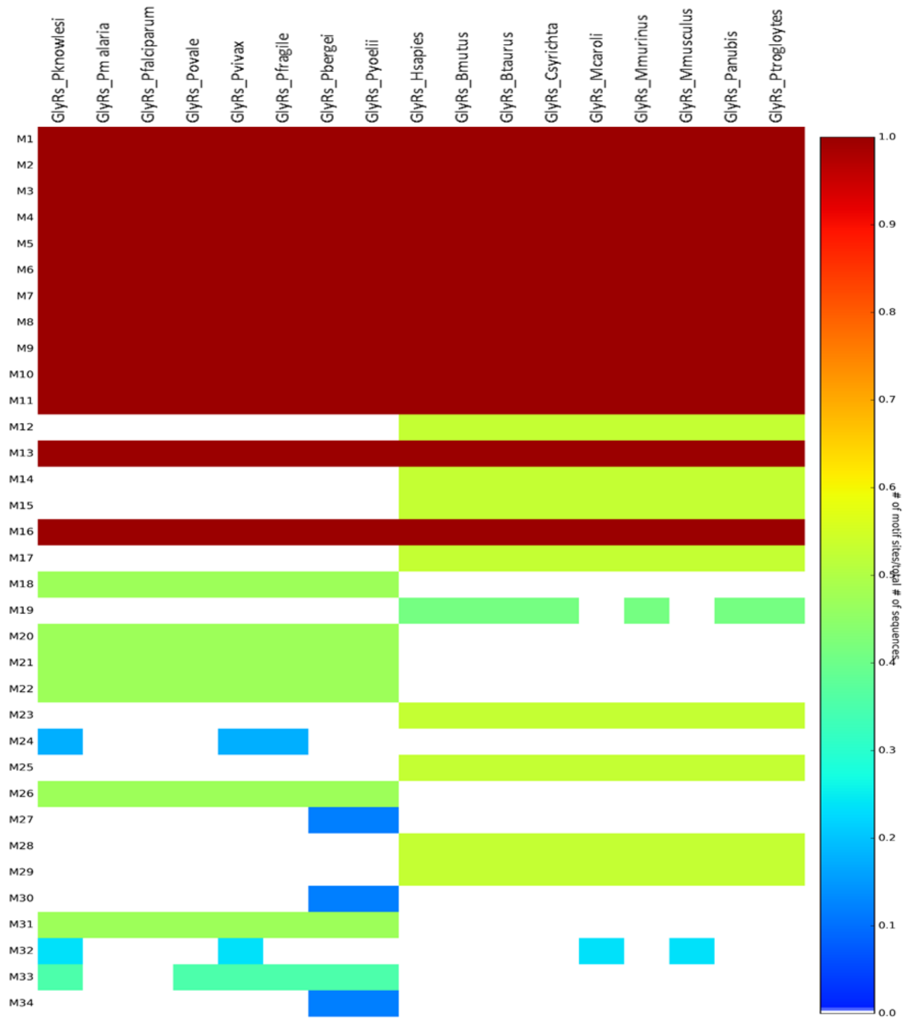
GlyRs_Pknowlesi
GlyRs_Pm alaria
GlyRs_Pfalciparum
GlyRs_Povale
GlyRs_Pvivax
GlyRs_Pfragile
GlyRs_Pbergei
GlyRs_Pyoelii
GlyRs_Hsapies
GlyRs_Bmutus
GlyRs_Btaurus
GlyRs_Csyrichta
GlyRs_Mcaroli
GlyRs_Mmurinus
GlyRs_Mmusculus
GlyRs_Panubis
GlyRs_Ptrogloytes
M1
M2
M3
M4
M5
M6
M7
M8
M9
M10
M11
M12
M13
M14
M15
M16
M17
M18
M19
M20
M21
M22
M23
M24
M25
M26
M27
M28
M29
M30
M31
M32
M33
M34
1.0
0.9
0.8
0.7
0.6
0.5
0.4
0.3
0.2
0.1
0.0
# of motif sites/total # of sequences

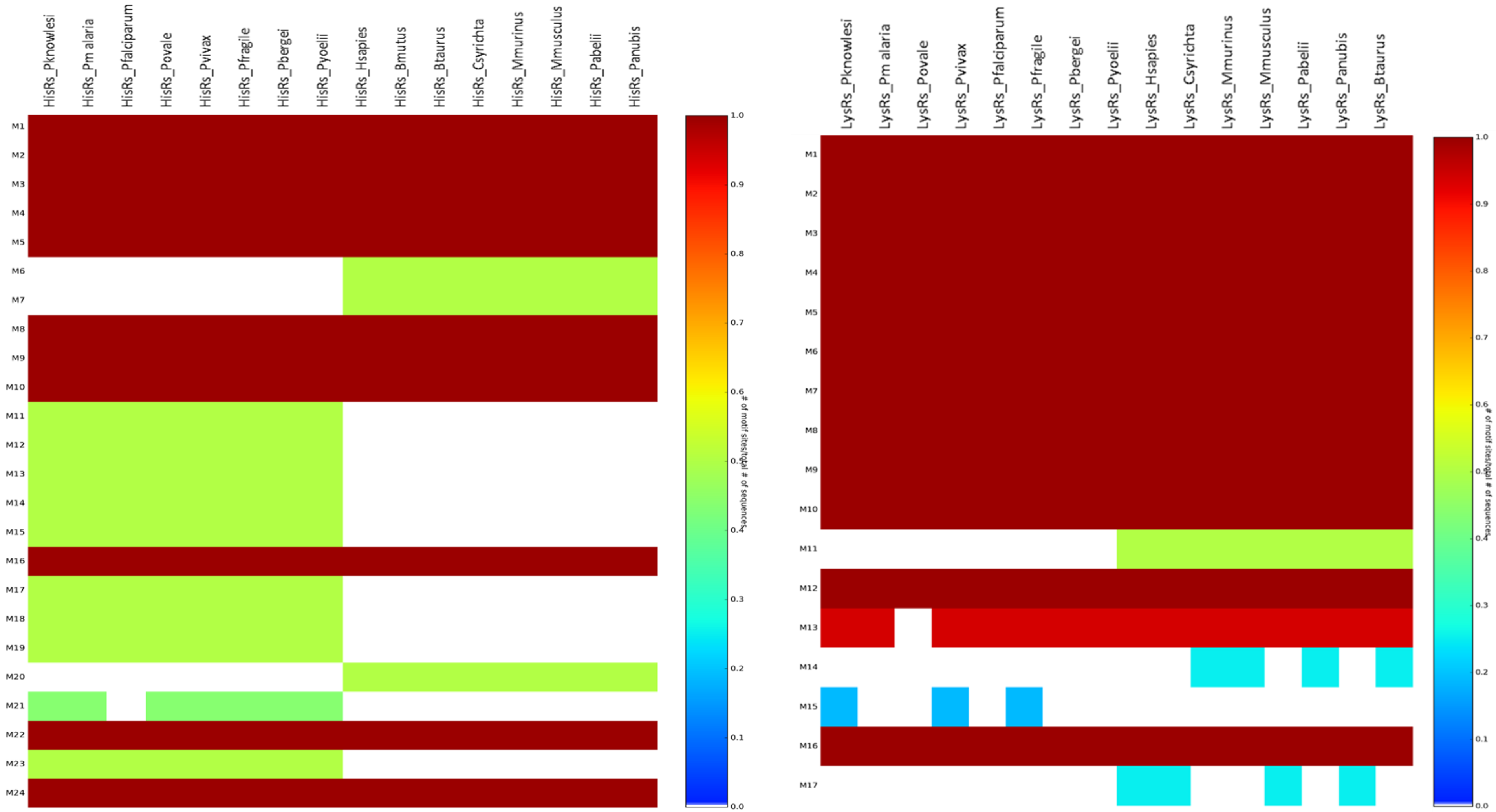
HisRs_Pknowlesi
HisRs_Pm alaria
HisRs_Pfalciparum
HisRs_Povale
HisRs_Pvivax
HisRs_Pfragile
HisRs_Pbergei
HisRs_Pyoelii
HisRs_Hsapies
HisRs_Bmutus
HisRs_Btaurus
HisRs_Csyrichta
HisRs_Mmurinus
HisRs_Mmusculus
HisRs_Pabelii
HisRs_Panubis
M1
M2
M3
M4
M5
M6
M7
M8
M9
M10
M11
M12
M13
M14
M15
M16
M17
M18
M19
M20
M21
M22
M23
M24
LysRs_Pknowlesi
LysRs_Pm alaria
LysRs_Povale
LysRs_Pvivax
LysRs_Pfalciparum
LysRs_Pfragile
LysRs_Pbergei
LysRs_Pyoelii
LysRs_Hsapies
LysRs_Csyrichta
LysRs_Mmurinus
LysRs_Mmusculus
LysRs_Pabelii
LysRs_Panubis
LysRs_Btaurus
# of motif sites/total # of sequences
1.0
0.9
0.8
0.7
0.6
0.4
0.3
0.2
0.1
0.0

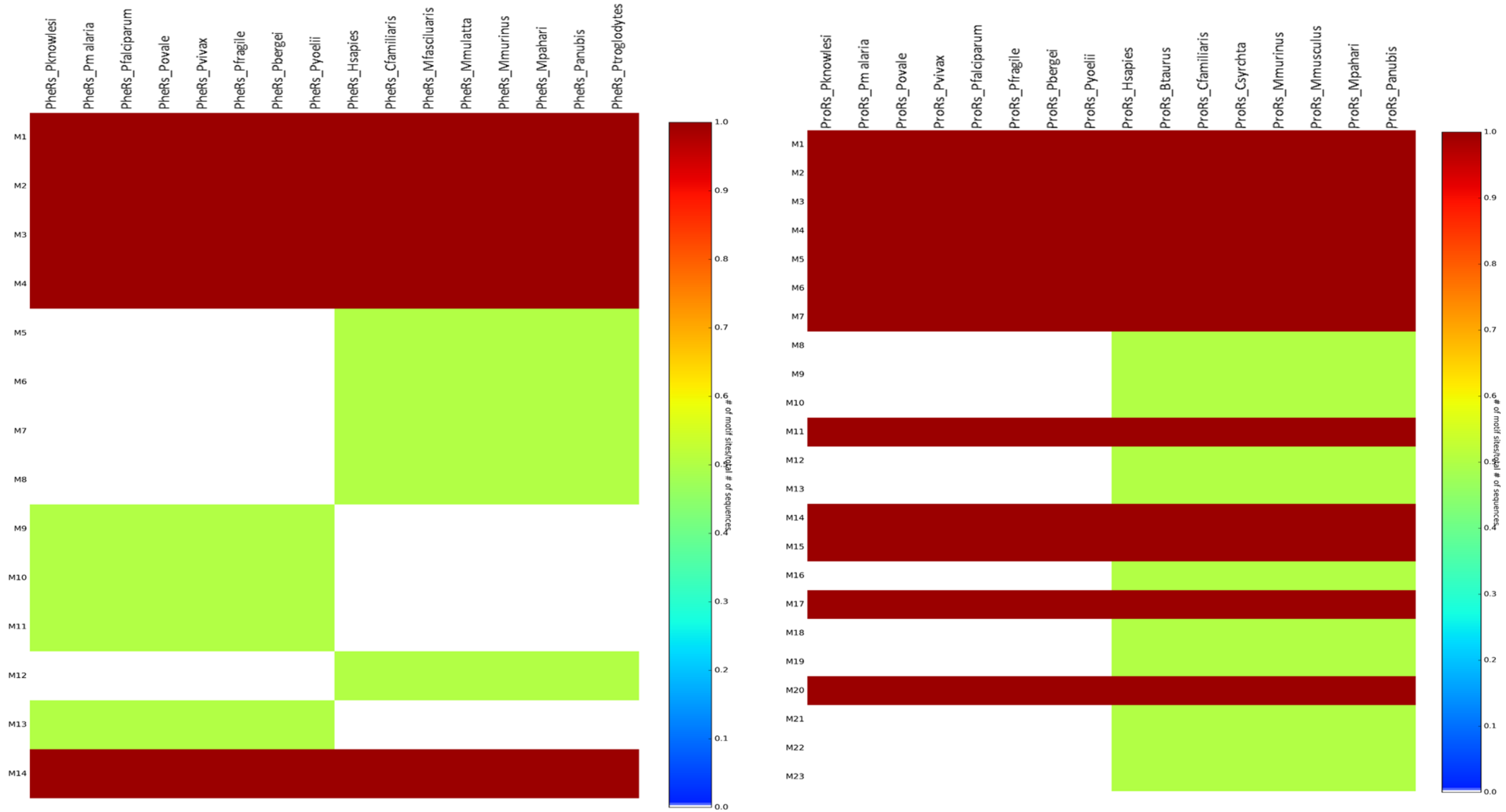

PheRs_Pknowlesi
PheRs_Pm alaria
PheRs_Pfalciparum
PheRs_Povale
PheRs_Pvivax
PheRs_Pfragile
PheRs_Pbergei
PheRs_Pyoelii
PheRs_Hsapies
PheRs_Cfamiliaris
PheRs_Mfasciluaris
PheRs_Mmulatta
PheRs_Mmurinus
PheRs_Mpahari
PheRs_Panubis
PheRs_Ptroglodytes
M1
M2
M3
M4
M5
M6
M7
M8
M9
M10
M11
M12
M13
M14
1.0
0.9
0.8
0.7
0.6
0.4
0.3
0.2
0.1
0.0
# of motif sites/total # of sequences
ProRs_Pknowlesi
ProRs_Pm alaria
ProRs_Povale
ProRs_Pvivax
ProRs_Pfalciparum
ProRs_Pfragile
ProRs_Pbergei
ProRs_Pyoelii
ProRs_Hsapies
ProRs_Btaurus
ProRs_Cfamiliaris
ProRs_Csyrchta
ProRs_Mmurinus
ProRs_Mmusculus
ProRs_Mpahari
ProRs_Panubis
M1
M2
M3
M4
M5
M6
M7
M8
M9
M10
M11
M12
M13
M14
M15
M16
M17
M18
M19
M20
M21
M22
M23
1.0
0.9
0.8
0.7
0.6
0.4
0.3
0.2
0.1
0.0
# of motif sites/total # of sequences

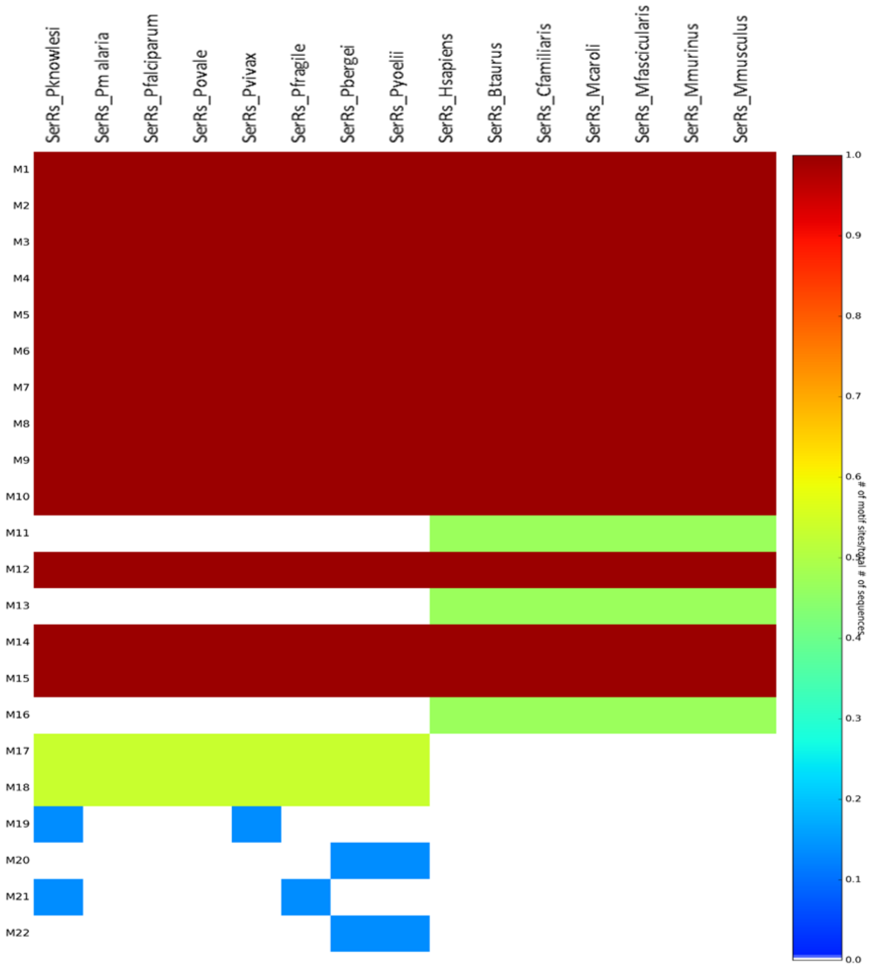
SerRs_Pknowlesi
SerRs_Pm alaria
SerRs_Pfalciparum
SerRs_Povale
SerRs_Pvivax
SerRs_Pfragile
SerRs_Pbergei
SerRs_Pyoelii
SerRs_Hsapiens
SerRs_Btaurus
SerRs_Cfamiliaris
SerRs_Mcaroli
SerRs_Mfascicularis
SerRs_Mmurinus
SerRs_Mmusculus
M1
M2
M3
M4
M5
M6
M7
M8
M9
M10
M11
M12
M13
M14
M15
M16
M17
M18
M19
M20
M21
M22
1.0
0.9
0.8
0.7
0.6
0.5
0.4
0.3
0.2
0.1
0.0
# of motif sites/total # of sequences

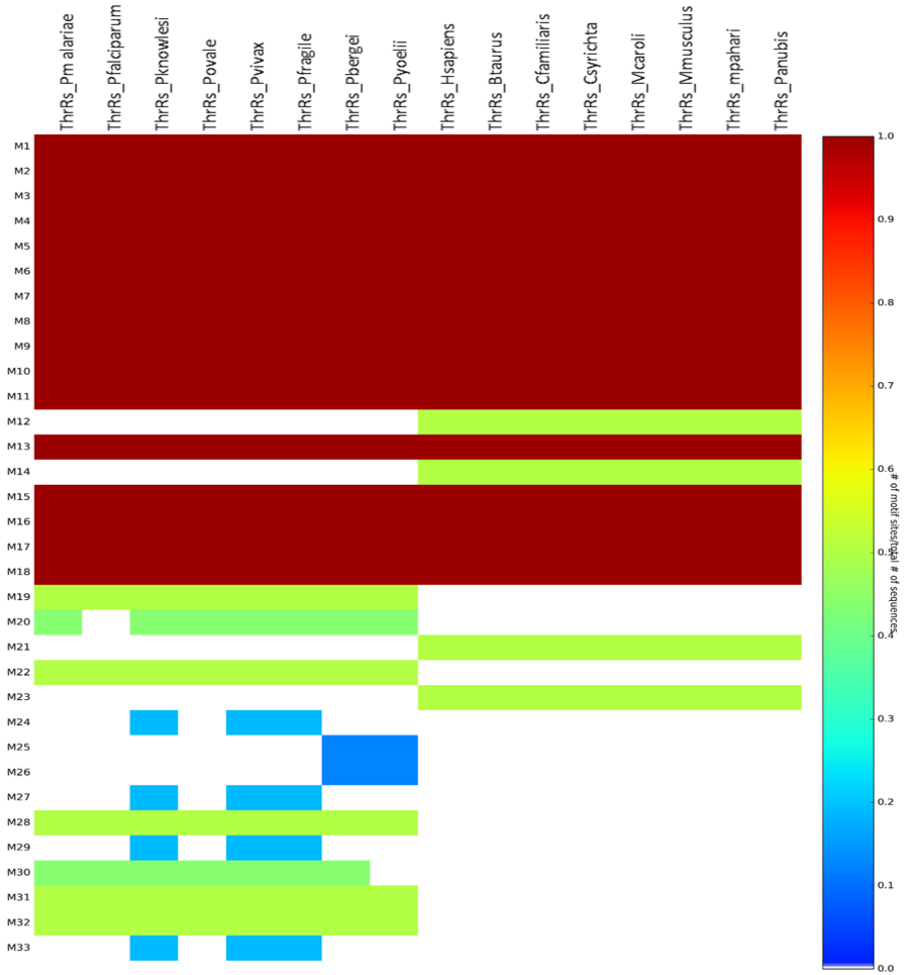
ThrRs_Pm alariae
ThrRs_Pfalciparum
ThrRs_Pknowlesi
ThrRs_Povale
ThrRs_Pvivax
ThrRs_Pfragile
ThrRs_Pbergei
ThrRs_Pyoelii
ThrRs_Hsapiens
ThrRs_Btaurus
ThrRs_Cfamiliaris
ThrRs_Csyrichta
ThrRs_Mcaroli
ThrRs_Mmusculus
ThrRs_mpahari
ThrRs_Panubis
M1
M2
M3
M4
M5
M6
M7
M8
M9
M10
M11
M12
M13
M14
M15
M16
M17
M18
M19
M20
M21
M22
M23
M24
M25
M26
M27
M28
M29
M30
M31
M32
M33
1.0
0.9
0.8
0.7
0.6
0.5
0.4
0.3
0.2
0.1
0.0
# of motif sites/total # of sequences
