# Supplementary material for: Aminoacyl tRNA synthetases as malarial drug targets: a comparative bioinformatics study
Source: Malar J. 2019 Feb 6;18:34. doi: 10.1186/s12936-019-2665-6 (PMC6366043; doi:10.1186/s12936-019-2665-6)
Supplement: Supplementary file 4 — Additional file 4. Results on mapping of discovered motifs on multiple sequence alignments for the 20 aaRS families. Multiple sequence alignment was performed using TCOFFEE software with default parameters. [file 12936_2019_2665_MOESM4_ESM.pdf]

Additional file 4: Results on mapping of discovered motifs on alignments for the 20 aaRS families. Multiple sequence alignment was performed using TCOFFEE software with default parameters. A purple colour shows motifs conserved in all sequences, blue colour shows motifs only conserved in mammalian species and green shows motifs that are conserved in only the plasmodium species. Motif numbering is based on MEME results.

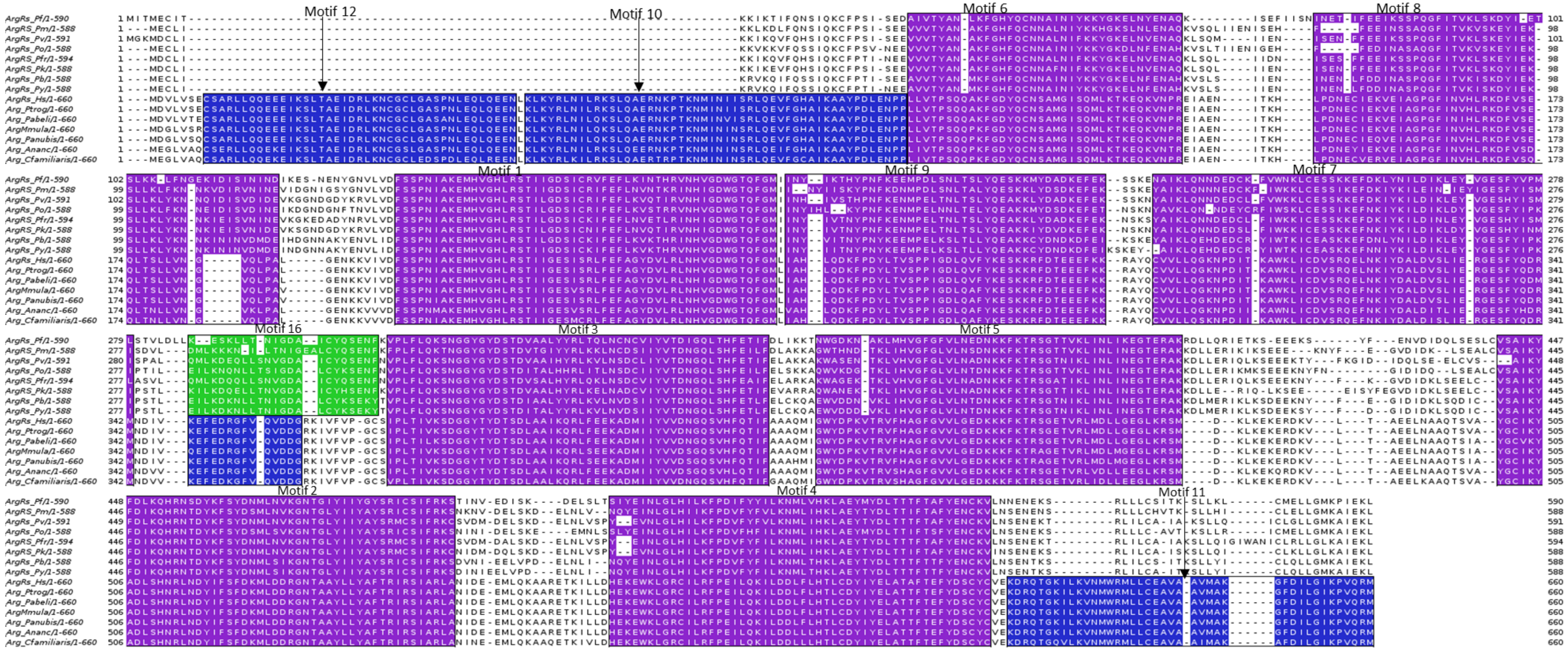

Additional file 4A: Mapping of motifs discovered in ArgRS family to the multiple sequence alignment

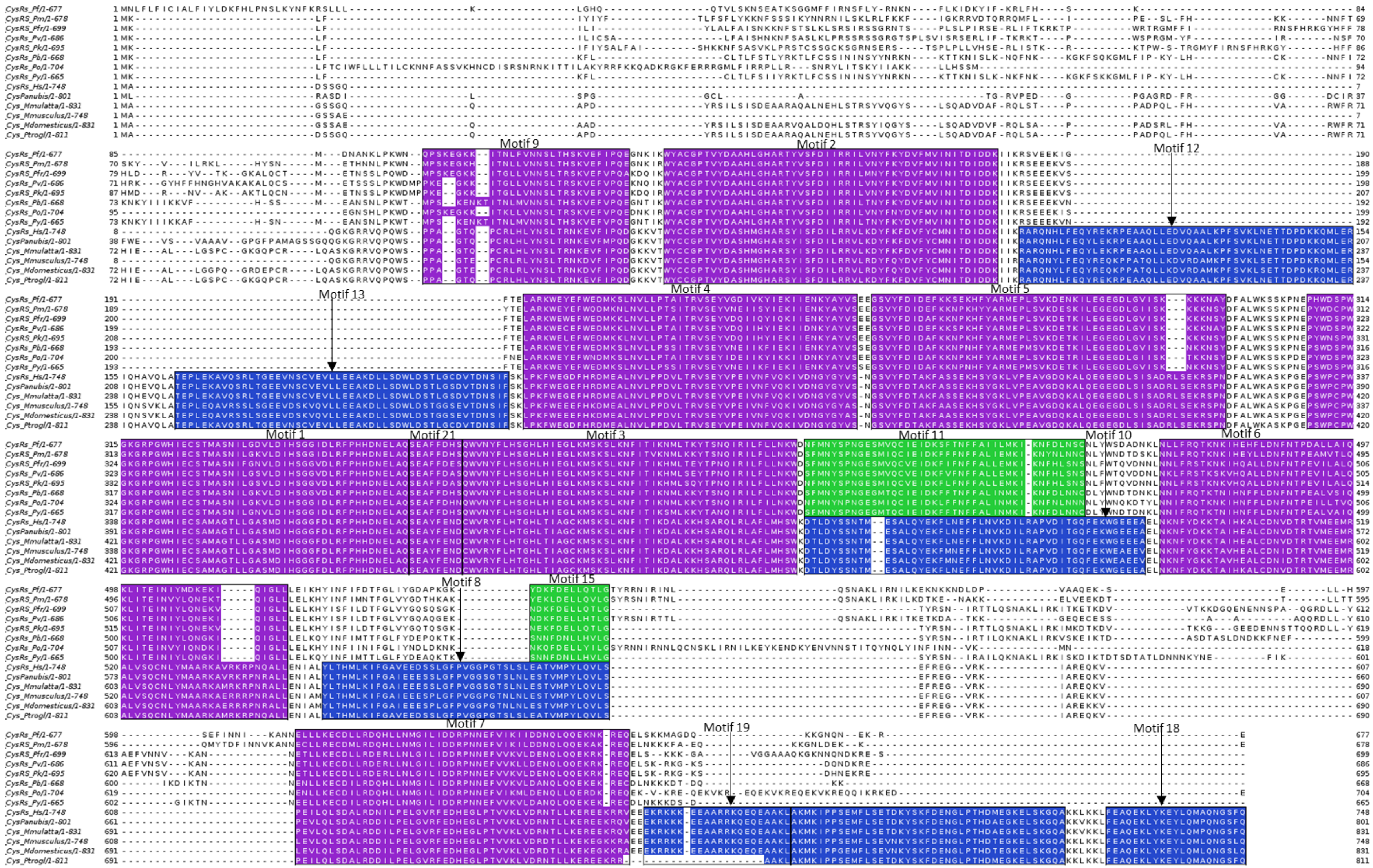

Additional file 4B: Mapping of motifs discovered in CysRS family to the multiple sequence alignment

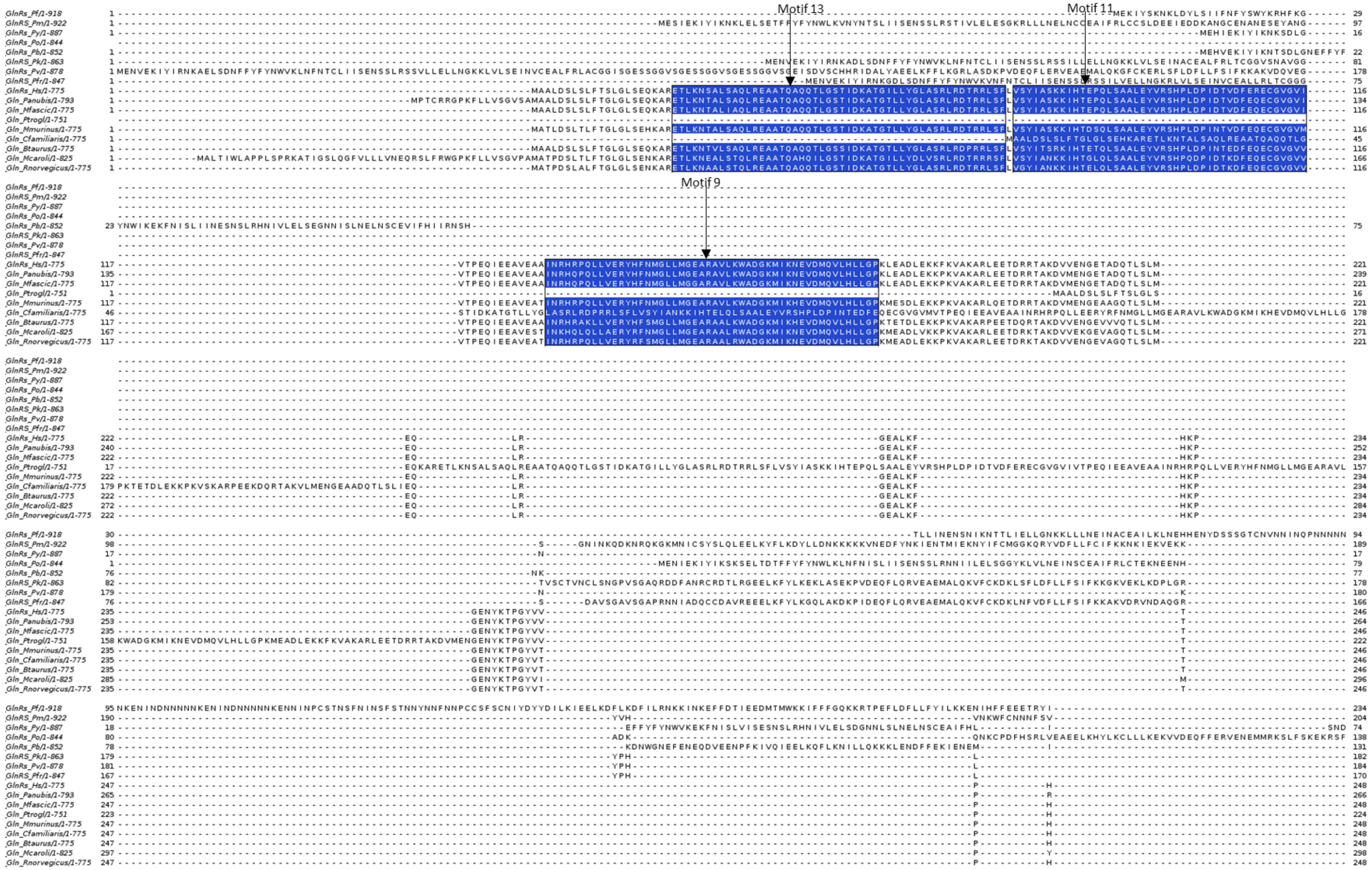

Additional file 4C: Mapping of motifs discovered in GlnRS family to the multiple sequence alignment

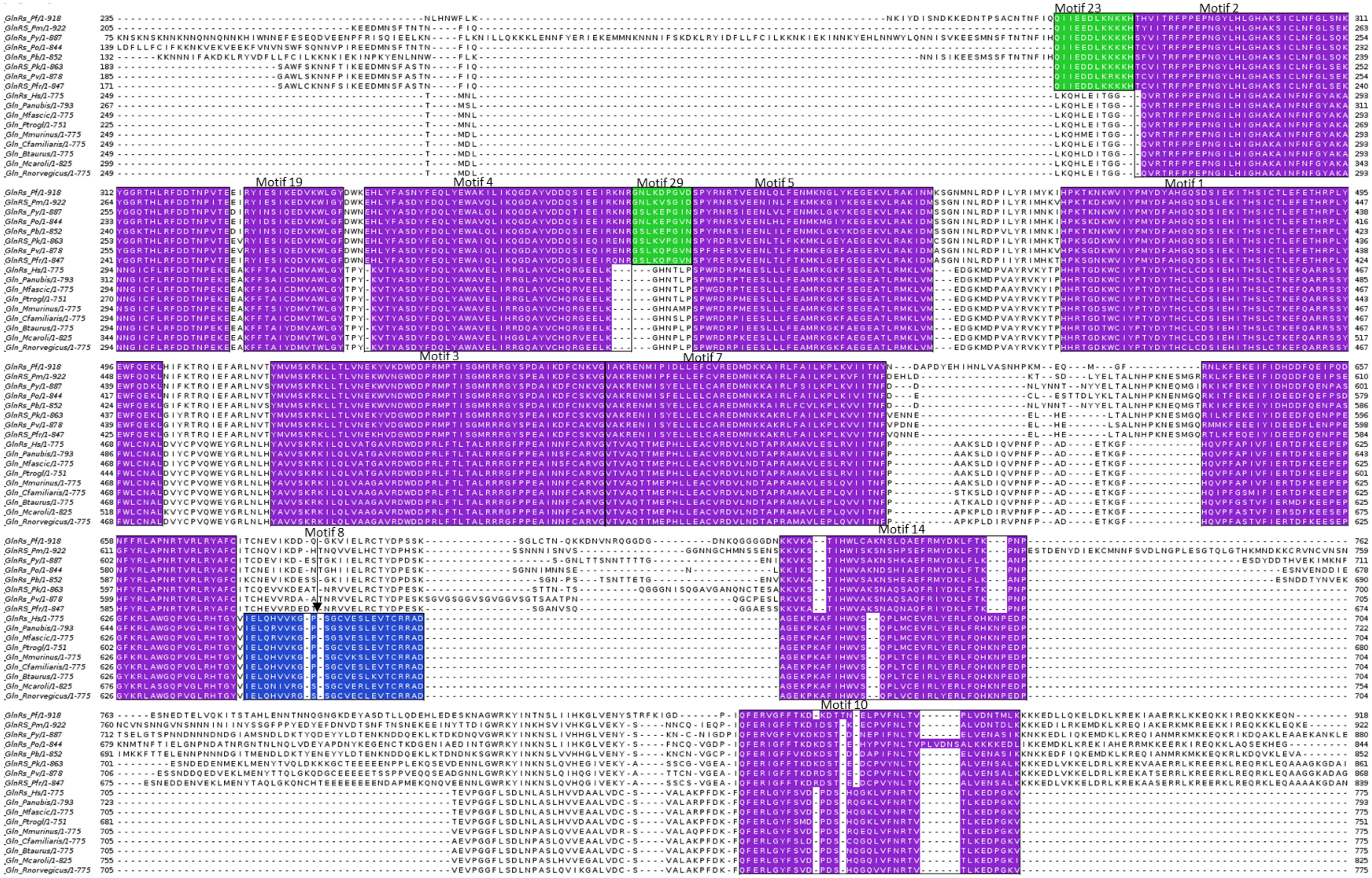

Additional file 4C: Mapping of motifs discovered in GlnRS family to the multiple sequence alignment

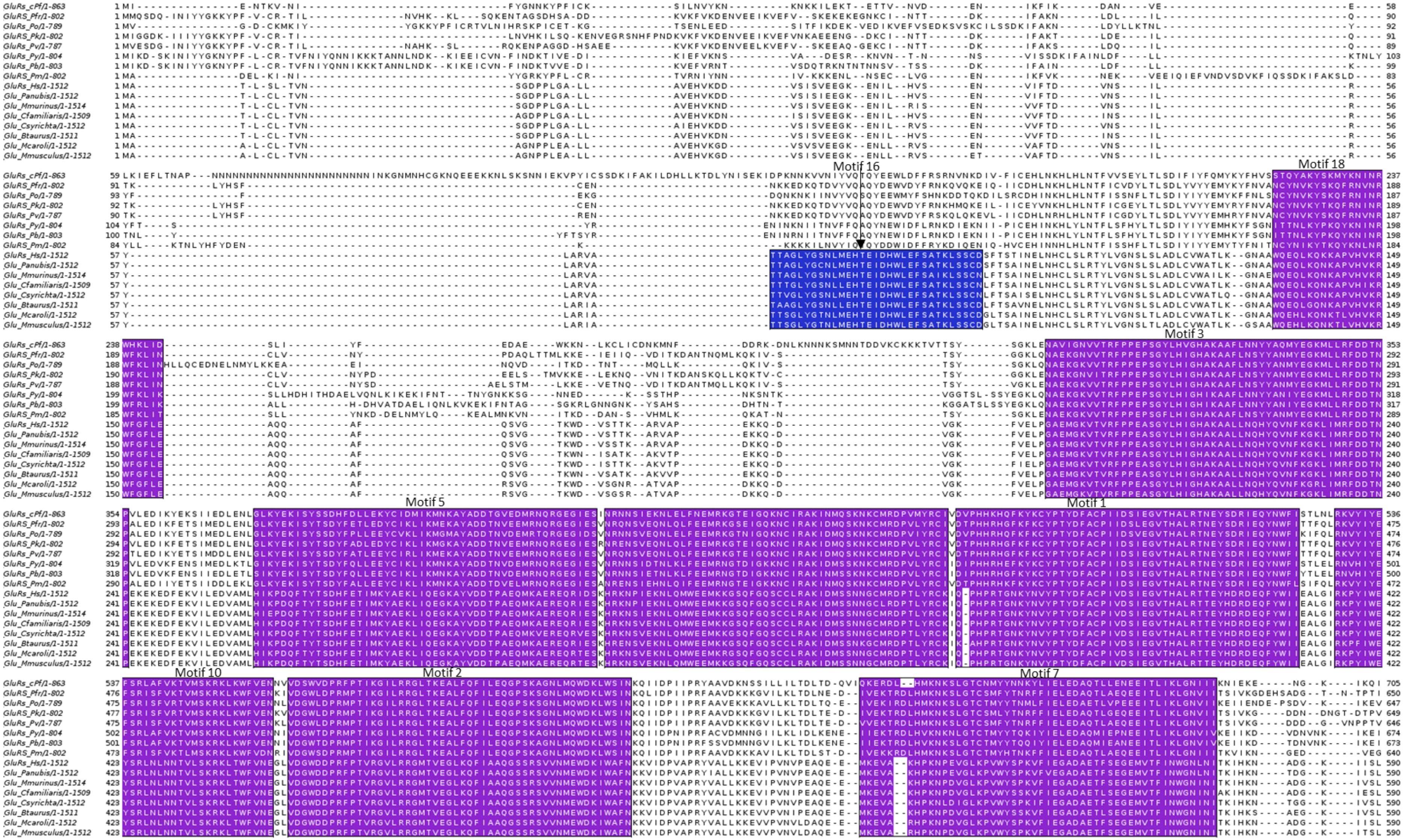

Additional file 4D: Mapping of motifs discovered in GluRS family to the multiple sequence alignment

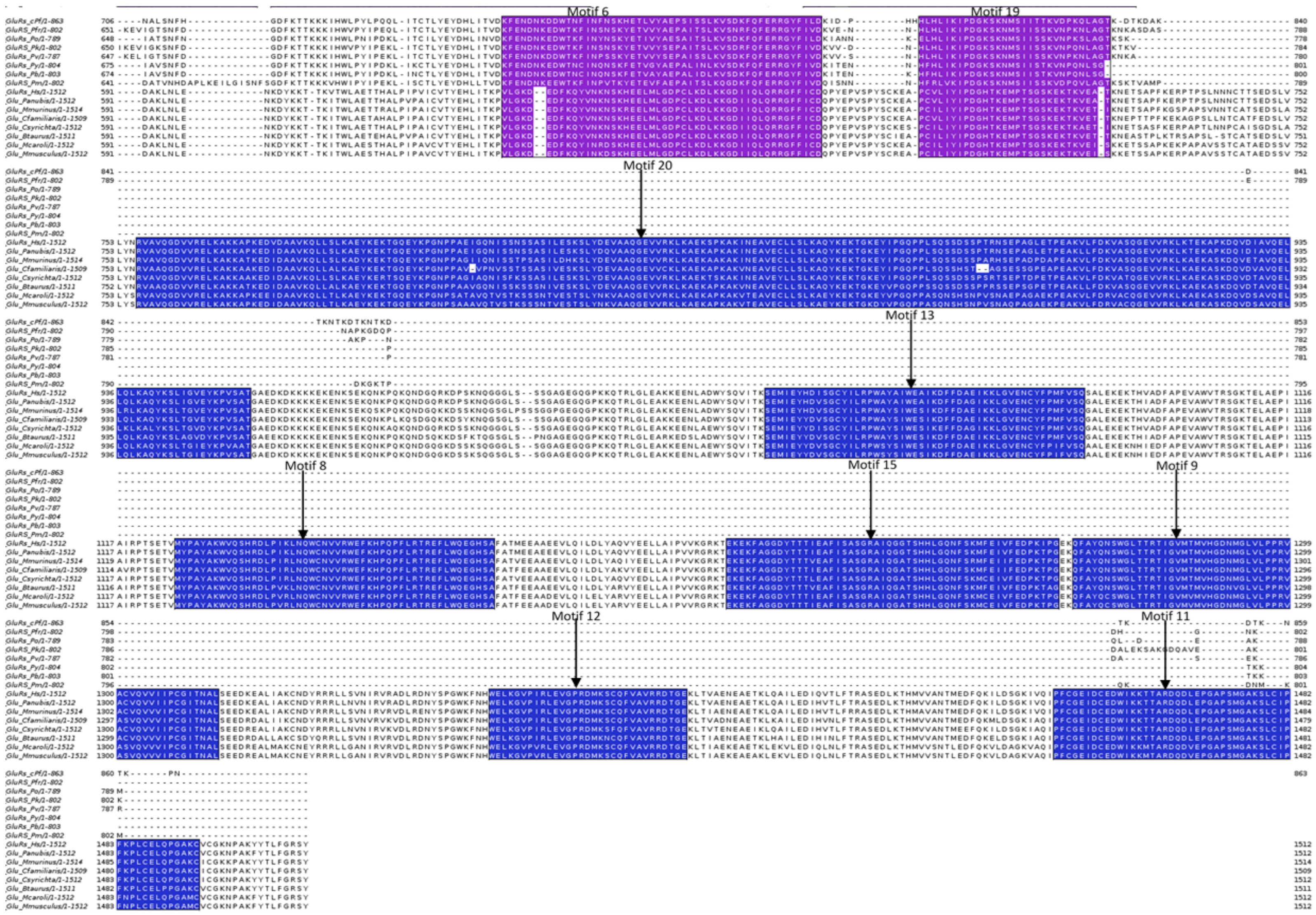

Additional file 4D: Mapping of motifs discovered in GluRS family to the multiple sequence alignment

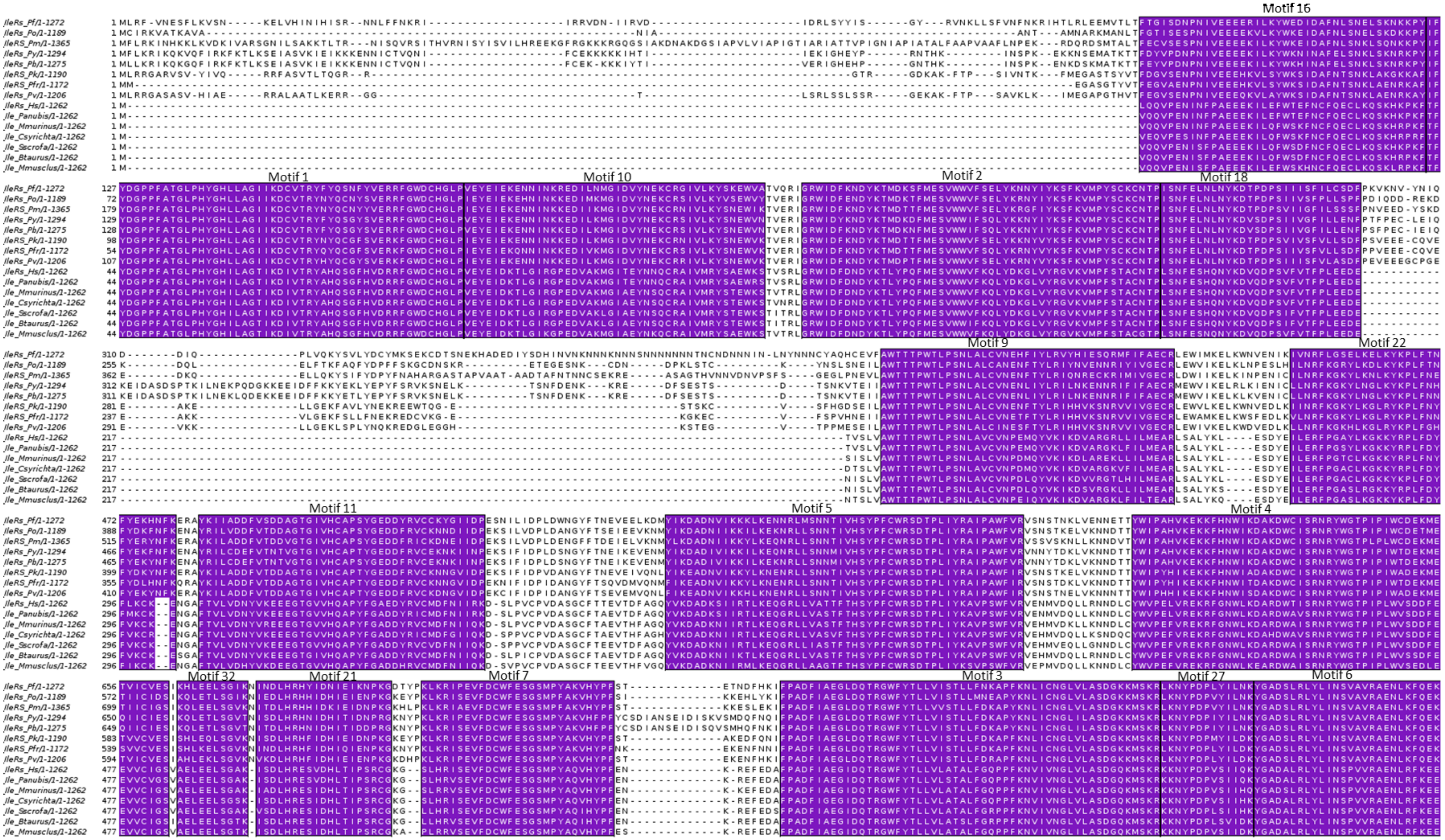

Additional file 4E: Mapping of motifs discovered in IleRS family to the multiple sequence alignment

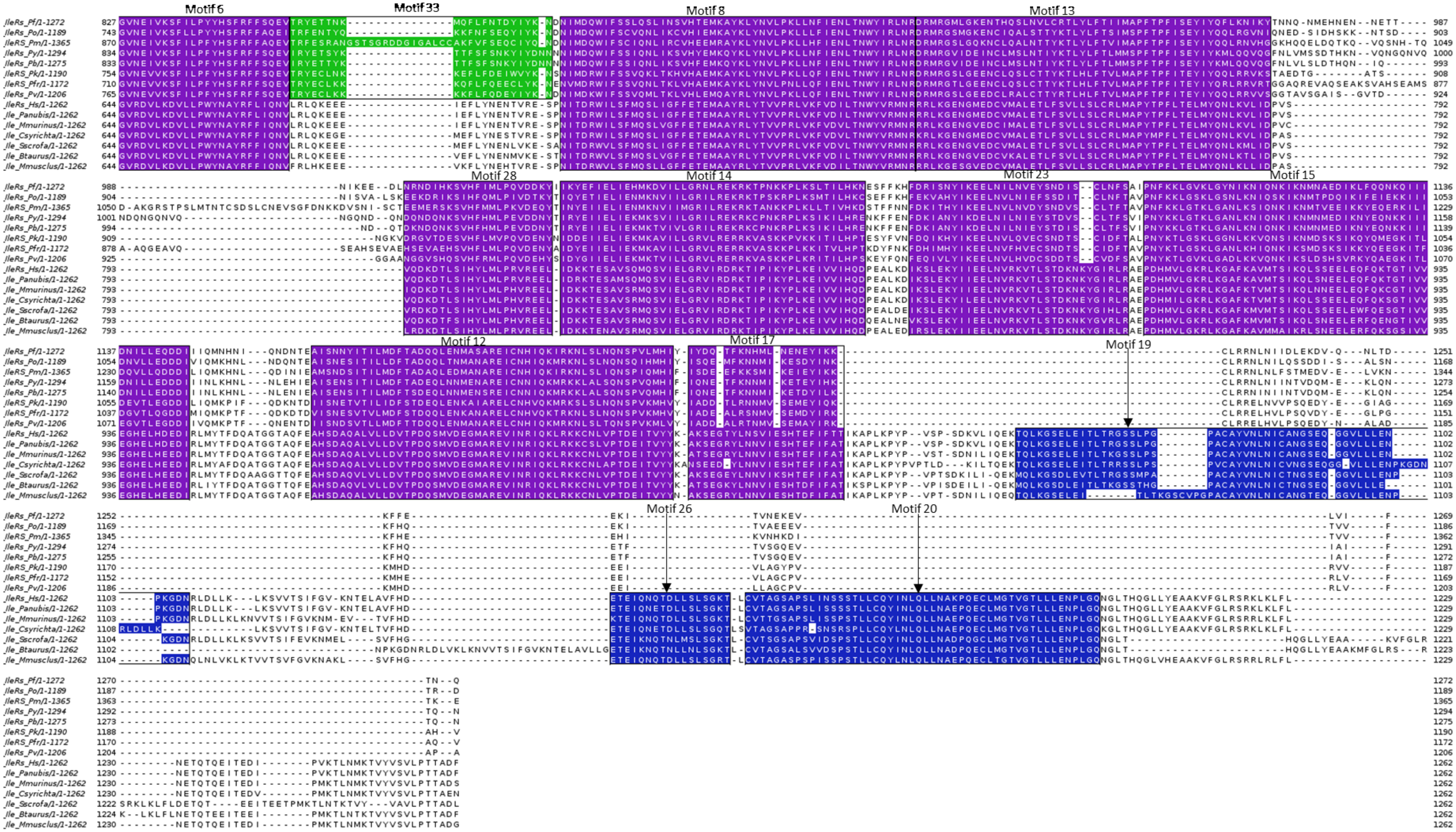

Additional file 4E: Mapping of motifs discovered in IleRS family to the multiple sequence alignment

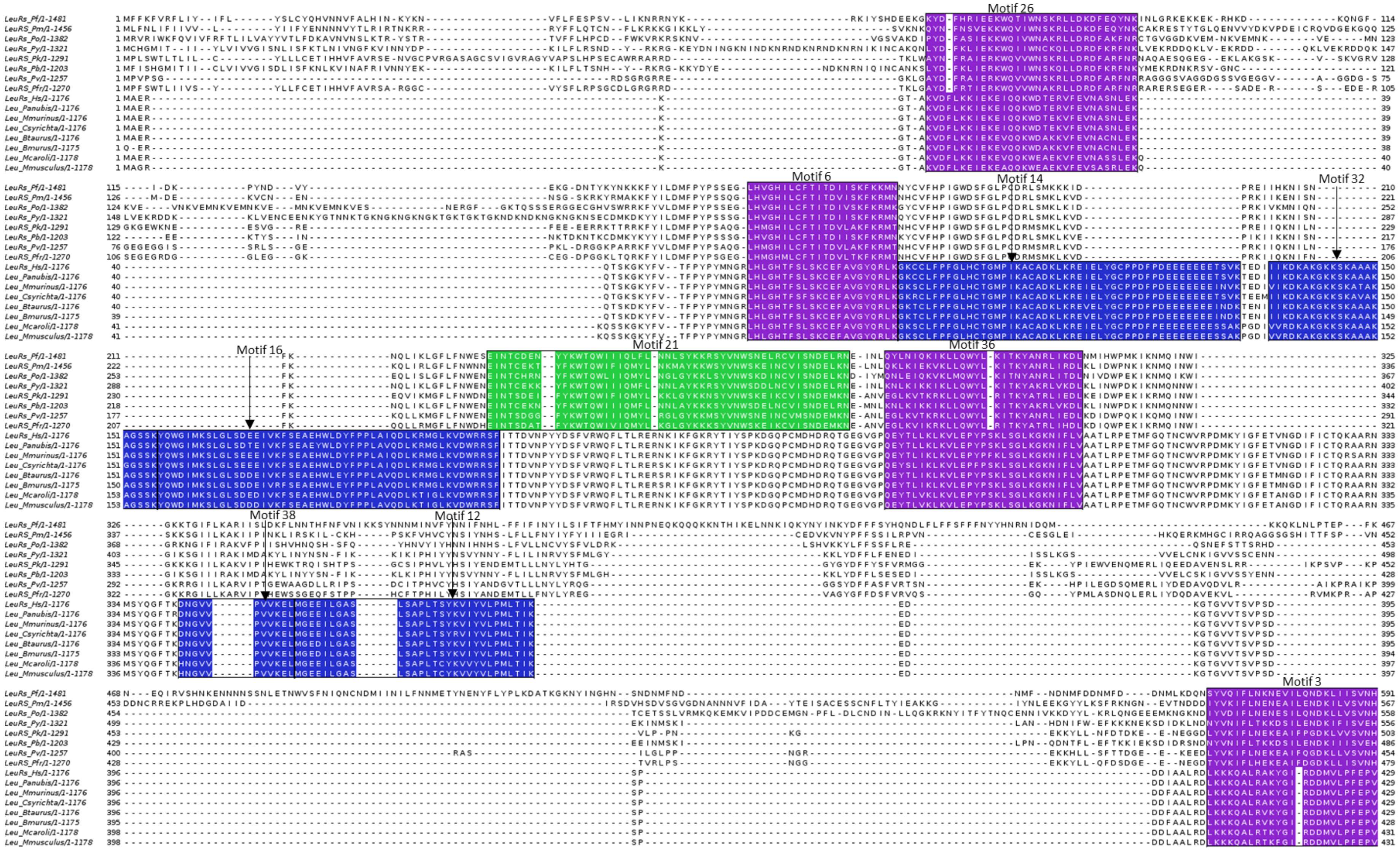

Additional file 4F: Mapping of motifs discovered in LeuRS family to the multiple sequence alignment

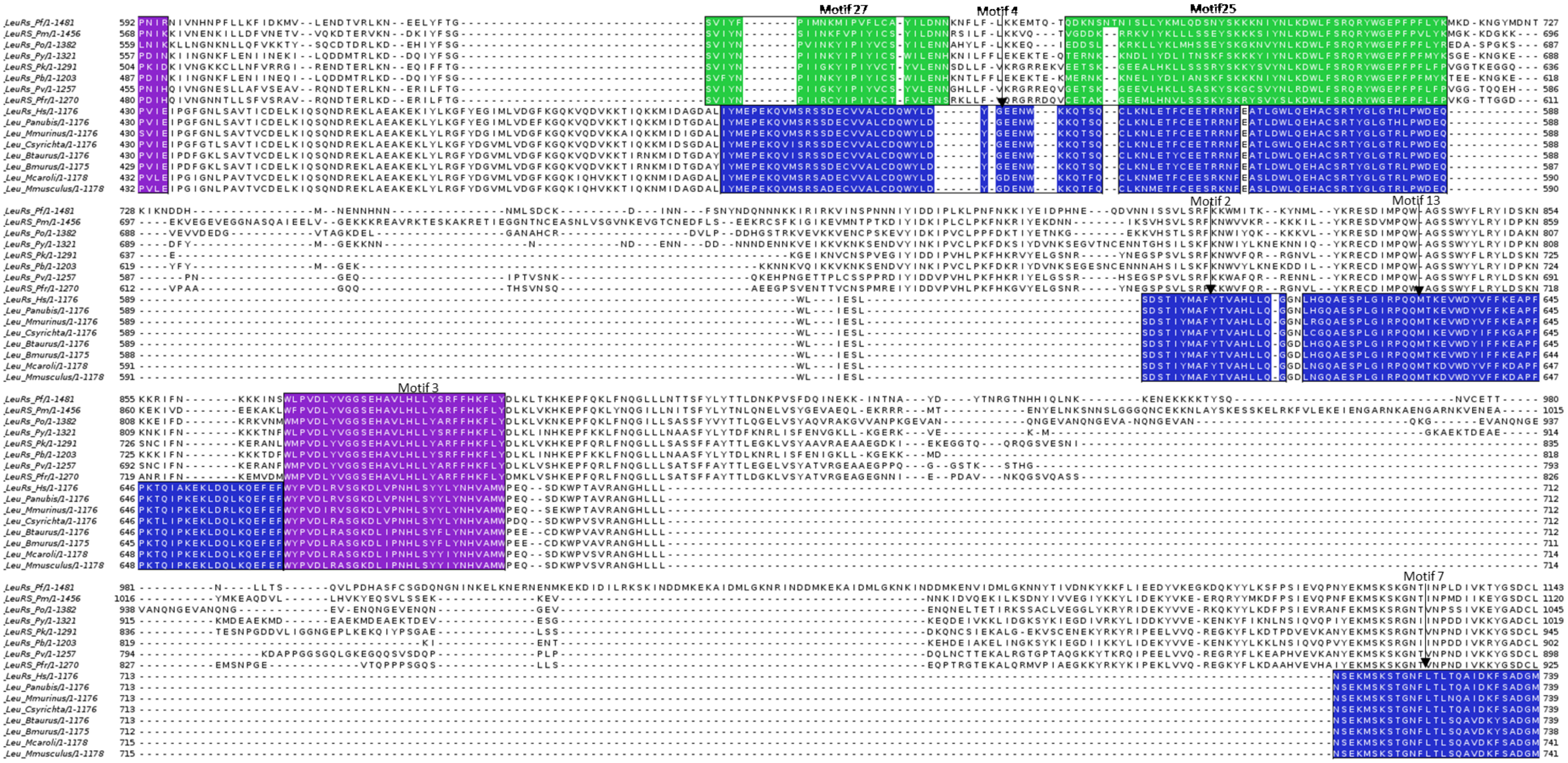

Additional file 4F: Mapping of motifs discovered in LeuRS family to the multiple sequence alignment

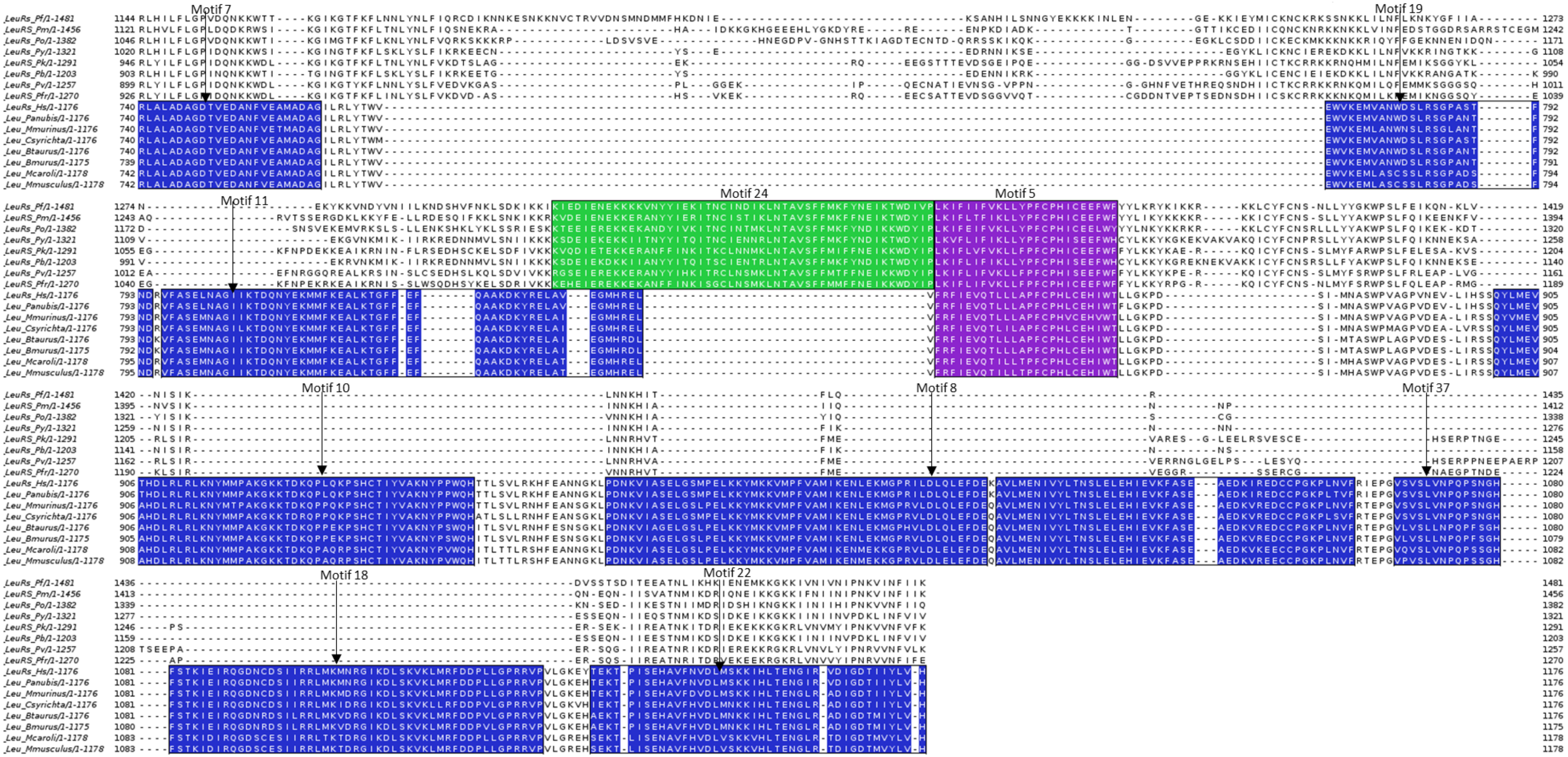

Additional file 4F: Mapping of motifs discovered in LeuRS family to the multiple sequence alignment

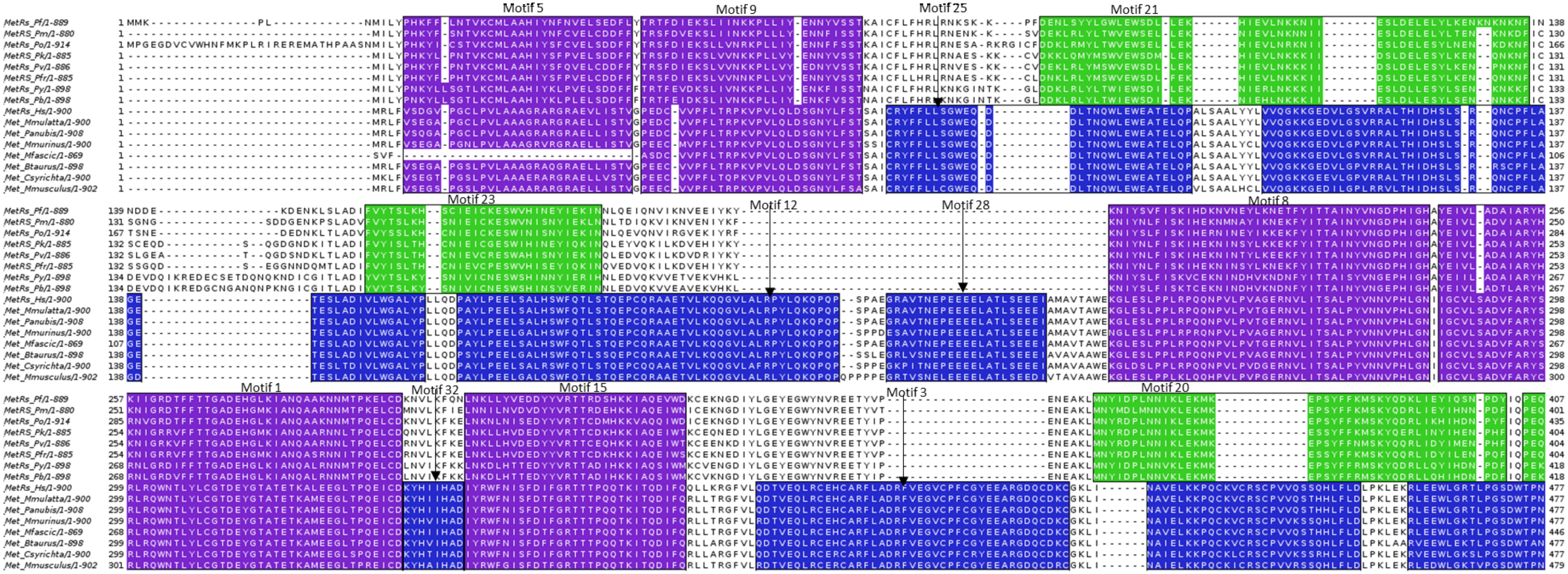

Additional file 4G: Mapping of motifs discovered in MetRS family to the multiple sequence alignment

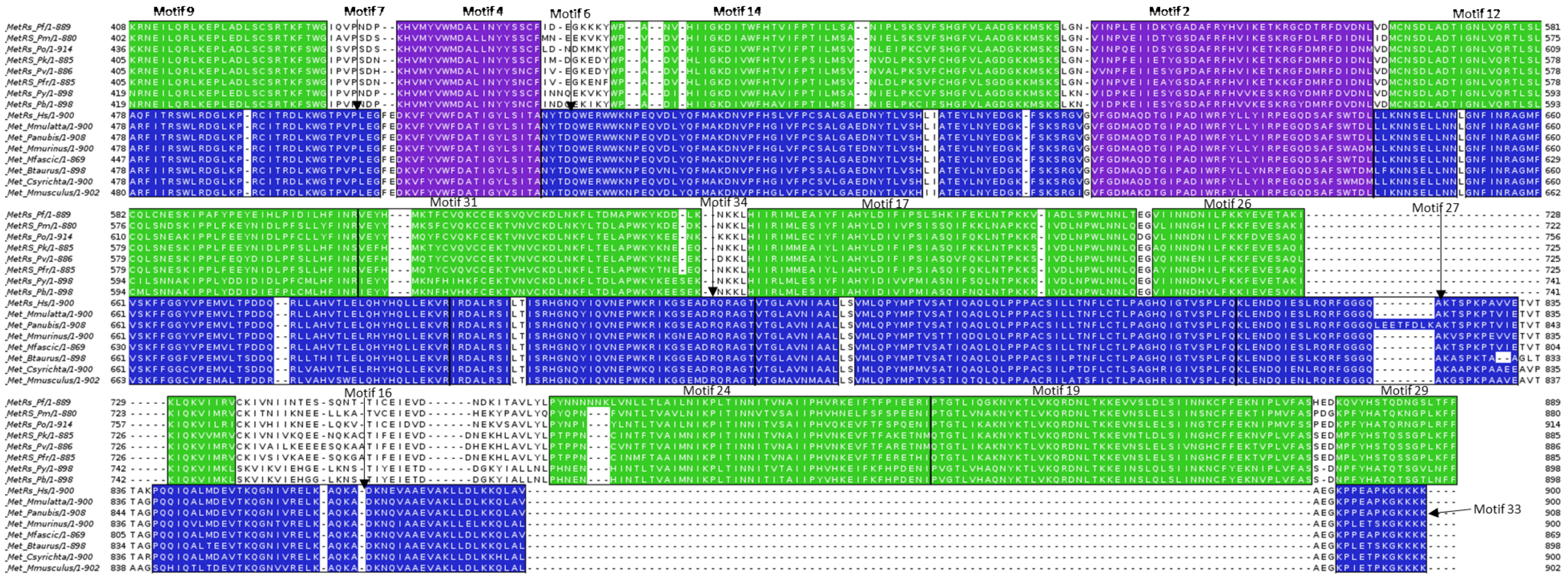

Additional file 4G: Mapping of motifs discovered in MetRS family to the multiple sequence alignment

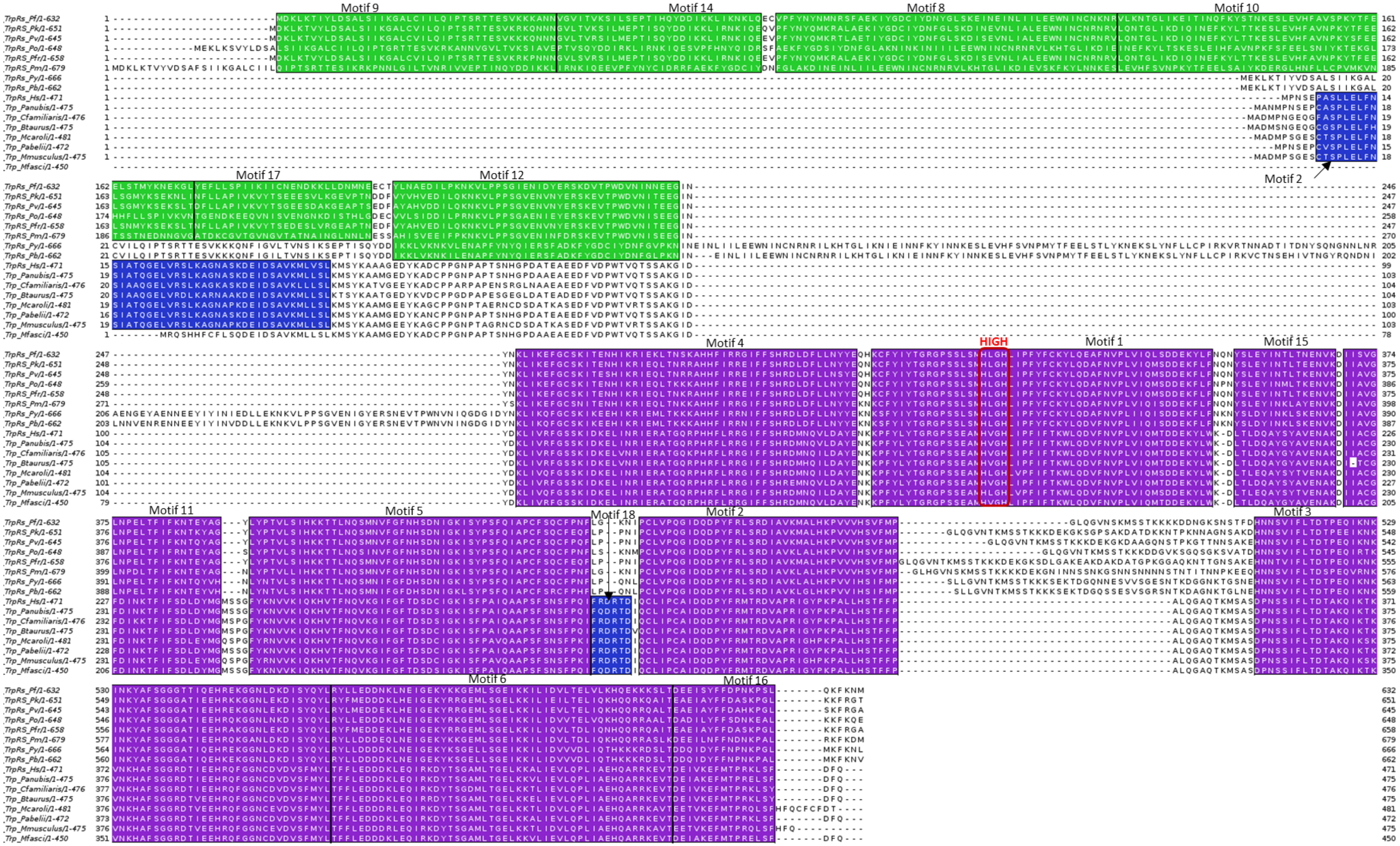

Additional file 4H: Mapping of motifs discovered in TrpRS family to the multiple sequence alignment

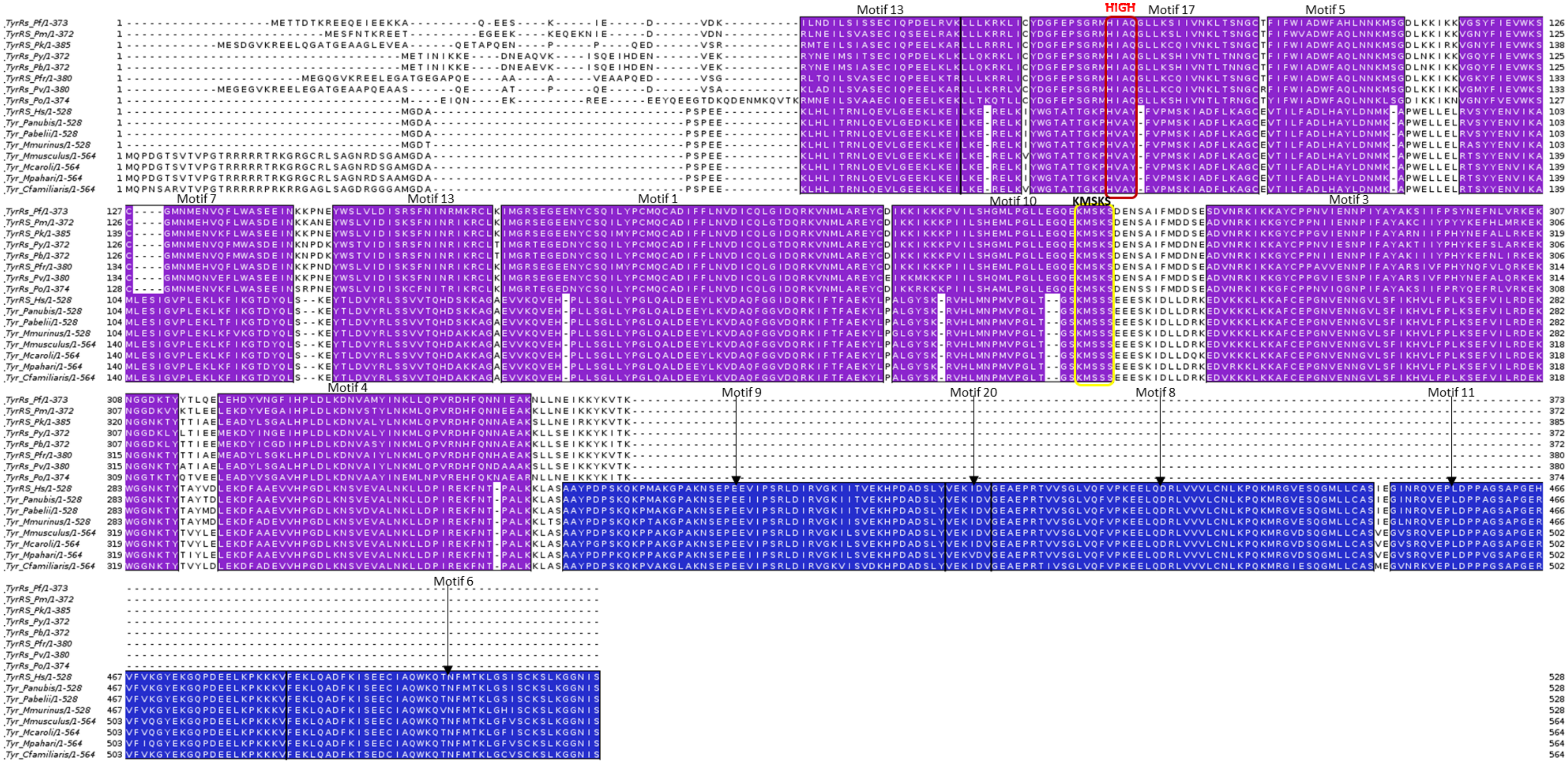

Additional file 4I: Mapping of motifs discovered in TyrRS family to the multiple sequence alignment

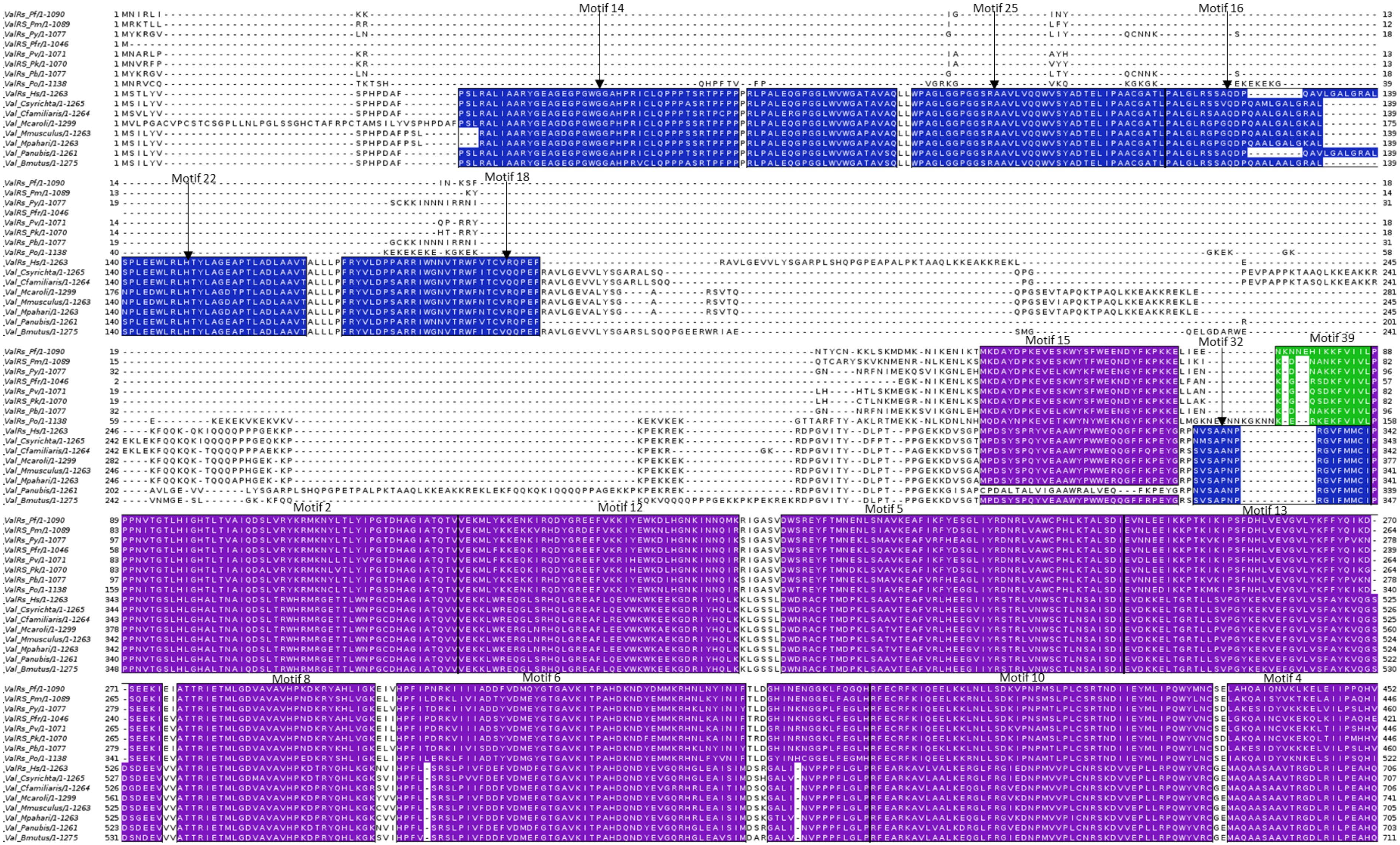

Additional file 4J: Mapping of motifs discovered in ValRS family to the multiple sequence alignment

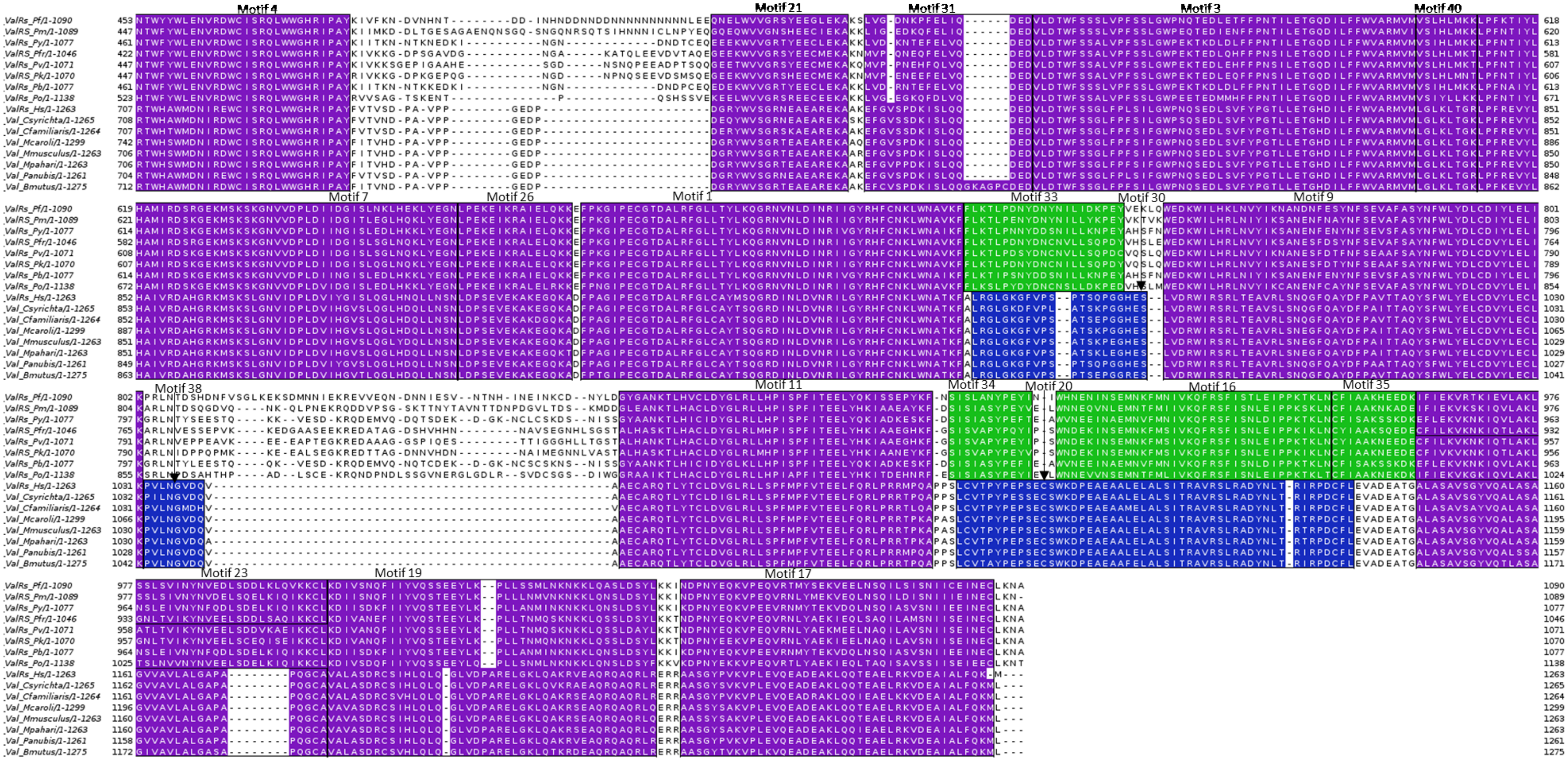

Additional file 4J: Mapping of motifs discovered in ValRS family to the multiple sequence alignment

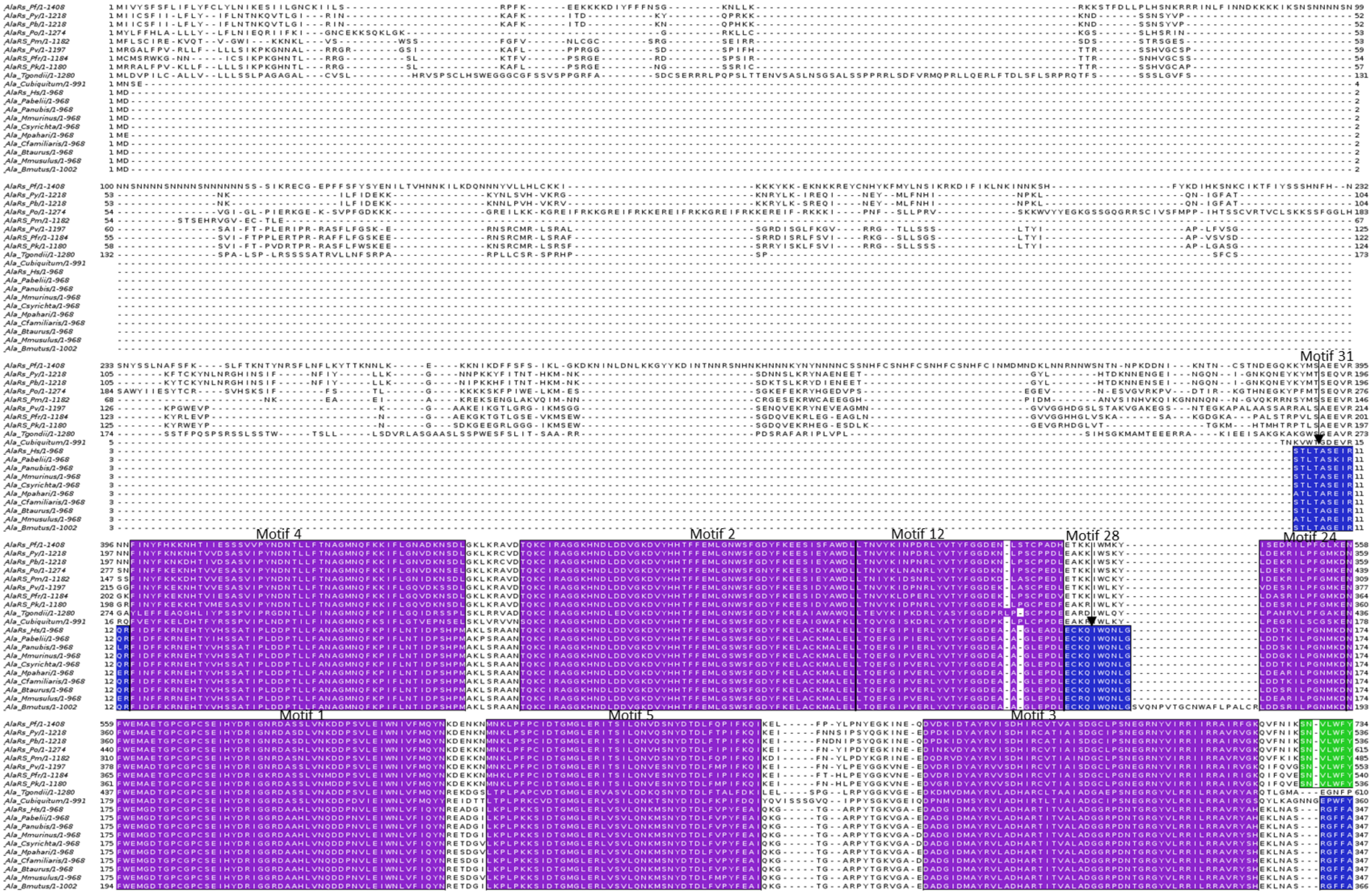

Additional file 4K: Mapping of motifs discovered in AlaRS family to the multiple sequence alignment

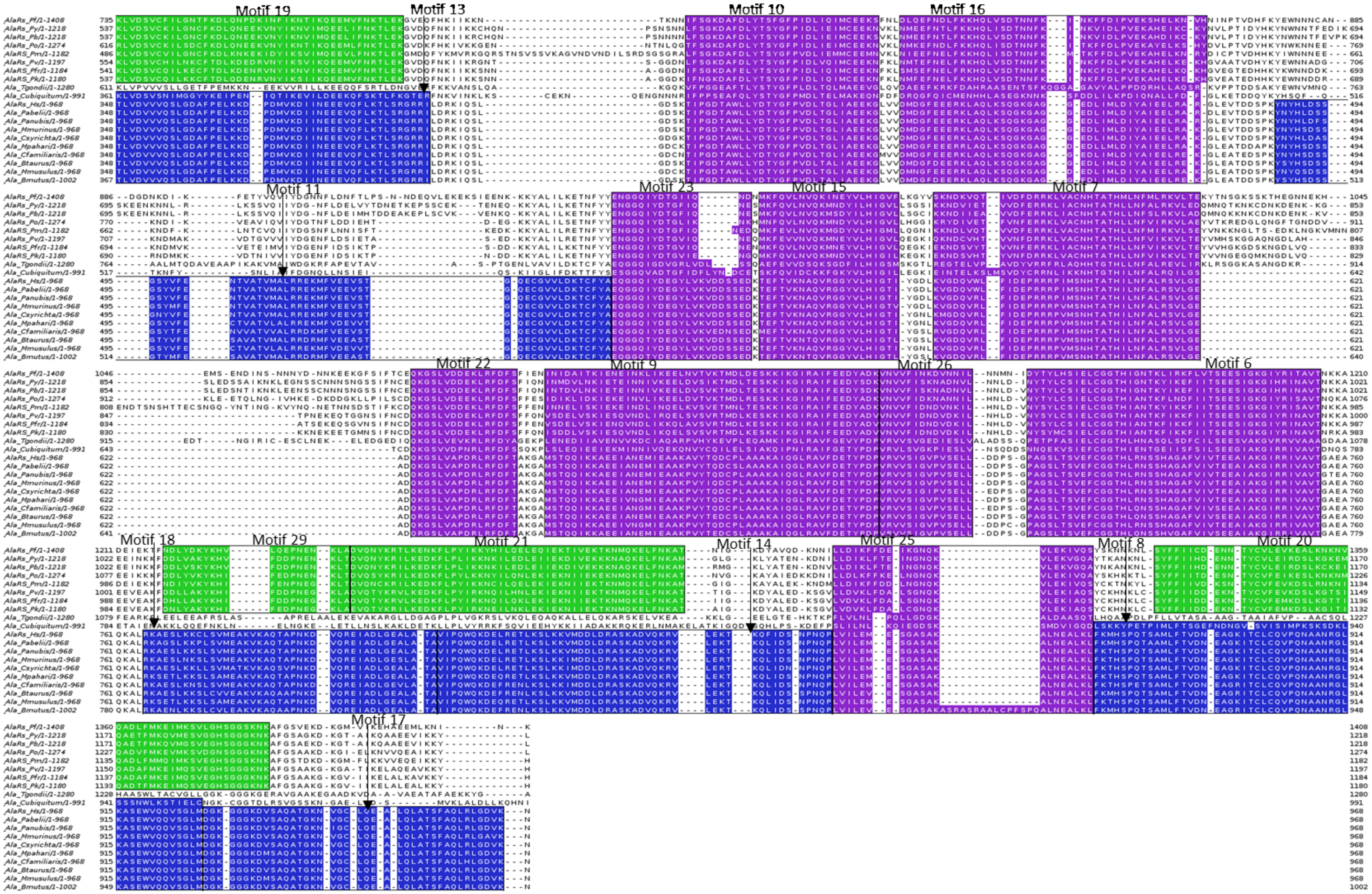

Additional file 4K: Mapping of motifs discovered in AlaRS family to the multiple sequence alignment

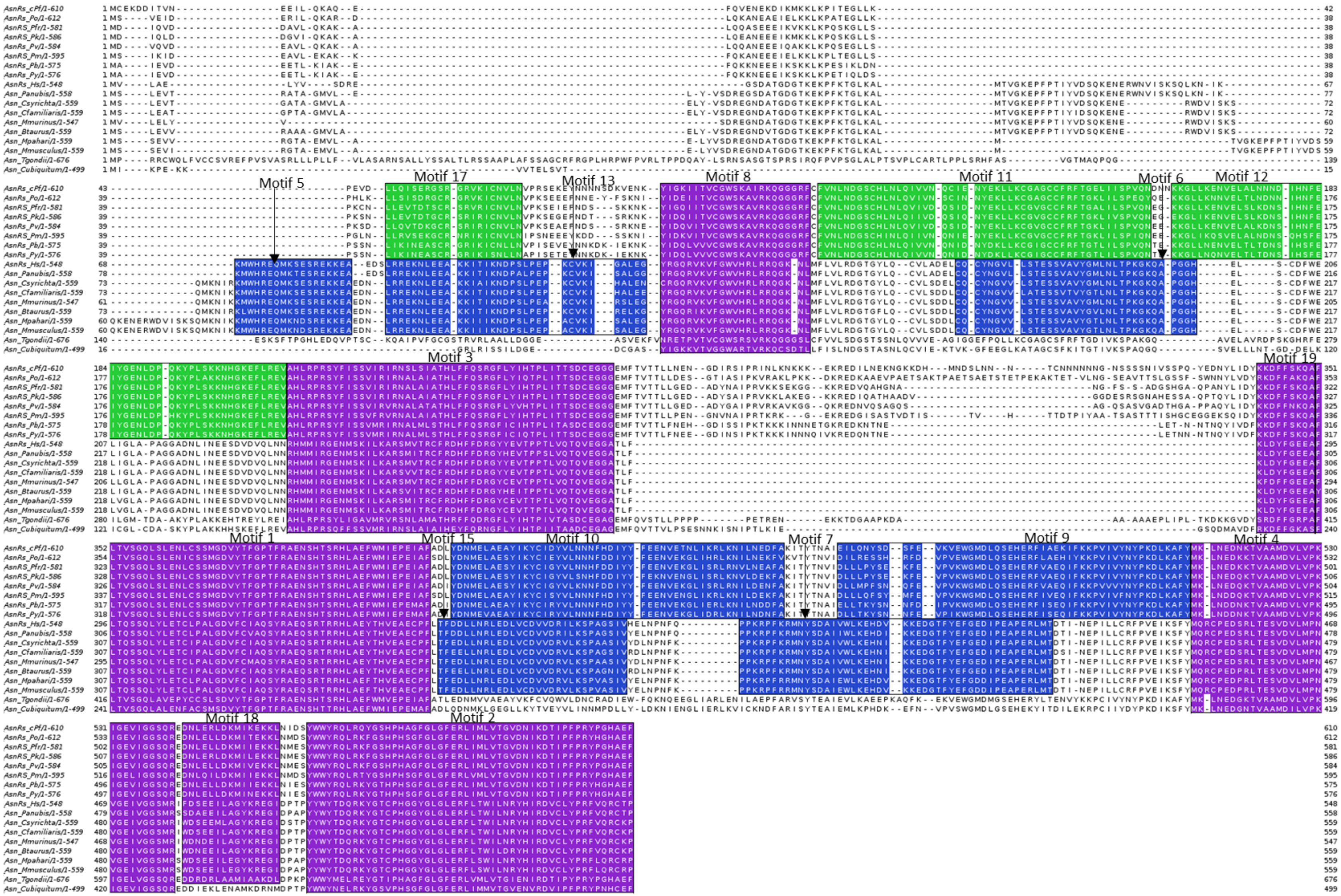

Additional file 4L: Mapping of motifs discovered in AsnRS family to the multiple sequence alignment

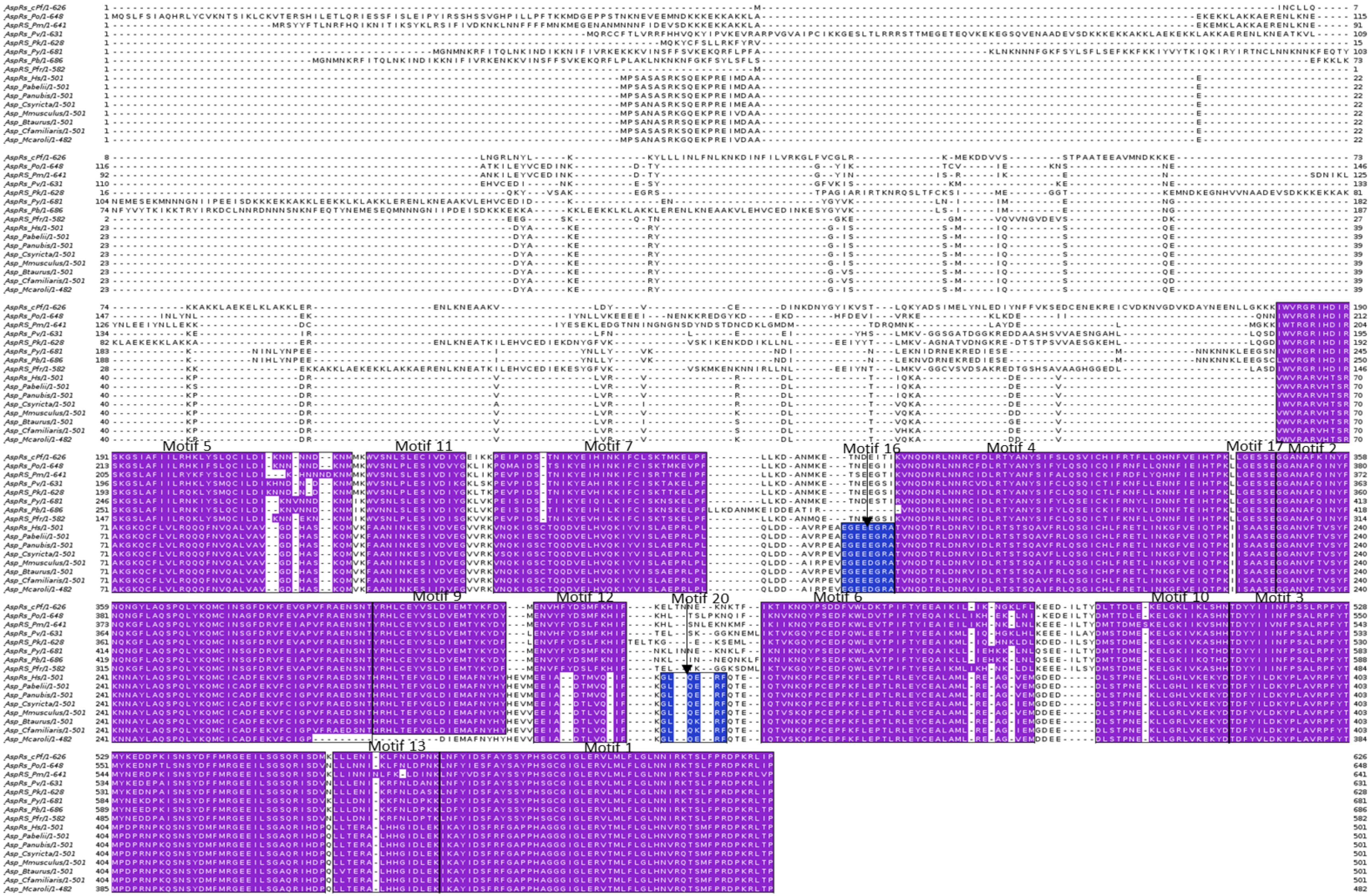

Additional file 4M: Mapping of motifs discovered in AspRS family to the multiple sequence alignment

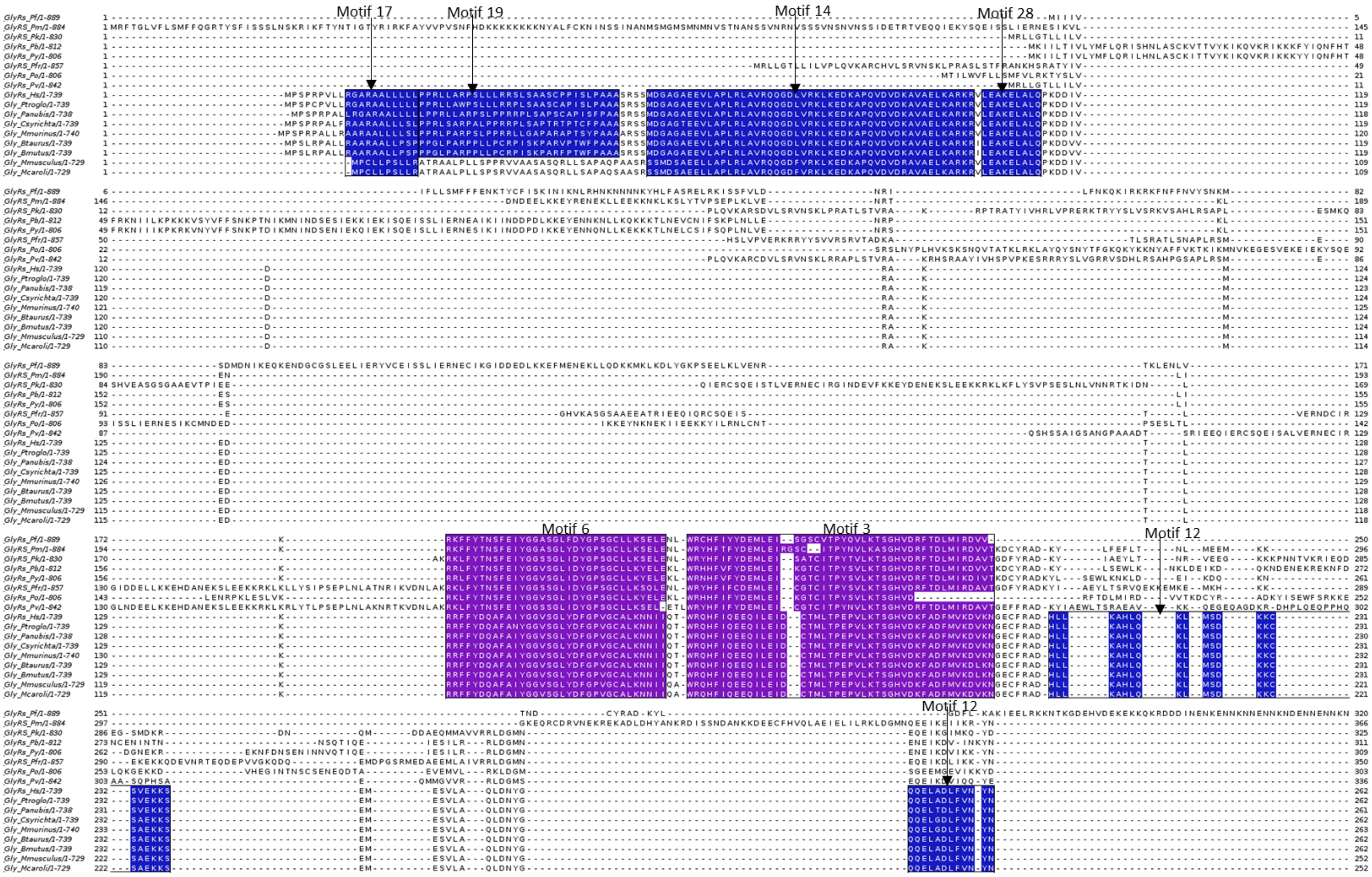

Additional file 4N: Mapping of motifs discovered in GlyRS family to the multiple sequence alignment

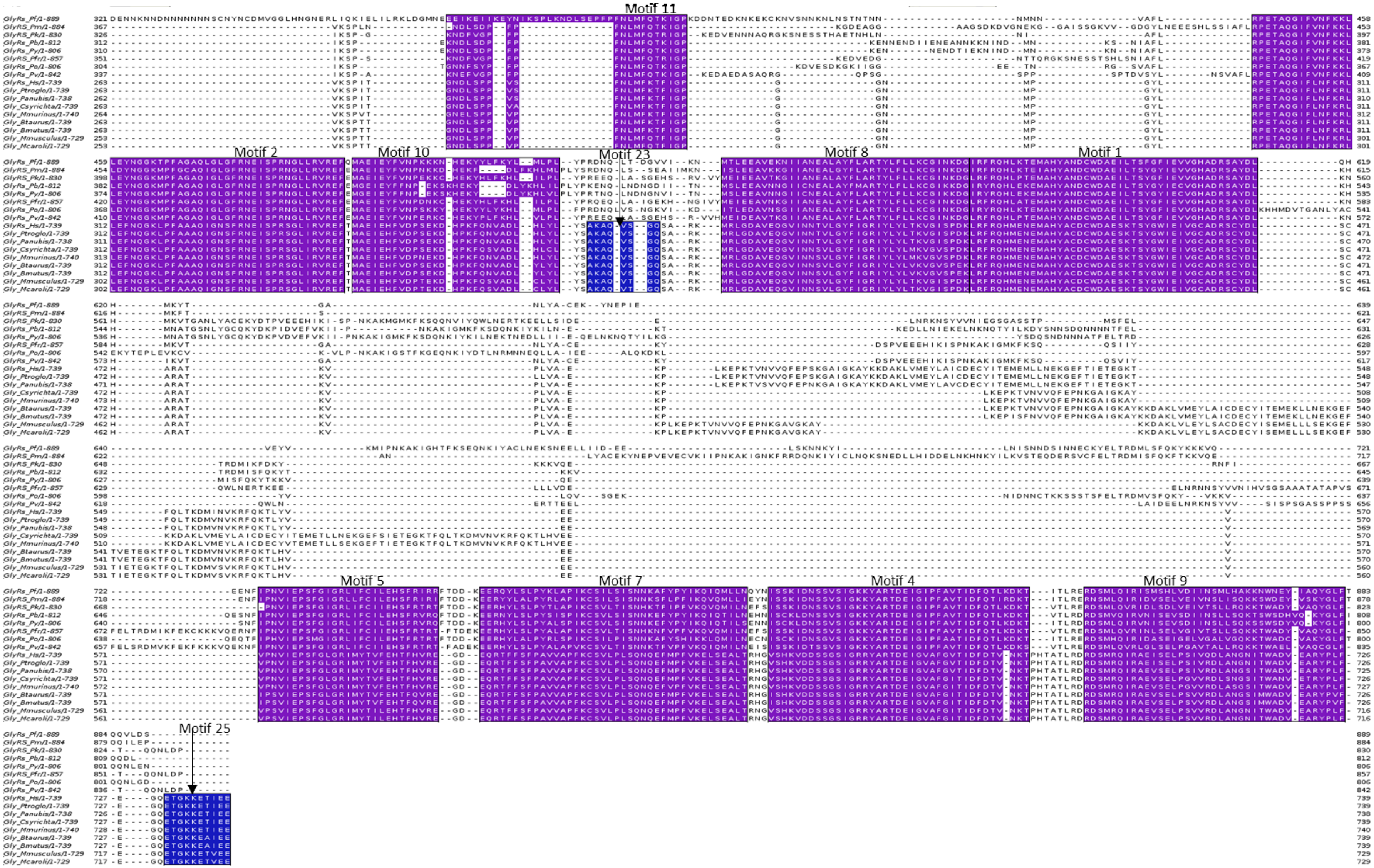

Additional file 4N: Mapping of motifs discovered in GlyRS family to the multiple sequence alignment

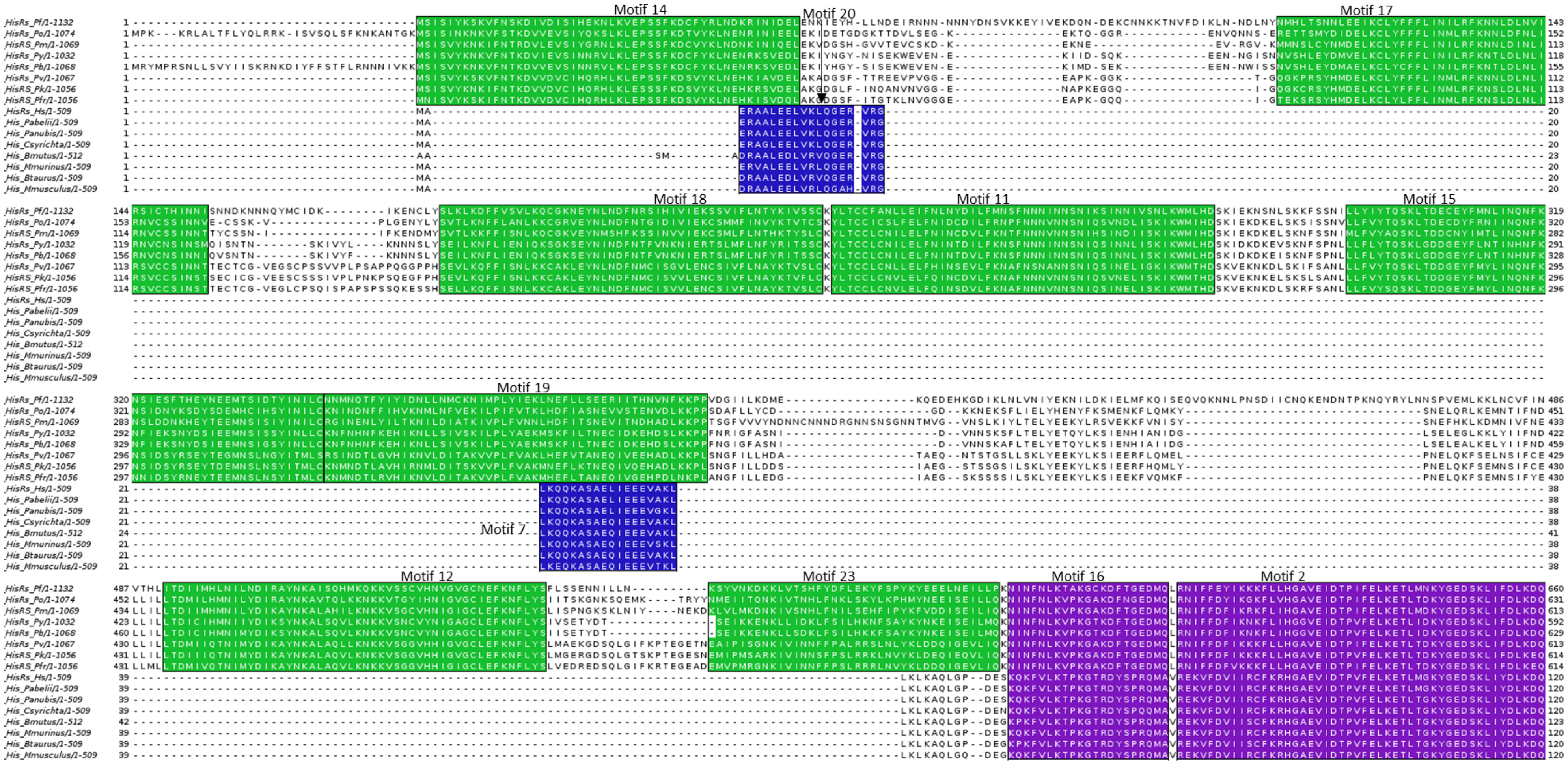

Additional file 4O: Mapping of motifs discovered in HisRS family to the multiple sequence alignment

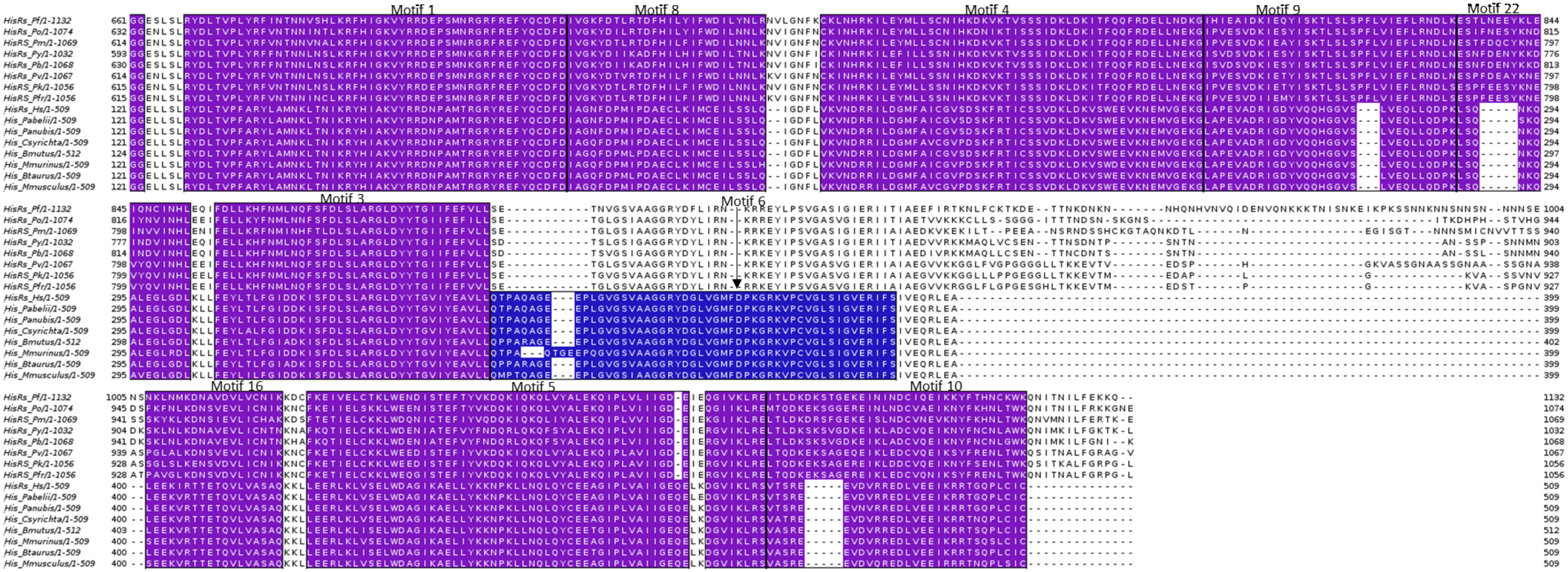

Additional file 4O: Mapping of motifs discovered in HisRS family to the multiple sequence alignment

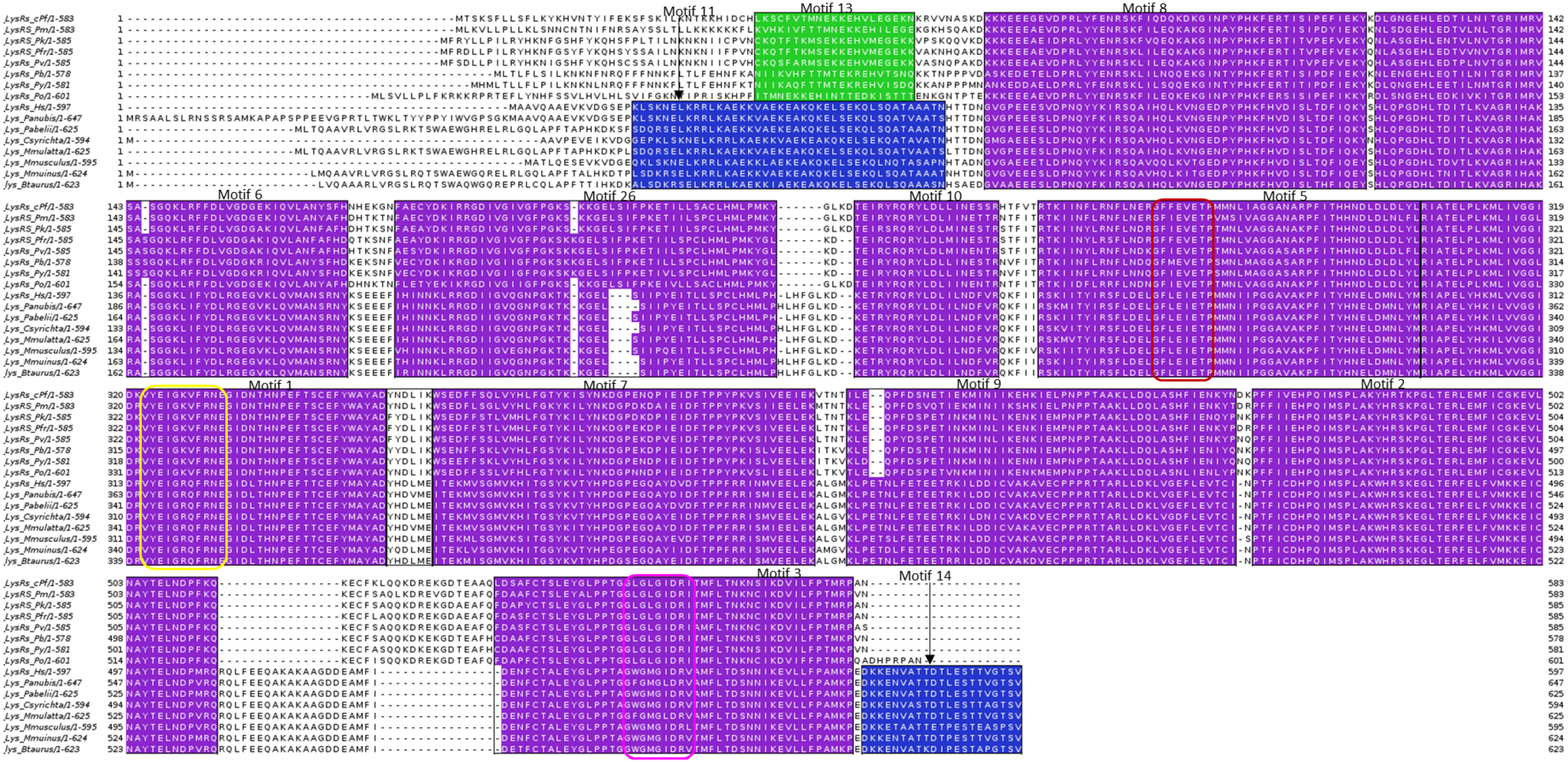

Additional file 4P: Mapping of motifs discovered in LysRS family to the multiple sequence alignment

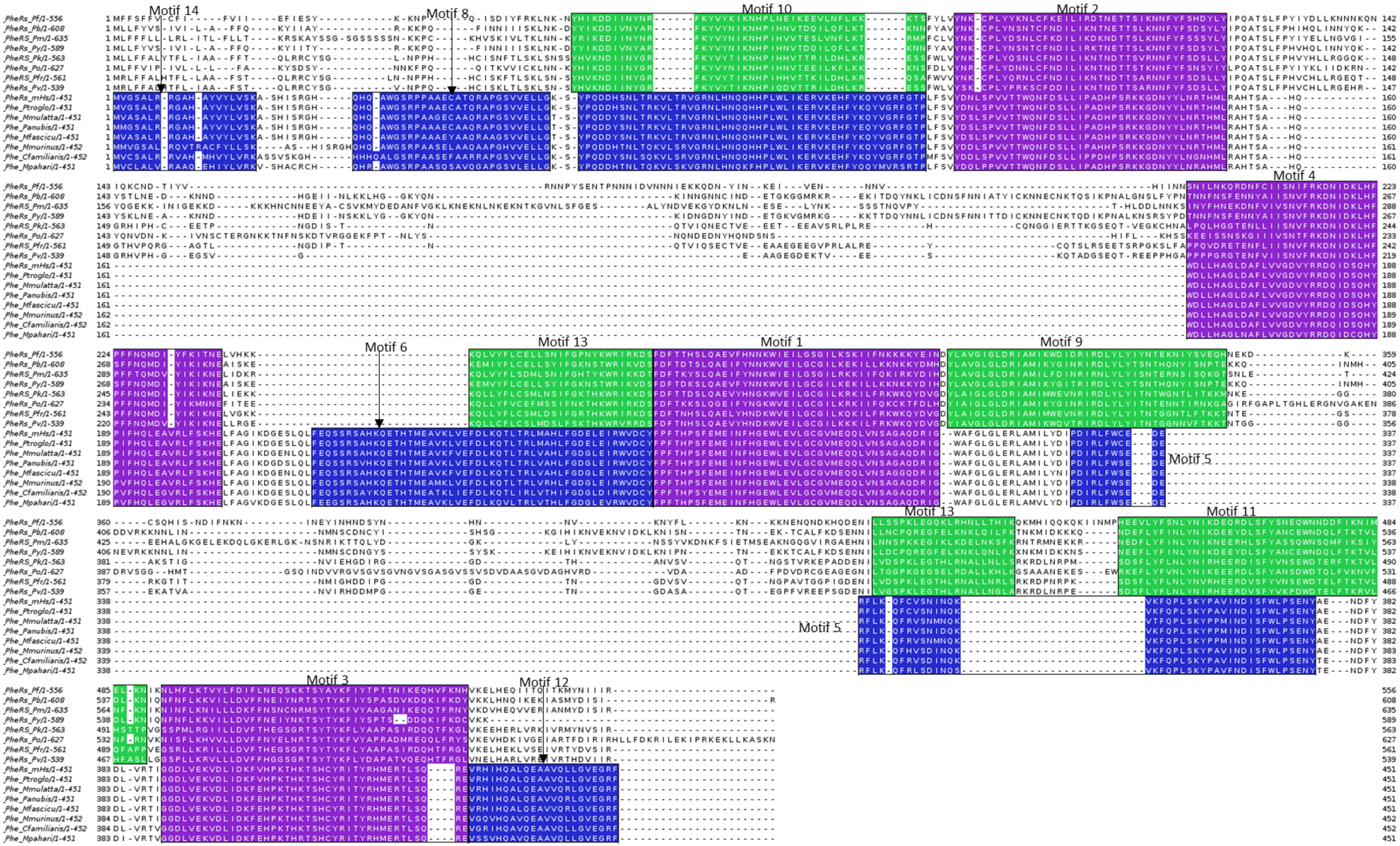

Additional file 4Q: Mapping of motifs discovered in PheRS family to the multiple sequence alignment

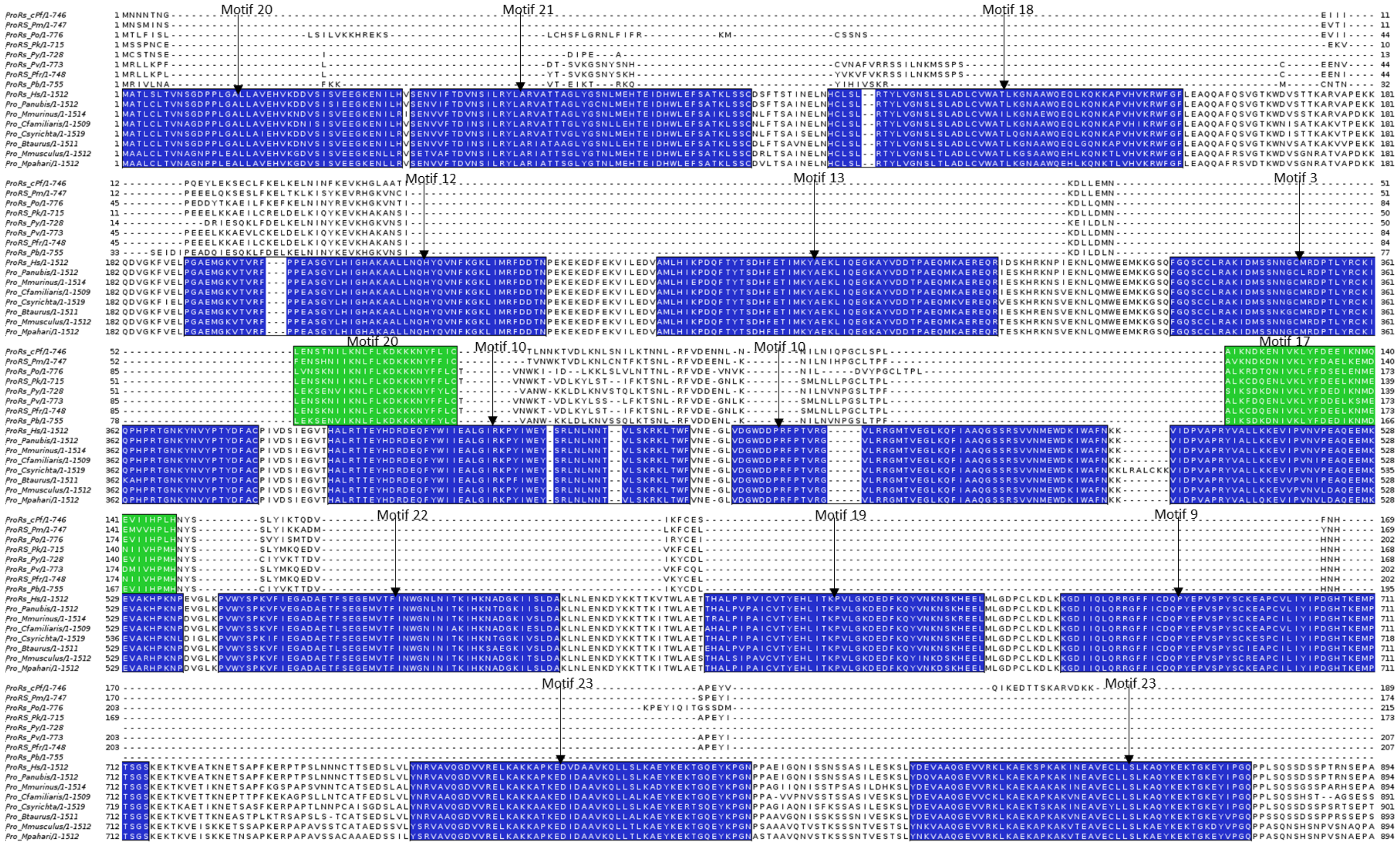

Additional file 4R: Mapping of motifs discovered in ProRS family to the multiple sequence alignment

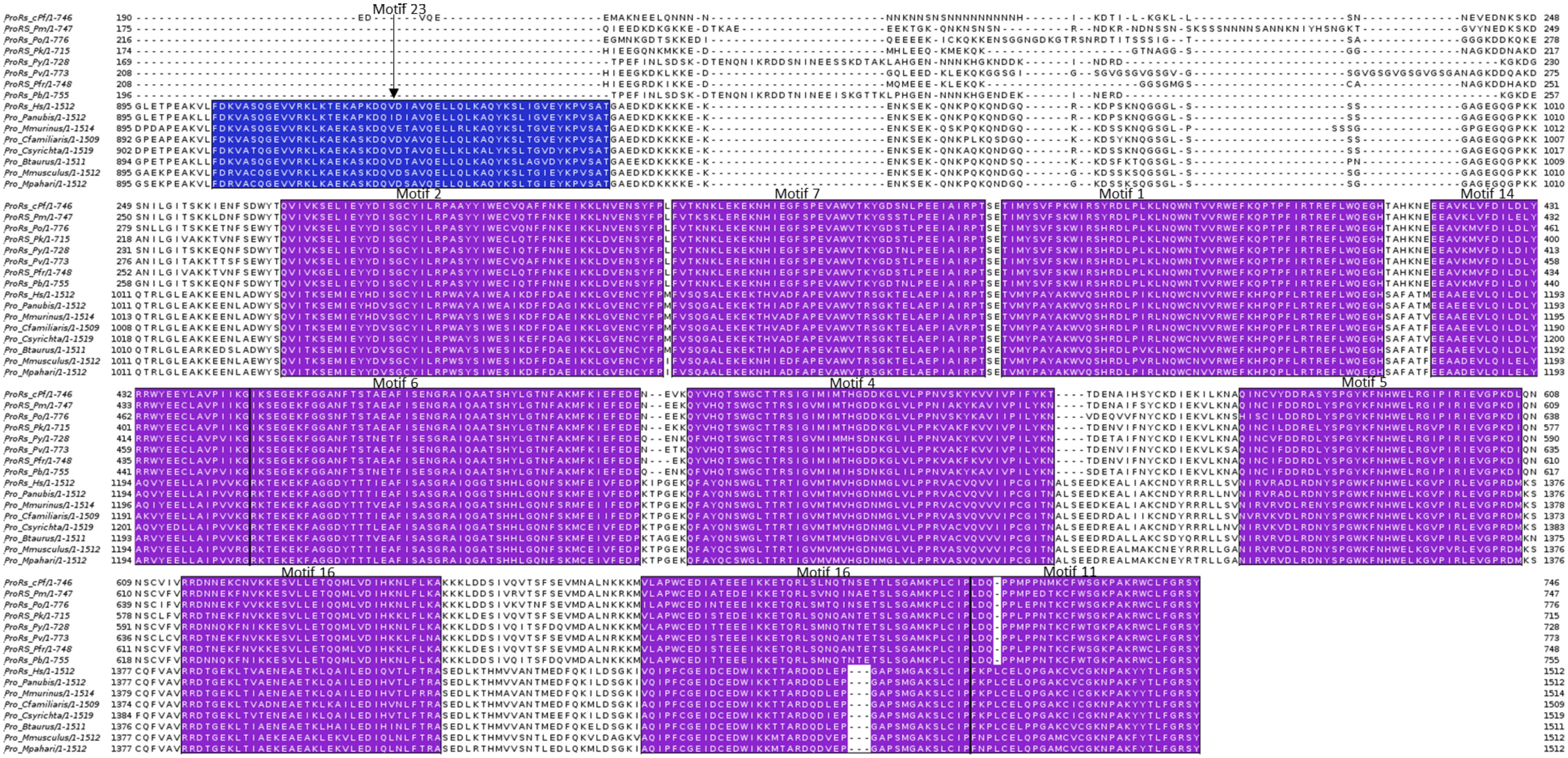

Additional file 4R: Mapping of motifs discovered in ProRS family to the multiple sequence alignment

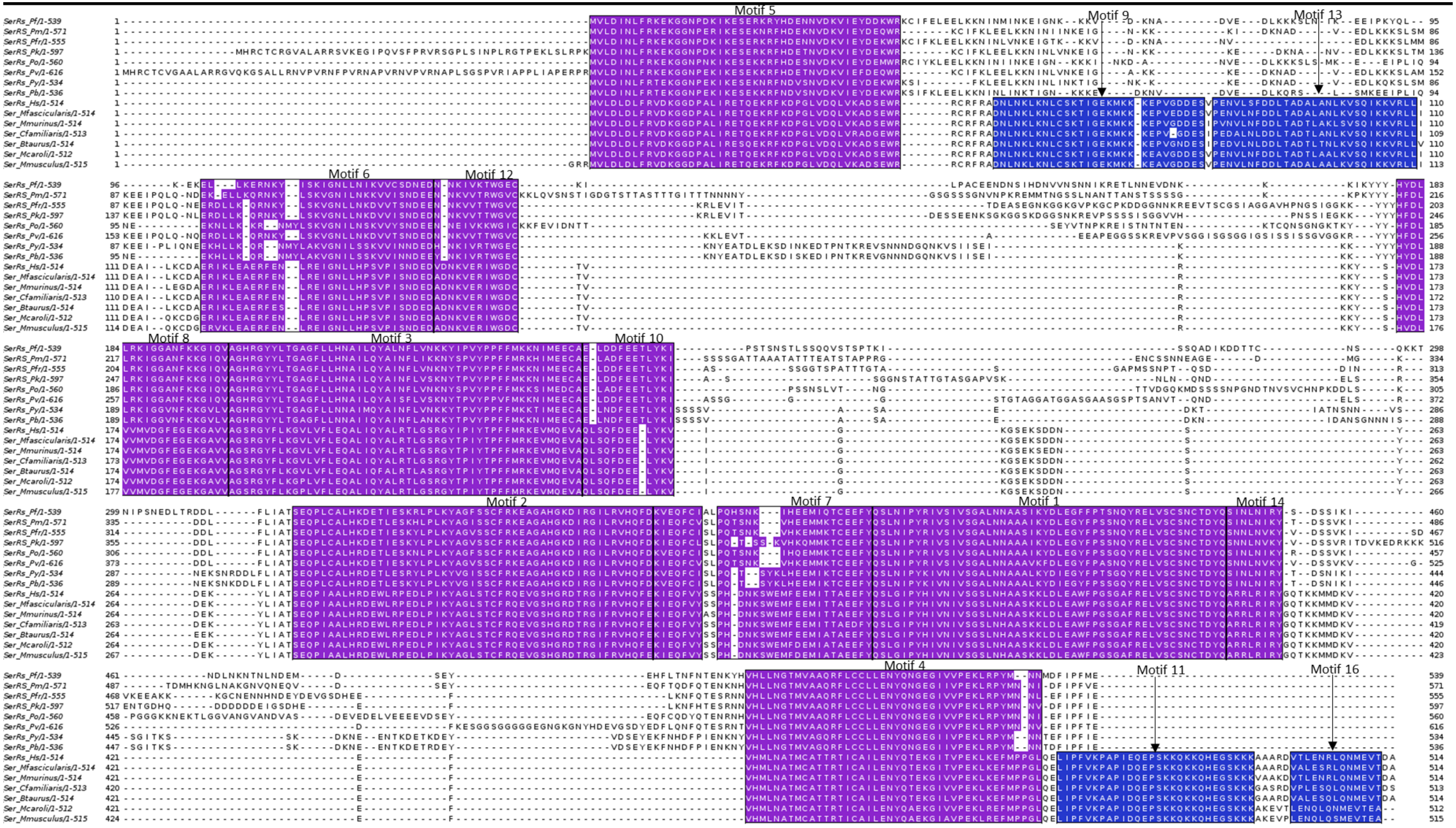

Additional file 4S: Mapping of motifs discovered in SerRS family to the multiple sequence alignment

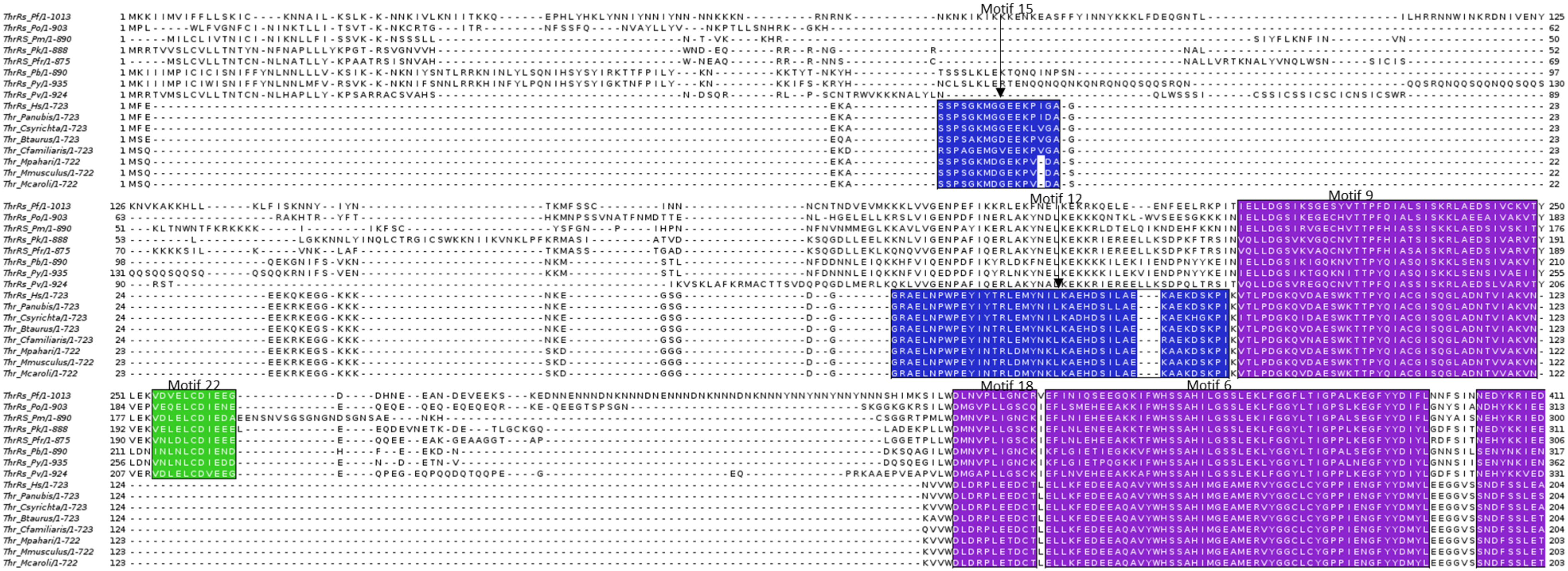

Additional file 4T: Mapping of motifs discovered in ThrRS family to the multiple sequence alignment

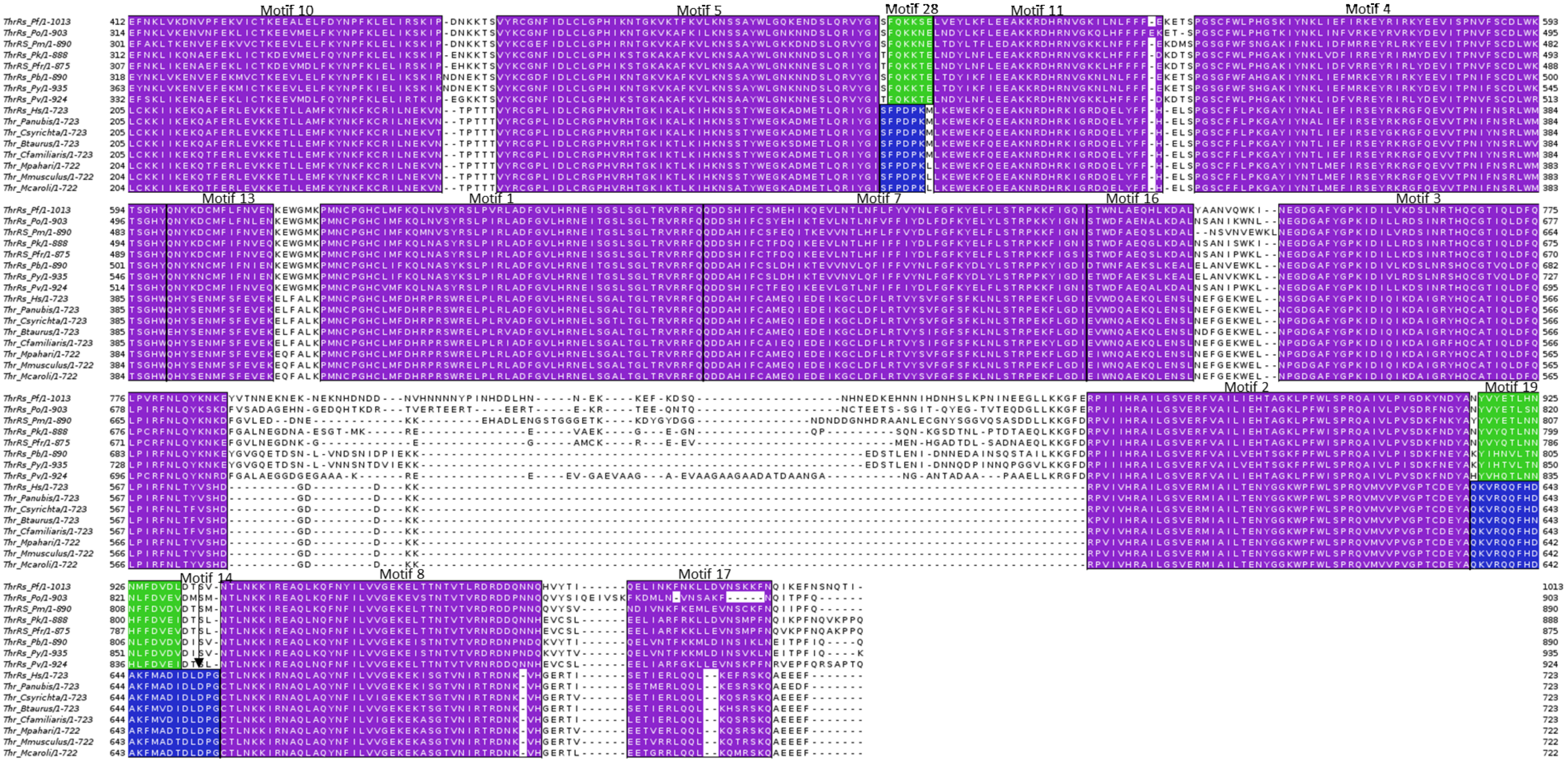

Additional file 47: Mapping of motifs discovered in ThrRS family to the multiple sequence alignment
