# Supplementary material for: Aminoacyl tRNA synthetases as malarial drug targets: a comparative bioinformatics study
Source: Malar J. 2019 Feb 6;18:34. doi: 10.1186/s12936-019-2665-6 (PMC6366043; doi:10.1186/s12936-019-2665-6)
Supplement: Supplementary file 5 — Additional file 5. Phylogenetic trees and pairwise sequence calculations for aaRS families: Molecular Phylogenetic calculations were performed using MEGA7. Sequence identity calculations were done using an in-house python script and results displayed as heatmaps. Conservation increases from blue to red. [file 12936_2019_2665_MOESM5_ESM.pdf]

Additional file 5: Phylogenetic trees and pairwise sequence calculations for aaRS families. Phylogenetic tree calculations were done using MEGA vs 7 while pairwise sequence calculations within each aaRS family were done using a Python script and results displayed as heatmaps using a Matlab script. A red color shows high sequence identity while a blue color shows low sequence identity.

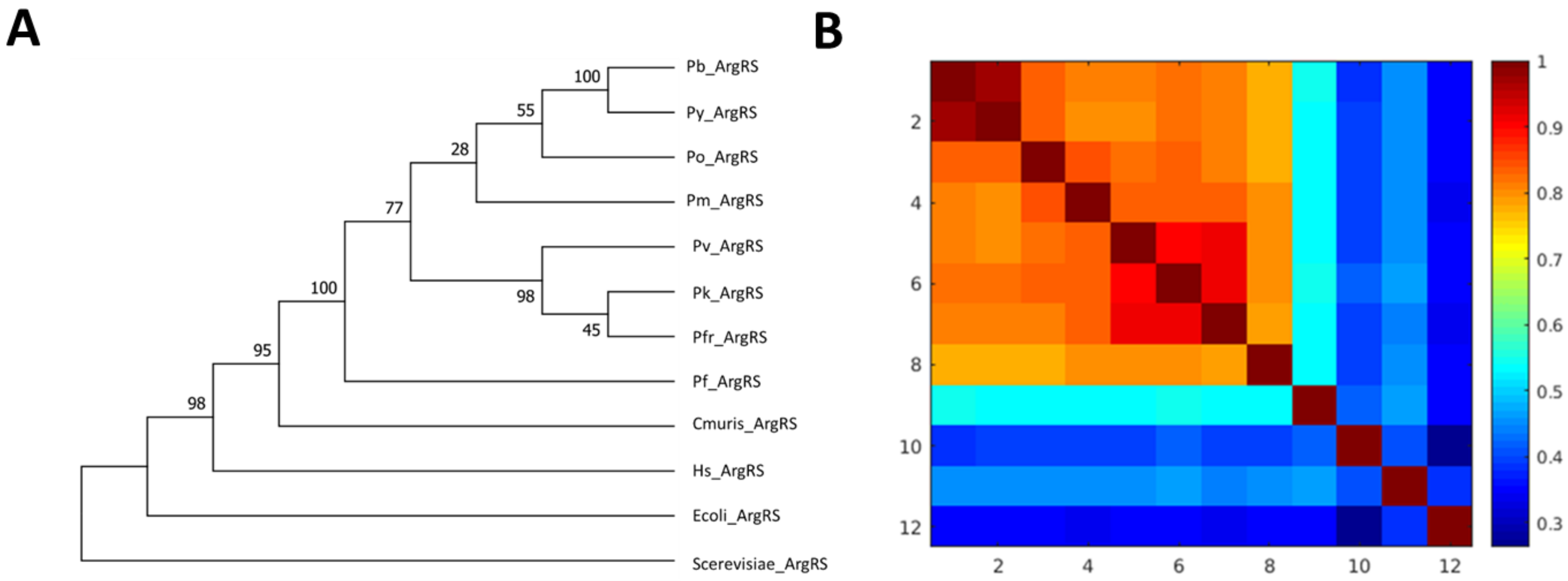

Additional file 5.1: A) ArgRS family phylogenetic tree. Maximum Likelihood method was used to infer evolutionary history using Le_Gascuel_2008 model at 90% site coverage. Phylogenetic tree calculations were done using MEGA7. The tree that had the highest log likelihood (-8602.47) is shown. The percentage of trees in which the associated taxa clustered together is shown next to the branches. Initial tree(s) for the heuristic search were obtained by using BioNJ and Neighbor-Join algorithms to a matrix of pairwise distances calculated using a JTT model, and then selecting the topology with higher log likelihood value. A Gamma distribution was used to calculate evolutionary rate differences among sites (5 categories (+*G*, parameter = 1.1184)). Twelve amino acid sequences were used for this analysis. There were 564 positions after calculations**. B)** ArgRS pairwise sequence calculations. The sequence identity values of the sequences in the ArgRS family is shown. The heatmap shows the identity scores as a color-coded matrix for every aaRS sequence versus every aaRS sequence in this family. Conservation increases from blue to red in the heat map.

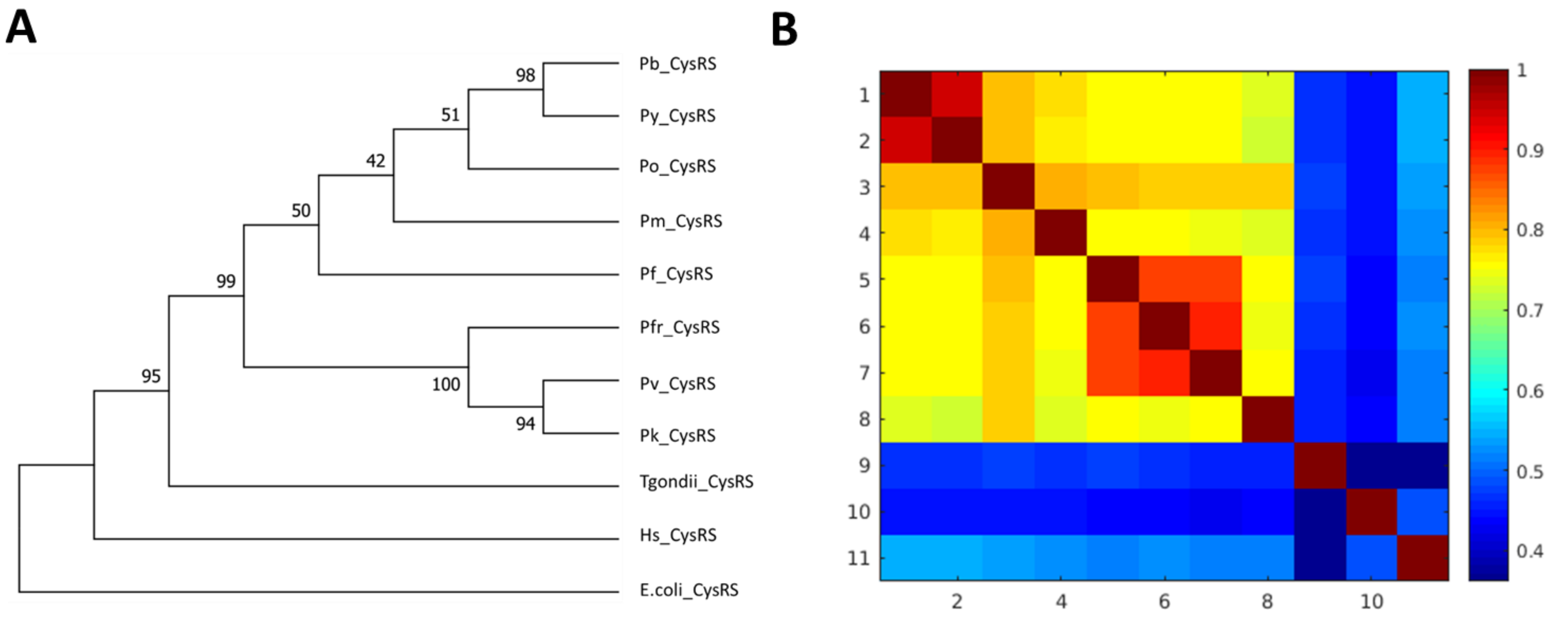

Additional file 5.2: A) CysRS family phylogenetic tree. Maximum Likelihood method was used to infer evolutionary history using Le_Gascuel_2008 model at 95% site coverage. Phylogenetic tree calculations were done using MEGA7. The tree that had the highest log likelihood (-4877.44) is shown. Initial tree(s) for the heuristic search were obtained by using BioNJ and Neighbor-Join algorithms to a matrix of pairwise distances calculated using a JTT model, and then selecting the topology with higher log likelihood value. A Gamma distribution was used to calculate evolutionary rate differences among sites (5 categories (+*G*, parameter = 0.7090)). Eleven amino acid sequences were used for this analysis. There were 442 positions after calculations. **B)** CysRS pairwise sequence calculations. The sequence identity values of the sequences in the CysRS family is shown. The heatmap shows the identity scores as a color-coded matrix for every aaRS sequence versus every aaRS sequence in this family. Conservation increases from blue to red in the heat map.

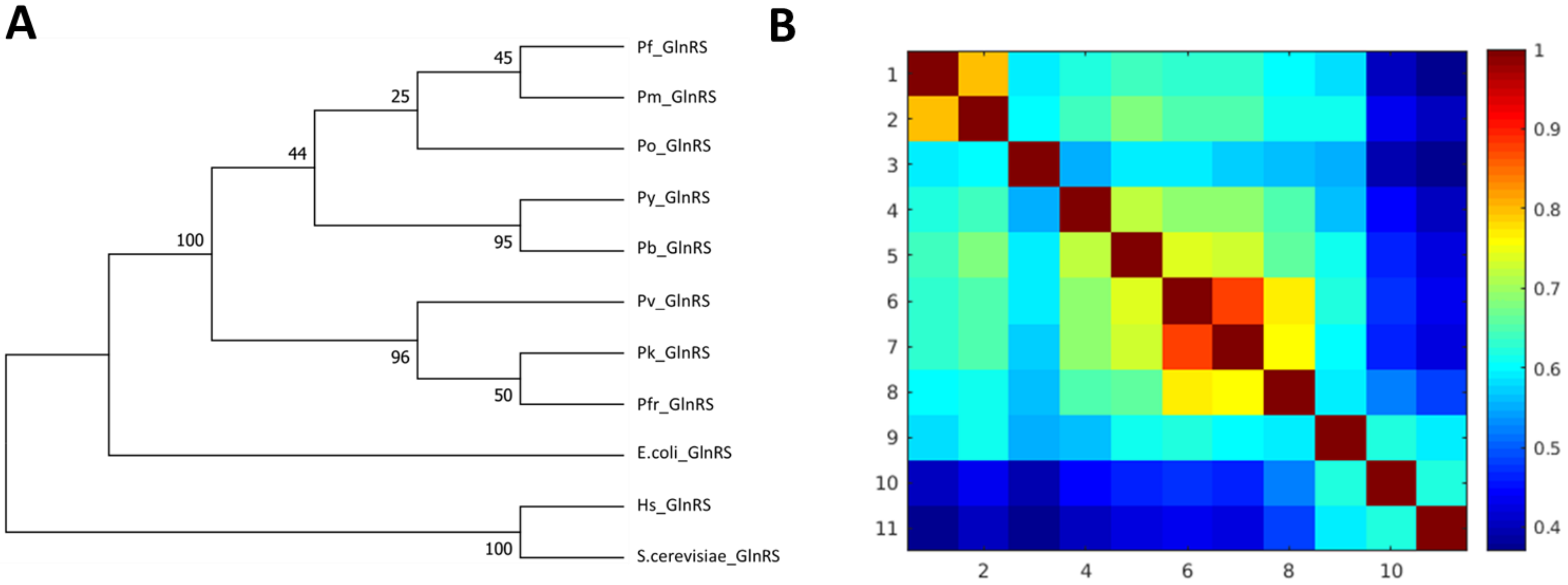

Additional file 5.3: A) GlnRS family phylogenetic tree. Maximum Likelihood method was used to infer evolutionary history using Le_Gascuel_2008 model at 100% site coverage. Phylogenetic tree calculations were done using MEGA7. The tree that had the highest log likelihood (-5809.90) is shown. Initial tree(s) for the heuristic search were obtained by using BioNJ and Neighbor-Join algorithms to a matrix of pairwise distances calculated using a JTT model, and then selecting the topology with higher log likelihood value. A Gamma distribution was used to calculate evolutionary rate differences among sites (5 categories (+*G*, parameter = 0.6210)). Eleven amino acid sequences were used for this analysis. There were 484 positions after calculations. **B)** GlnRS pairwise sequence calculations. The sequence identity values of the sequences in the GlnRS family is shown. The heatmap shows the identity scores as a color-coded matrix for every aaRS sequence versus every aaRS sequence in this family. Conservation increases from blue to red in the heat map.

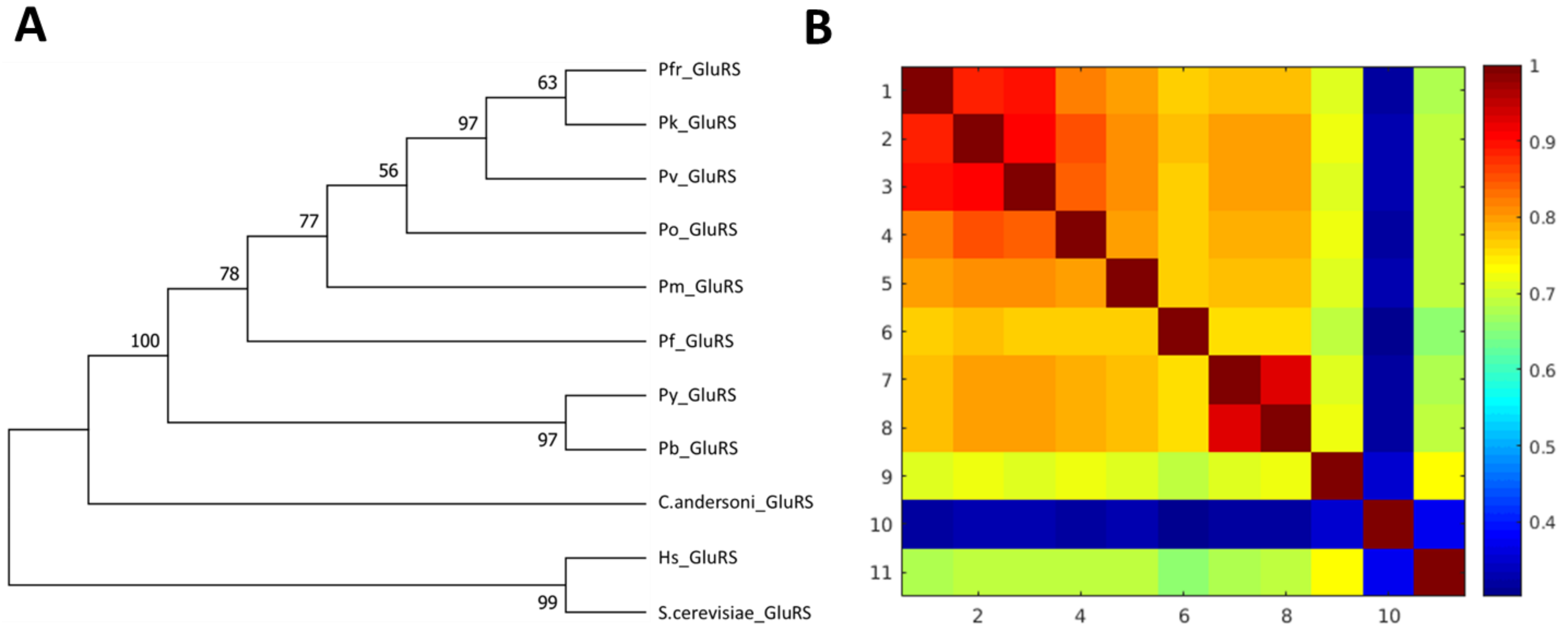

Additional file 5.4: A) GluRS family phylogenetic tree. Maximum Likelihood method was used to infer evolutionary history using Whelan And Goldman model at 100% site coverage. Phylogenetic tree calculations were done using MEGA7. The tree that had the highest log likelihood (-4983.29) is shown. Initial tree(s) for the heuristic search were obtained by using BioNJ and Neighbor-Join algorithms to a matrix of pairwise distances calculated using a JTT model, and then selecting the topology with higher log likelihood value. A Gamma distribution was used to calculate evolutionary rate differences among sites (5 categories (+*G*, parameter = 0.8404)). Eleven amino acid sequences were used for this analysis. There were of 430 positions after calculations**. B)** GluRS pairwise sequence calculations. The sequence identity values of the sequences in the GluRS family is shown. The heatmap shows the identity scores as a color-coded matrix for every aaRS sequence versus every aaRS sequence in this family. Conservation increases from blue to red in the heat map.

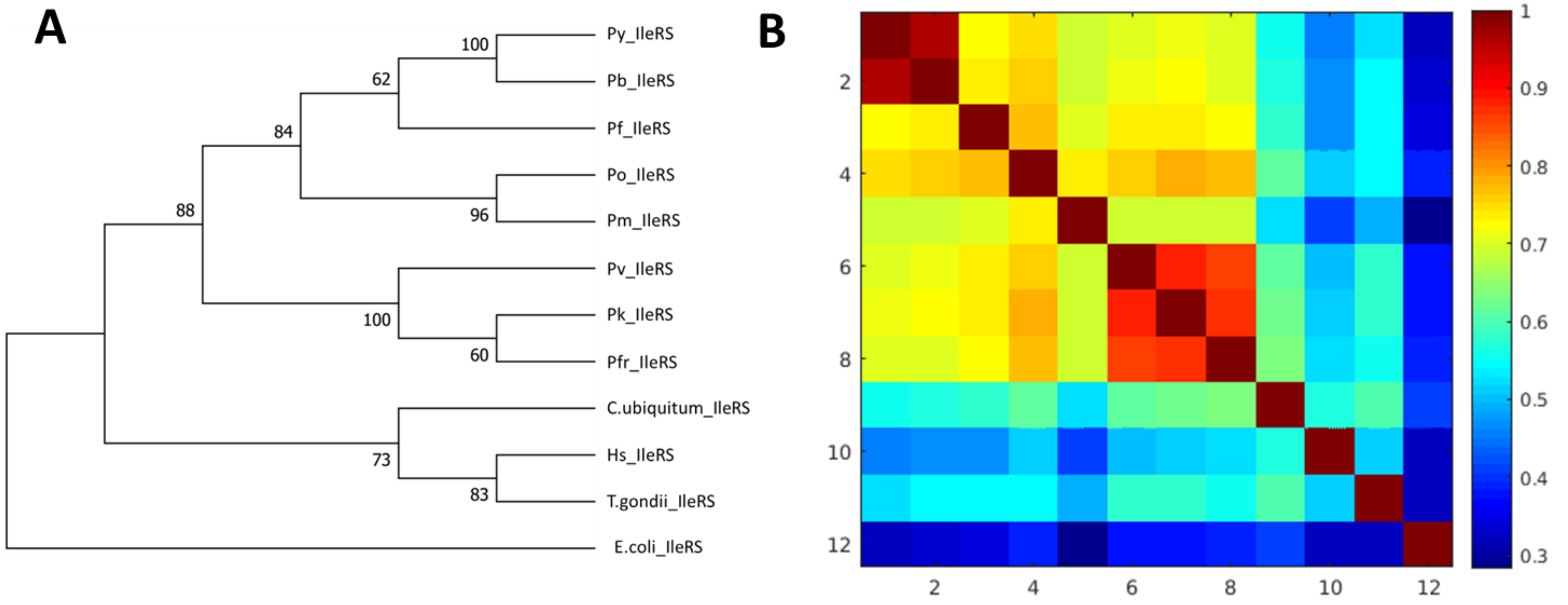

Additional file 5.5: A) IleRS family phylogenetic tree. Maximum Likelihood method was used to infer evolutionary history using Le_Gascuel_2008 model at 90% site coverage. Phylogenetic tree calculations were done using MEGA7. The tree that had the highest log likelihood (-15380.82) is shown. Initial tree(s) for the heuristic search were obtained by using BioNJ and Neighbor-Join algorithms to a matrix of pairwise distances calculated using a JTT model, and then selecting the topology with higher log likelihood value. A Gamma distribution was used to calculate evolutionary rate differences among sites (5 categories (+*G*, parameter = 0.7449)). Twelve amino acid sequences were used for this analysis. There were of 1073 positions after calculations**. B)** IleRS pairwise sequence calculations. The sequence identity values of the sequences in the IleRS family is shown. The heatmap shows the identity scores as a color-coded matrix for every aaRS sequence versus every aaRS sequence in this family. Conservation increases from blue to red in the heat map.

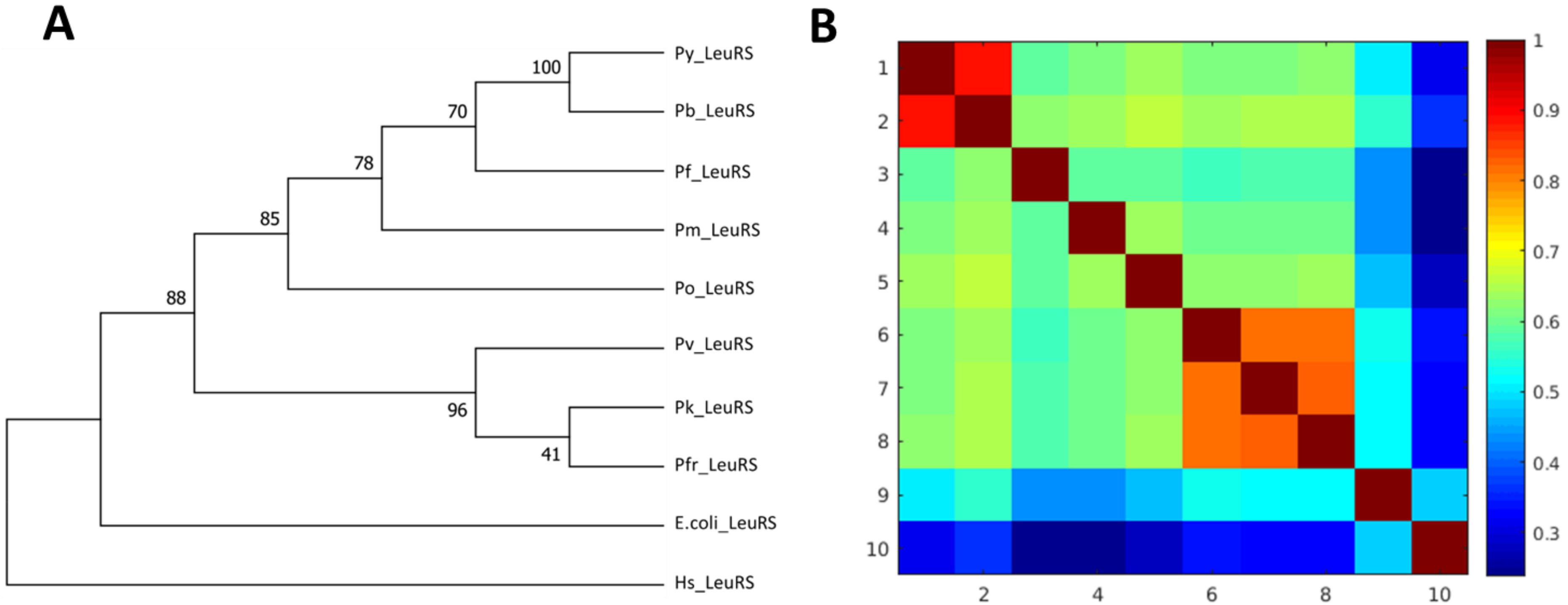

Additional file 5.6: A) LeuRS family phylogenetic tree. Maximum Likelihood method was used to infer evolutionary history using JTT matrix-based model at 90% site coverage. Phylogenetic tree calculations were done using MEGA7. The tree that had the highest log likelihood (-11798.31) is shown. Initial tree(s) for the heuristic search were obtained by using BioNJ and Neighbor-Join algorithms to a matrix of pairwise distances calculated using a JTT model, and then selecting the topology with higher log likelihood value. A Gamma distribution was used to calculate evolutionary rate differences among sites (5 categories (+*G*, parameter = 1.0104)). Ten amino acid sequences were used for this analysis. There were 839 positions after calculations**. B)** LeuRS pairwise sequence calculations. The sequence identity values of the sequences in the LeuRS family is shown. The heatmap shows the identity scores as a color-coded matrix for every aaRS sequence versus every aaRS sequence in this family. Conservation increases from blue to red in the heat map.

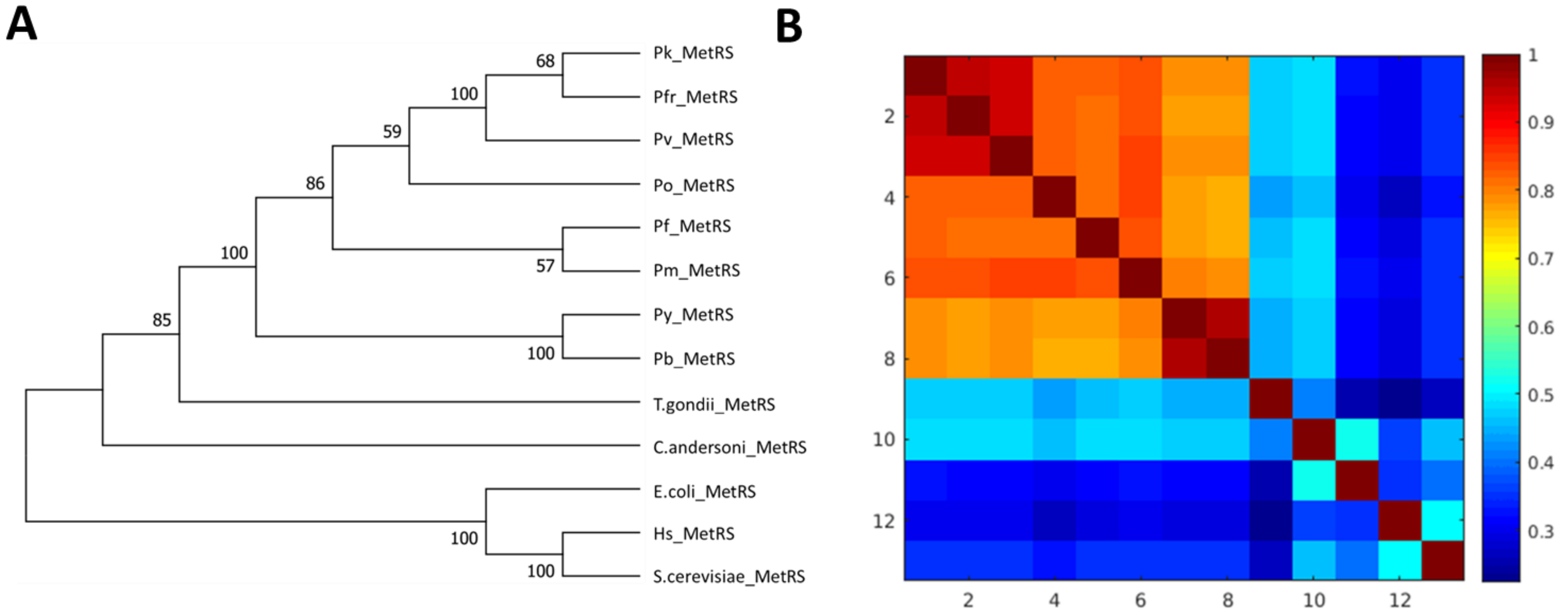

Additional file 5.7: A) MetRS family phylogenetic tree. Maximum Likelihood method was used to infer evolutionary history using Le_Gascuel_2008 model at 90% site coverage. Phylogenetic tree calculations were done using MEGA7. The tree that had the highest log likelihood (-8999.21) is shown. Initial tree(s) for the heuristic search were obtained by using BioNJ and Neighbor-Join algorithms to a matrix of pairwise distances calculated using a JTT model, and then selecting the topology with higher log likelihood value. A Gamma distribution was used to calculate evolutionary rate differences among sites (5 categories (+*G*, parameter = 1.5029)). The rate variation model allowed for some sites to be evolutionarily invariable ([+*I*], 4.21% sites). Thirteen amino acid sequences were used for this analysis. There were 534 positions after calculations. **B)** MetRS pairwise sequence calculations. The sequence identity values of the sequences in the MetRS family is shown. The heatmap shows the identity scores as a color-coded matrix for every aaRS sequence versus every aaRS sequence in this family. Conservation increases from blue to red in the heat map.

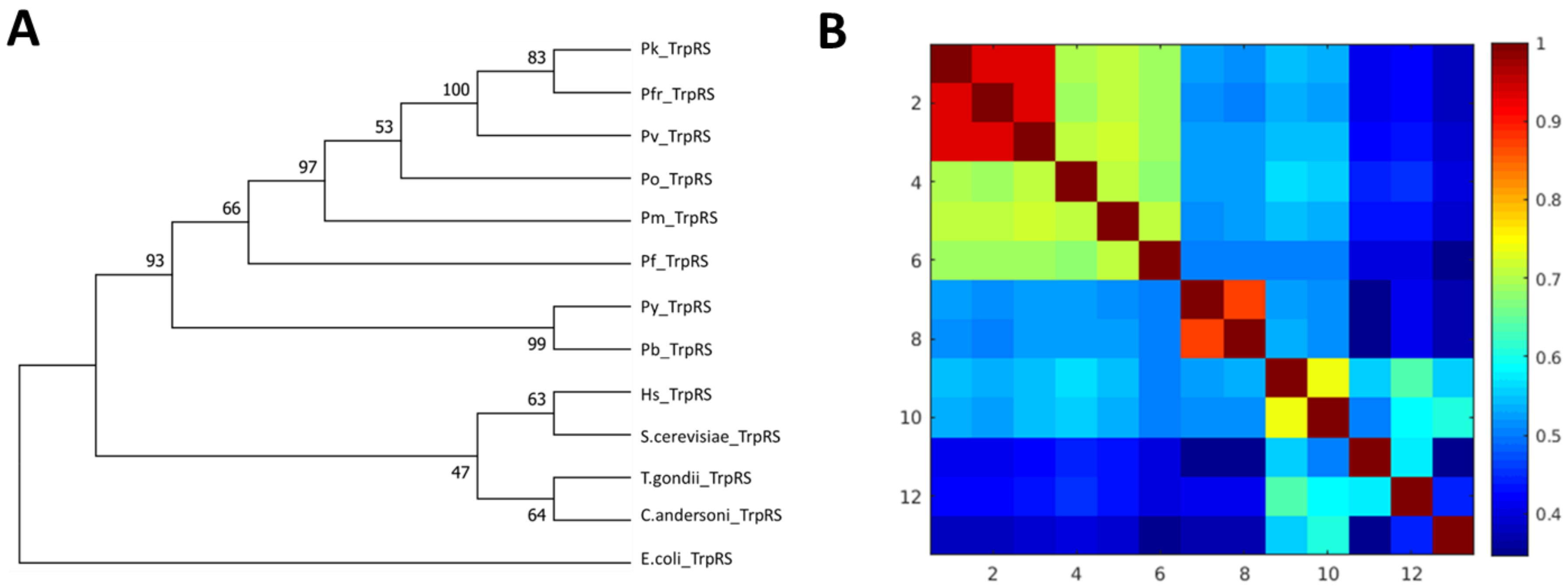

Additional file 5.8: A) TrpRS family phylogenetic tree. Maximum Likelihood method was used to infer evolutionary history using Le_Gascuel_2008 model at 90% site coverage. Phylogenetic tree calculations were done using MEGA7. The tree that had the highest log likelihood (-6377.66) is shown. Initial tree(s) for the heuristic search were obtained by using BioNJ and Neighbor-Join algorithms to a matrix of pairwise distances calculated using a JTT model, and then selecting the topology with higher log likelihood value. A Gamma distribution was used to calculate evolutionary rate differences among sites (5 categories (+*G*, parameter = 1.5029)). The rate variation model allowed for some sites to be evolutionarily invariable ([+*I*], 5.47% sites). Thirteen amino acid sequences were used for this analysis. There were 411 positions after calculations. **B)** TrpRS pairwise sequence calculations. The sequence identity values of the sequences in the TrpRS family is shown. The heatmap shows the identity scores as a color-coded matrix for every aaRS sequence versus every aaRS sequence in this family. Conservation increases from blue to red in the heat map.

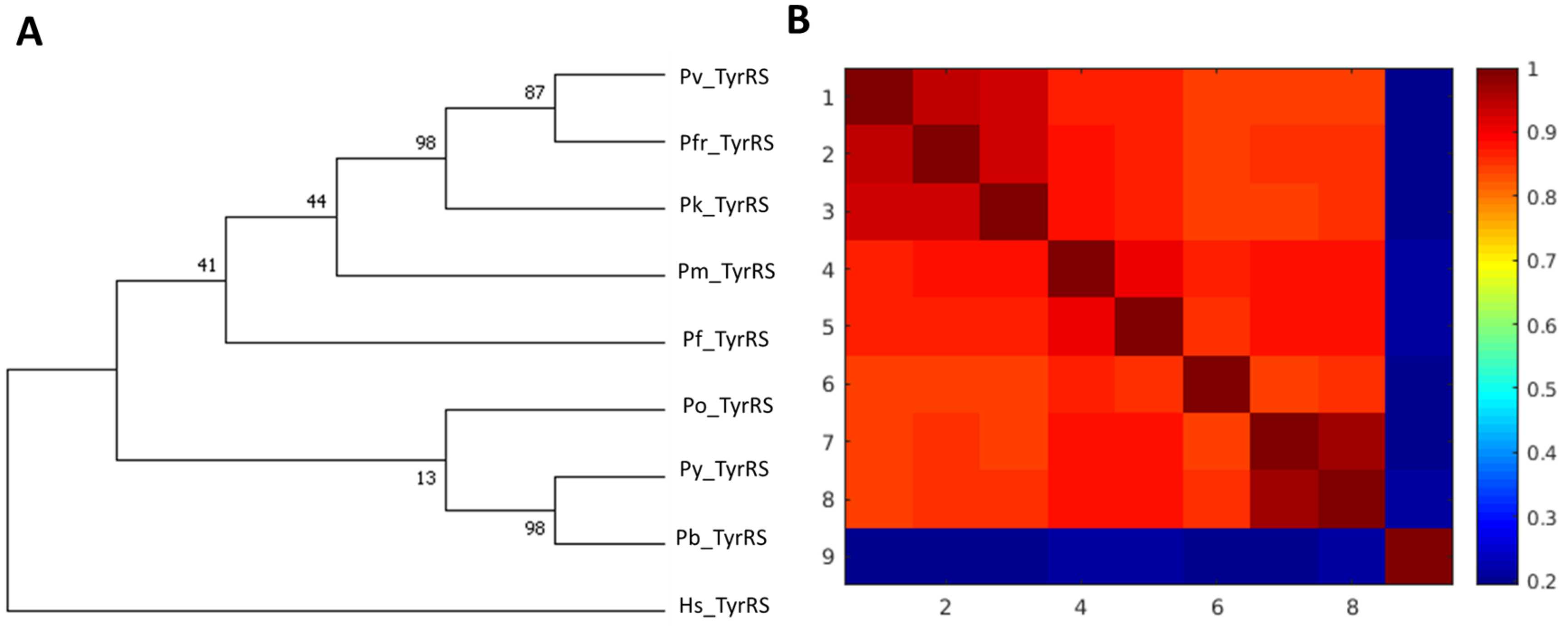

Additional file 5.9: A) TyrRS family phylogenetic tree. Maximum Likelihood method was used to infer evolutionary history using Le_Gascuel_2008 model at 95% site coverage. Phylogenetic tree calculations were done using MEGA7. The tree that had the highest log likelihood (-2978.09) is shown. Initial tree(s) for the heuristic search were obtained by using BioNJ and Neighbor-Join algorithms to a matrix of pairwise distances calculated using a JTT model, and then selecting the topology with higher log likelihood value. A Gamma distribution was used to calculate evolutionary rate differences among sites (5 categories (+*G*, parameter = 0.4355)). Nine amino acid sequences were used for this analysis. There were 343 positions after the calculations**. B)** TyrRS pairwise sequence calculations. The sequence identity values of the sequences in the TyrRS family is shown. The heatmap shows the identity scores as a color-coded matrix for every aaRS sequence versus every aaRS sequence in this family. Conservation increases from blue to red in the heat map.

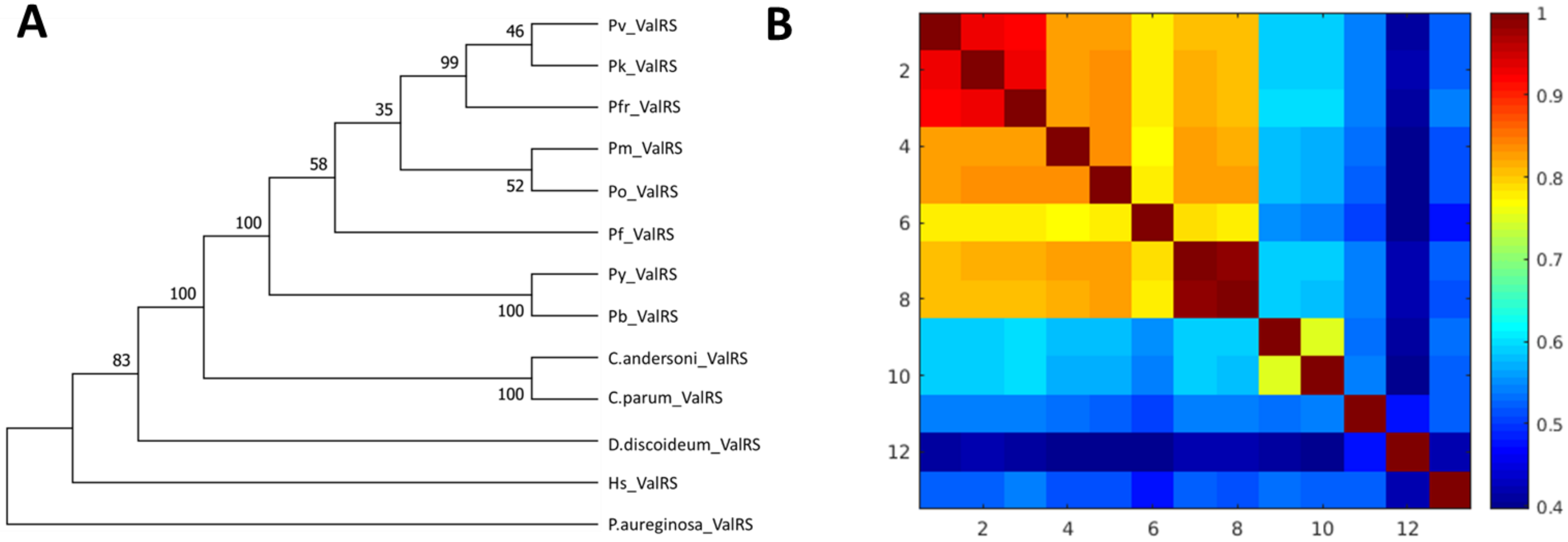

Additional file 5.10: A) ValRS family phylogenetic tree. Maximum Likelihood method was used to infer evolutionary history using Le_Gascuel_2008 model at 100% site coverage. Phylogenetic tree calculations were done using MEGA7. The tree that had the highest log likelihood (-12839.45) is shown. Initial tree(s) for the heuristic search were obtained by using BioNJ and Neighbor-Join algorithms to a matrix of pairwise distances calculated using a JTT model, and then selecting the topology with higher log likelihood value. A Gamma distribution was used to calculate evolutionary rate differences among sites (5 categories (+*G*, parameter = 0.6531)). Thirteen amino acid sequences were used for this analysis. There were 905 positions after the calculations**. B)** ValRS pairwise sequence calculations. The sequence identity values of the sequences in the ValRS family is shown. The heatmap shows the identity scores as a color-coded matrix for every aaRS sequence versus every aaRS sequence in this family. Conservation increases from blue to red in the heat map.

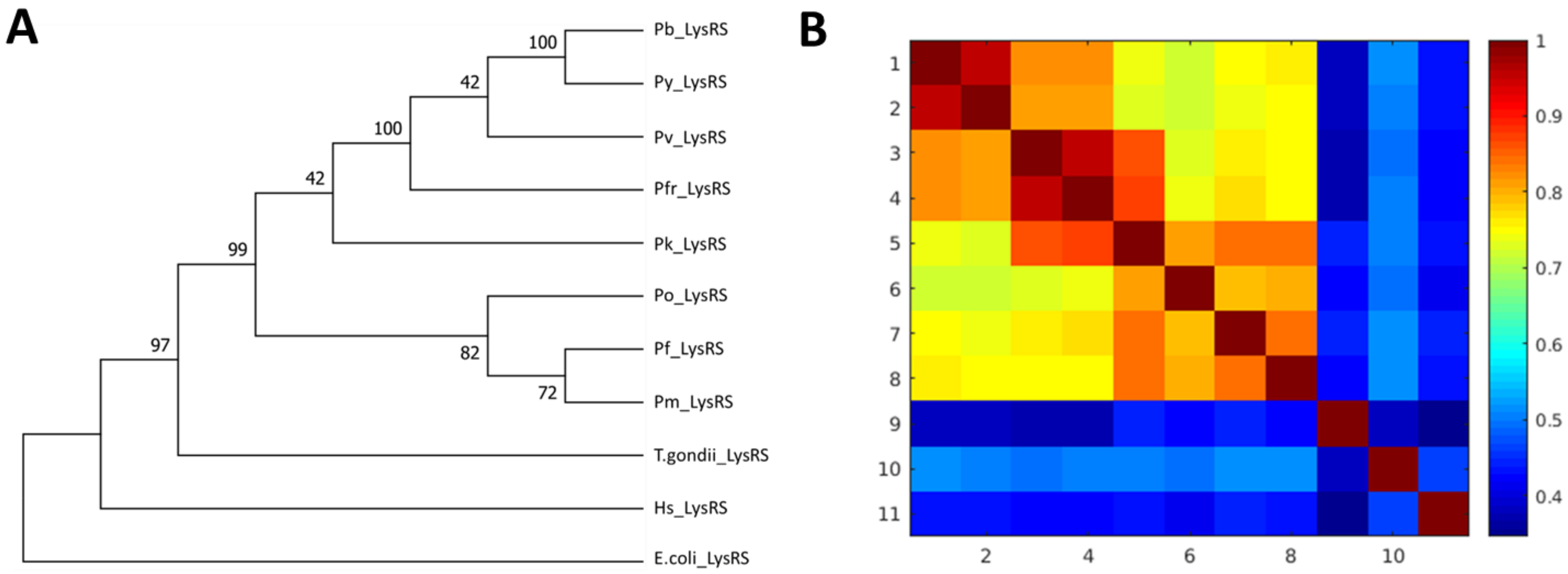

Additional file 5.11: A) LysRS family phylogenetic tree. Maximum Likelihood method was used to infer evolutionary history using Le_Gascuel_2008 model at 90% site coverage. Phylogenetic tree calculations were done using MEGA7. The tree that had the highest log likelihood (-6116.25) is shown. Initial tree(s) for the heuristic search were obtained by using BioNJ and Neighbor-Join algorithms to a matrix of pairwise distances calculated using a JTT model, and then selecting the topology with higher log likelihood value. A Gamma distribution was used to calculate evolutionary rate differences among sites (5 categories (+*G*, parameter = 0.6075)). Eleven amino acid sequences were used for this analysis. There were 503 positions after the calculations. **B)** LysRS pairwise sequence calculations. The sequence identity values of the sequences in the LysRS family is shown. The heatmap shows the identity scores as a color-coded matrix for every aaRS sequence versus every aaRS sequence in this family. Conservation increases from blue to red in the heat map.

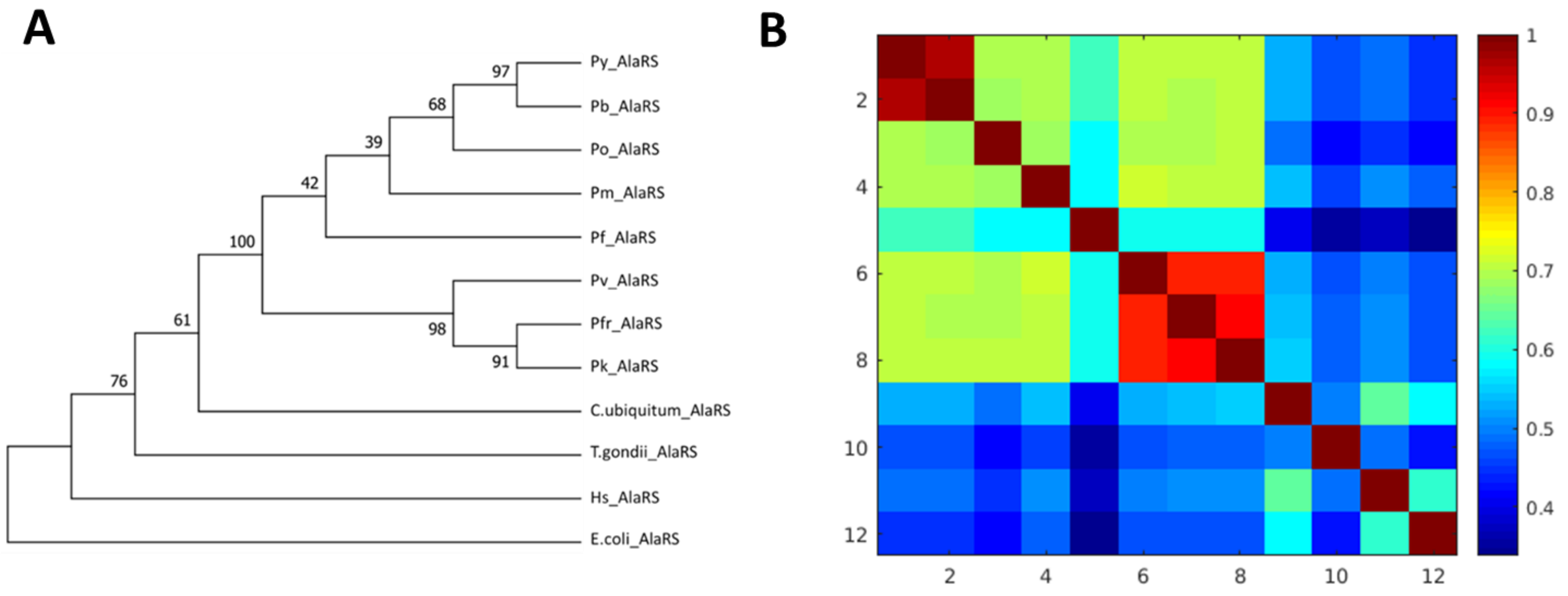

Additional file 5.12: A) AlaRS family phylogenetic tree. Maximum Likelihood method was used to infer evolutionary history using Le_Gascuel_2008 model at 90% site coverage. Phylogenetic tree calculations were done using MEGA7. The tree that had the highest log likelihood (-13324.59) is shown. Initial tree(s) for the heuristic search were obtained by using BioNJ and Neighbor-Join algorithms to a matrix of pairwise distances calculated using a JTT model, and then selecting the topology with higher log likelihood value. A Gamma distribution was used to calculate evolutionary rate differences among sites (5 categories (+*G*, parameter = 0.6756)). Twelve amino acid sequences were used for this analysis. There were 936 positions after the calculations**. B)** AlaRS pairwise sequence calculations. The sequence identity values of the sequences in the AlaRS family is shown. The heatmap shows the identity scores as a color-coded matrix for every aaRS sequence versus every aaRS sequence in this family. Conservation increases from blue to red in the heat map.

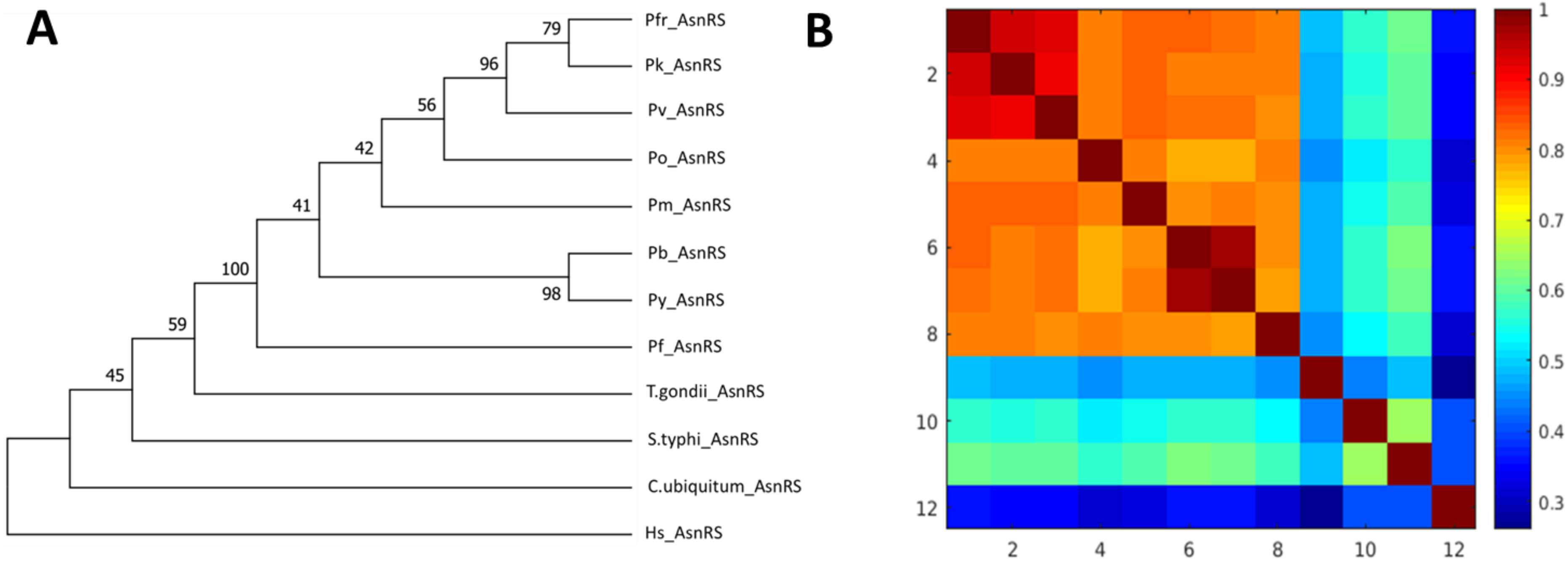

Additional file 5.13: A) AsnRS family phylogenetic tree. Maximum Likelihood method was used to infer evolutionary history using Le_Gascuel_2008 model at 100% site coverage. Phylogenetic tree calculations were done using MEGA7. The tree that had the highest log likelihood (-5013.10) is shown. Initial tree(s) for the heuristic search were obtained by using BioNJ and Neighbor-Join algorithms to a matrix of pairwise distances calculated using a JTT model, and then selecting the topology with higher log likelihood value. A Gamma distribution was used to calculate evolutionary rate differences among sites (5 categories (+*G*, parameter = 0.7387)). Twelve amino acid sequences were used for this analysis. There were 419 positions after the calculations**. B)** AsnRS pairwise sequence calculations. The sequence identity values of the sequences in the AsnRS family is shown. The heatmap shows the identity scores as a color-coded matrix for every aaRS sequence versus every aaRS sequence in this family. Conservation increases from blue to red in the heat map.

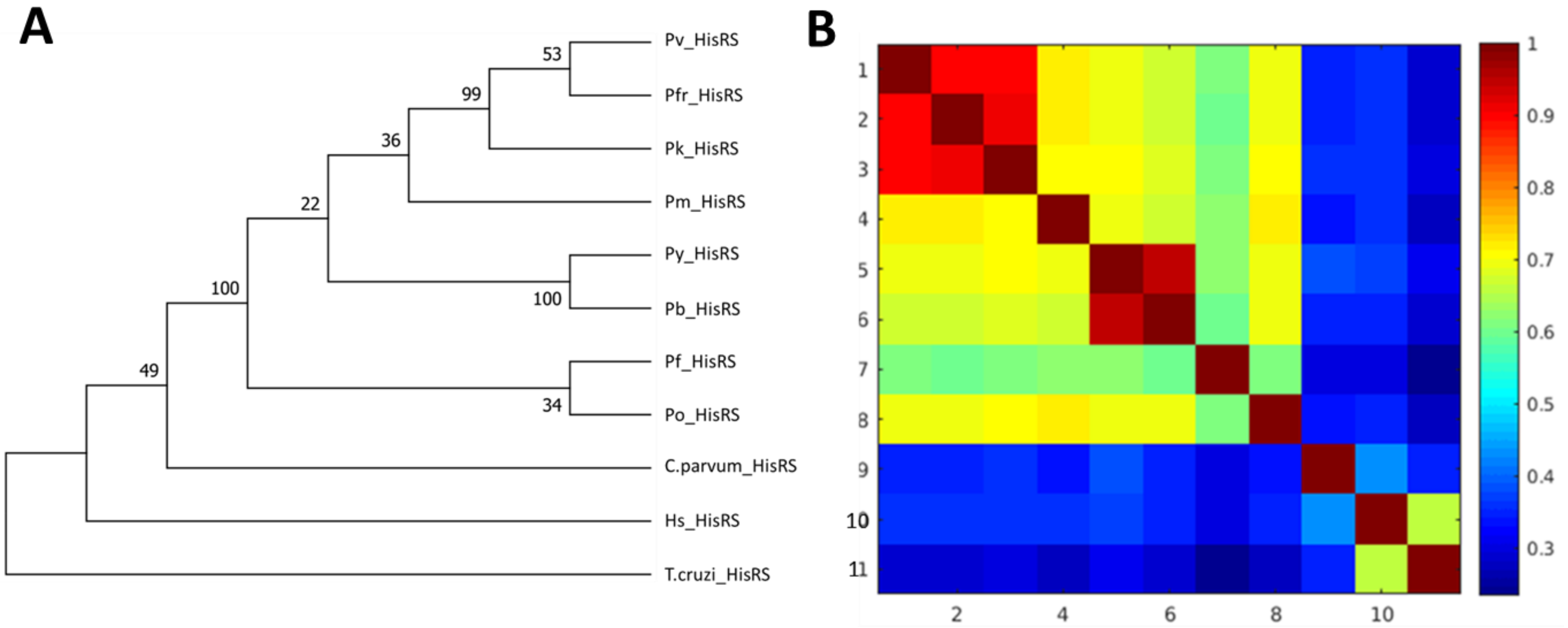

Additional file 5.14: A) HisRS family phylogenetic tree. Maximum Likelihood method was used to infer evolutionary history using JTT matrix-based model at 90% site coverage. Phylogenetic tree calculations were done using MEGA7. The tree that had the highest log likelihood (-6592.40) is shown. Initial tree(s) for the heuristic search were obtained by using BioNJ and Neighbor-Join algorithms to a matrix of pairwise distances calculated using a JTT model, and then selecting the topology with higher log likelihood value. A Gamma distribution was used to calculate evolutionary rate differences among sites (5 categories (+*G*, parameter = 0.9174)). Eleven amino acid sequences were used for this analysis. There were 530 positions after the calculations. **B)** HisRS pairwise sequence calculations. The sequence identity values of the sequences in the HisRS family is shown. The heatmap shows the identity scores as a color-coded matrix for every aaRS sequence versus every aaRS sequence in this family. Conservation increases from blue to red in the heat map.

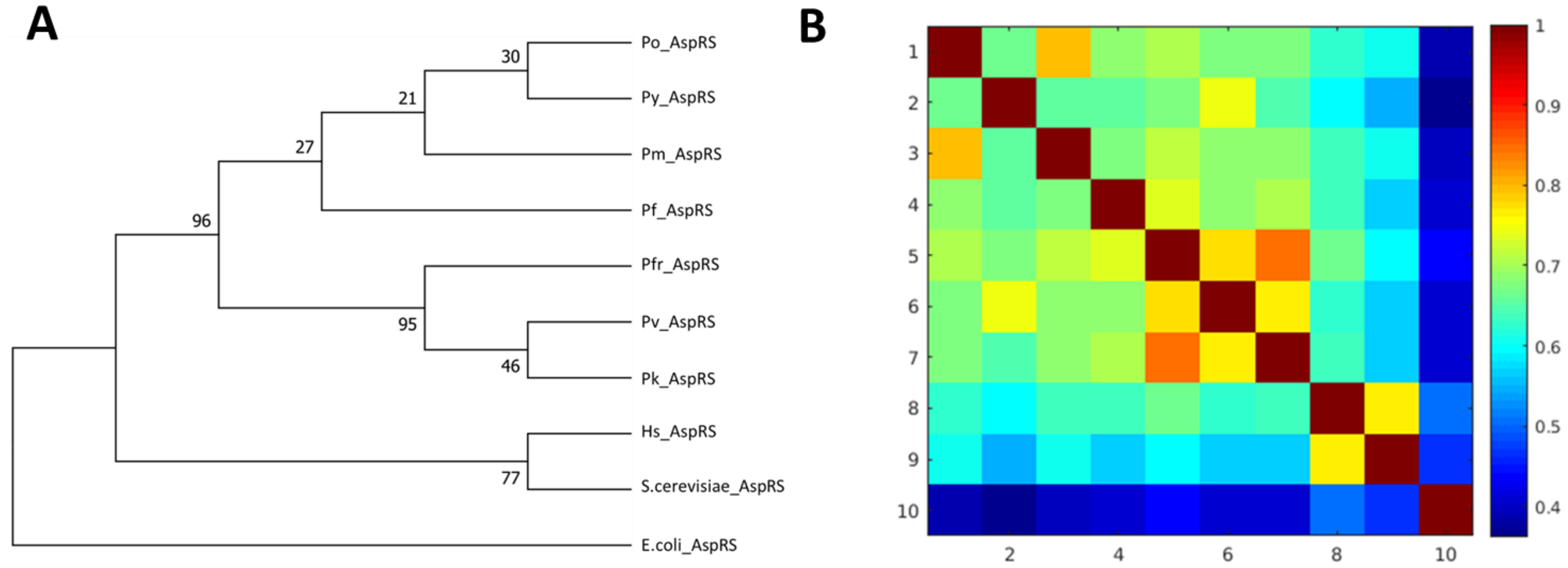

Additional file 5.15: A) AspRS family phylogenetic tree. Maximum Likelihood method was used to infer evolutionary history using Whelan And Goldman model at 90% site coverage. Phylogenetic tree calculations were done using MEGA7. The tree that had the highest log likelihood (-5487.09) is shown. Initial tree(s) for the heuristic search were obtained by using BioNJ and Neighbor-Join algorithms to a matrix of pairwise distances calculated using a JTT model, and then selecting the topology with higher log likelihood value. A Gamma distribution was used to calculate evolutionary rate differences among sites (5 categories (+*G*, parameter = 0.9989)). Ten amino acid sequences were used for this analysis. There were 443 positions after the calculations**. B)** AspRS pairwise sequence calculations. The sequence identity values of the sequences in the AspRS family is shown. The heatmap shows the identity scores as a color-coded matrix for every aaRS sequence versus every aaRS sequence in this family. Conservation increases from blue to red in the heat map.

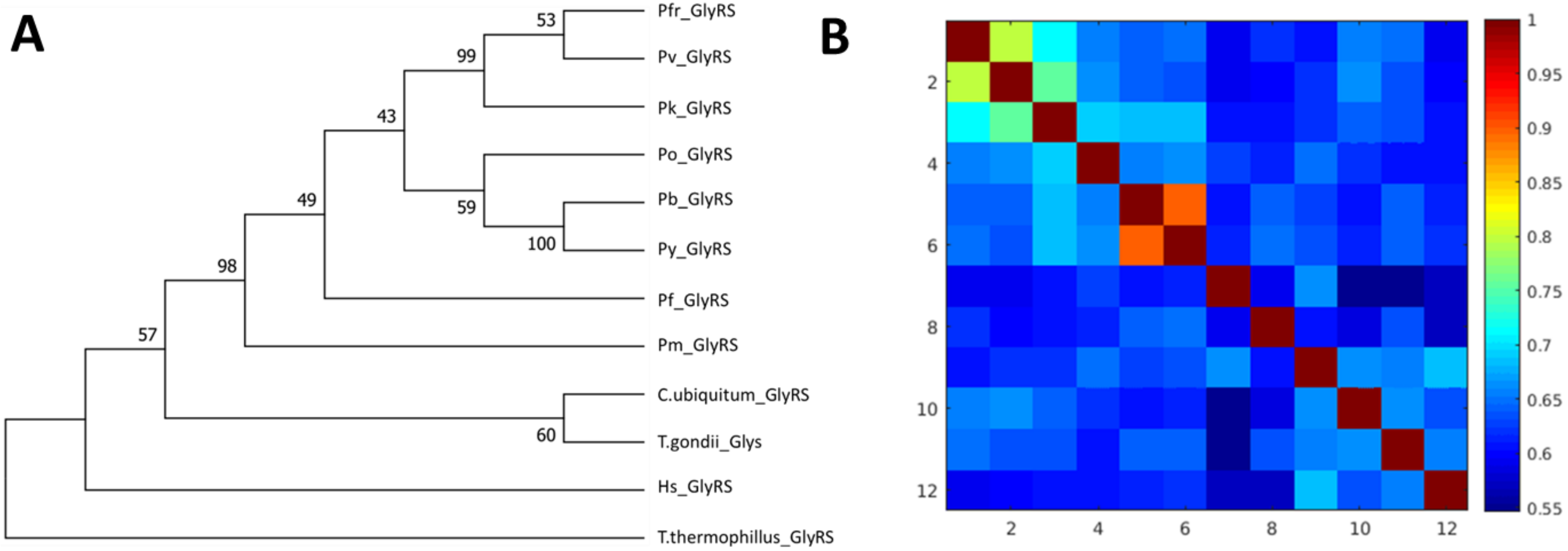

Additional file 5.16: A) GlyRS family phylogenetic tree. Maximum Likelihood method was used to infer evolutionary history using Le_Gascuel_2008 model at 90% site coverage. Phylogenetic tree calculations were done using MEGA7. The tree that had the highest log likelihood (-5569.88) is shown. Initial tree(s) for the heuristic search were obtained by using BioNJ and Neighbor-Join algorithms to a matrix of pairwise distances calculated using a JTT model, and then selecting the topology with higher log likelihood value. A Gamma distribution was used to calculate evolutionary rate differences among sites (5 categories (+*G*, parameter = 0.6003)). Twelve amino acid sequences were used for this analysis. There were 428 positions after the calculations. **B)** GlyRS pairwise sequence calculations. The sequence identity values of the sequences in the GlyRS family is shown. The heatmap shows the identity scores as a color-coded matrix for every aaRS sequence versus every aaRS sequence in this family. Conservation increases from blue to red in the heat map.

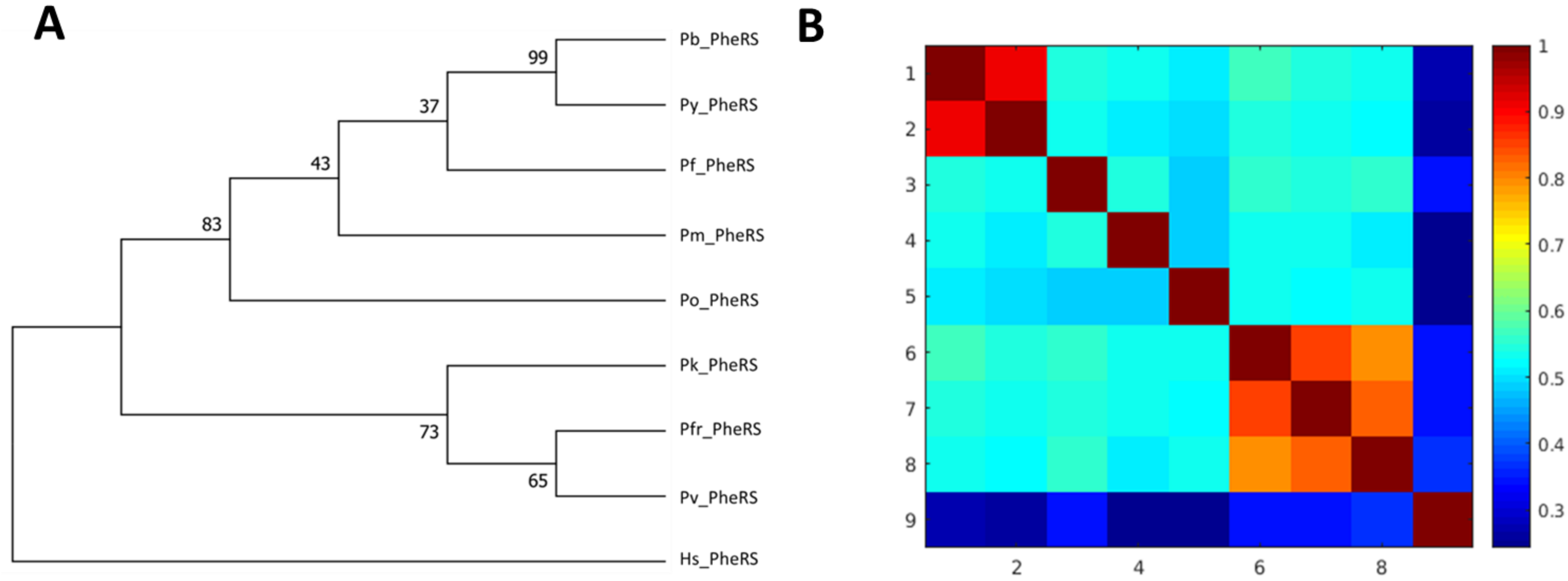

Additional file 5.17: A) PheRS family phylogenetic tree. Maximum Likelihood method was used to infer evolutionary history using Le_Gascuel_2008 model at 90% site coverage. Phylogenetic tree calculations were done using MEGA7. The tree that had the highest log likelihood (-4374.03) is shown. Initial tree(s) for the heuristic search were obtained by using BioNJ and Neighbor-Join algorithms to a matrix of pairwise distances calculated using a JTT model, and then selecting the topology with higher log likelihood value. A Gamma distribution was used to calculate evolutionary rate differences among sites (5 categories (+*G*, parameter = 0.9196)). Nine amino acid sequences were used for this analysis. There were 360 positions after the calculations. **B)** PheRS pairwise sequence calculations. The sequence identity values of the sequences in the PheRS family is shown. The heatmap shows the identity scores as a color-coded matrix for every aaRS sequence versus every aaRS sequence in this family. Conservation increases from blue to red in the heat map.

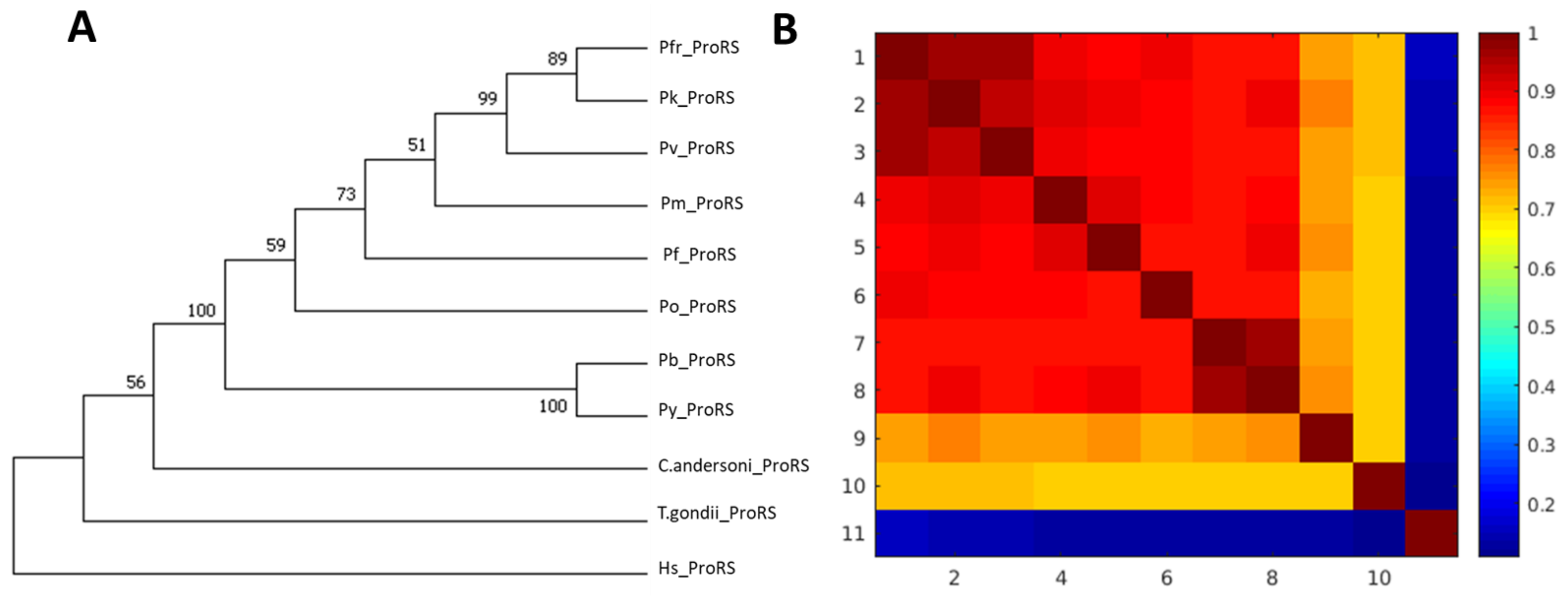

Additional file 5.18: A) ProRS family phylogenetic tree. Maximum Likelihood method was used to infer evolutionary history using Le_Gascuel_2008 model at 100% site coverage. Phylogenetic tree calculations were done using MEGA7. The tree that had the highest log likelihood (-3767.96) is shown. Initial tree(s) for the heuristic search were obtained by using BioNJ and Neighbor-Join algorithms to a matrix of pairwise distances calculated using a JTT model, and then selecting the topology with higher log likelihood value. A Gamma distribution was used to calculate evolutionary rate differences among sites (5 categories (+*G*, parameter = 0.9935)). The rate variation model allowed for some sites to be evolutionarily invariable ([+*I*], 13.65% sites). Eleven amino acid sequences were used for this analysis. There were 315 positions after the calculations. **B)** ProRS pairwise sequence calculations. The sequence identity values of the sequences in the ProRS family is shown. The heatmap shows the identity scores as a color-coded matrix for every aaRS sequence versus every aaRS sequence in this family. Conservation increases from blue to red in the heat map.

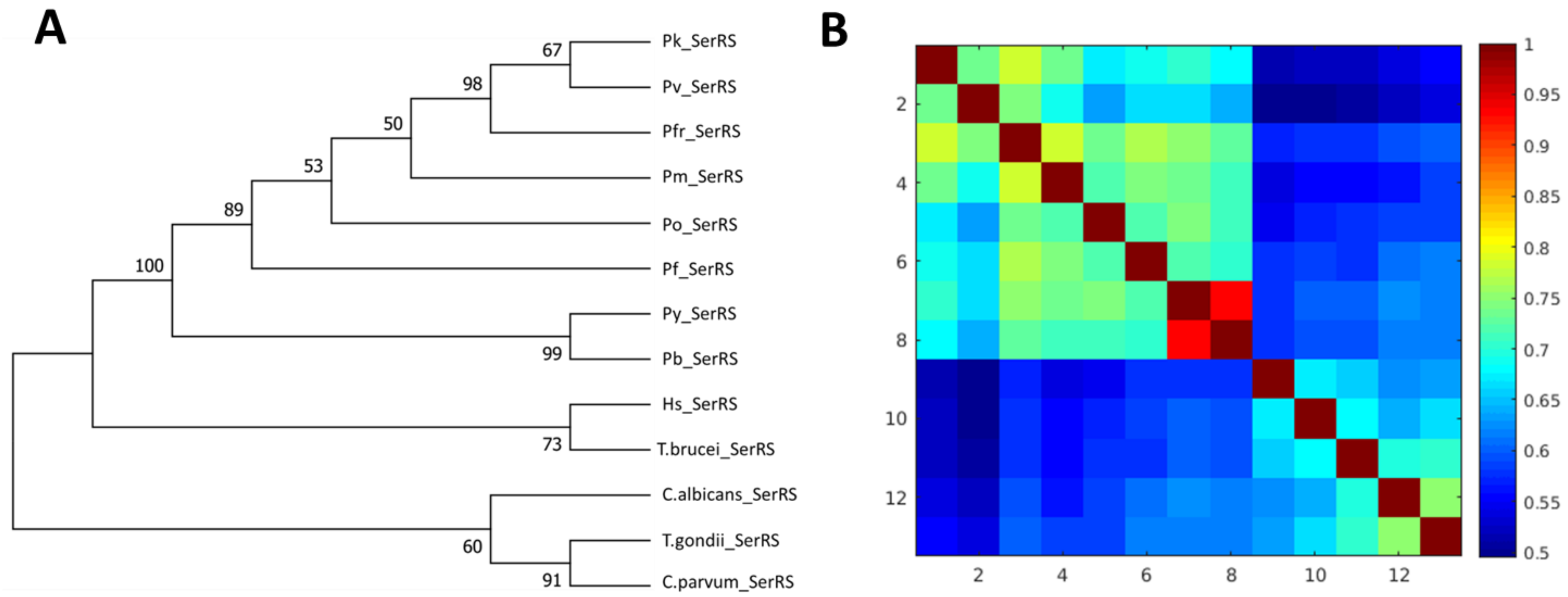

Additional file 5.19: A) SerRS family phylogenetic tree. Maximum Likelihood method was used to infer evolutionary history using Le_Gascuel_2008 model at 90% site coverage. Phylogenetic tree calculations were done using MEGA7. The tree that had the highest log likelihood (-5474.69) is shown. Initial tree(s) for the heuristic search were obtained by using BioNJ and Neighbor-Join algorithms to a matrix of pairwise distances calculated using a JTT model, and then selecting the topology with higher log likelihood value. A Gamma distribution was used to calculate evolutionary rate differences among sites (5 categories (+*G*, parameter = 0.8707)). Thirteen amino acid sequences were used for this analysis. There were 402 positions after the calculations. **B)** SerRS pairwise sequence calculations. The sequence identity values of the sequences in the SerRS family is shown. The heatmap shows the identity scores as a color-coded matrix for every aaRS sequence versus every aaRS sequence in this family. Conservation increases from blue to red in the heat map.

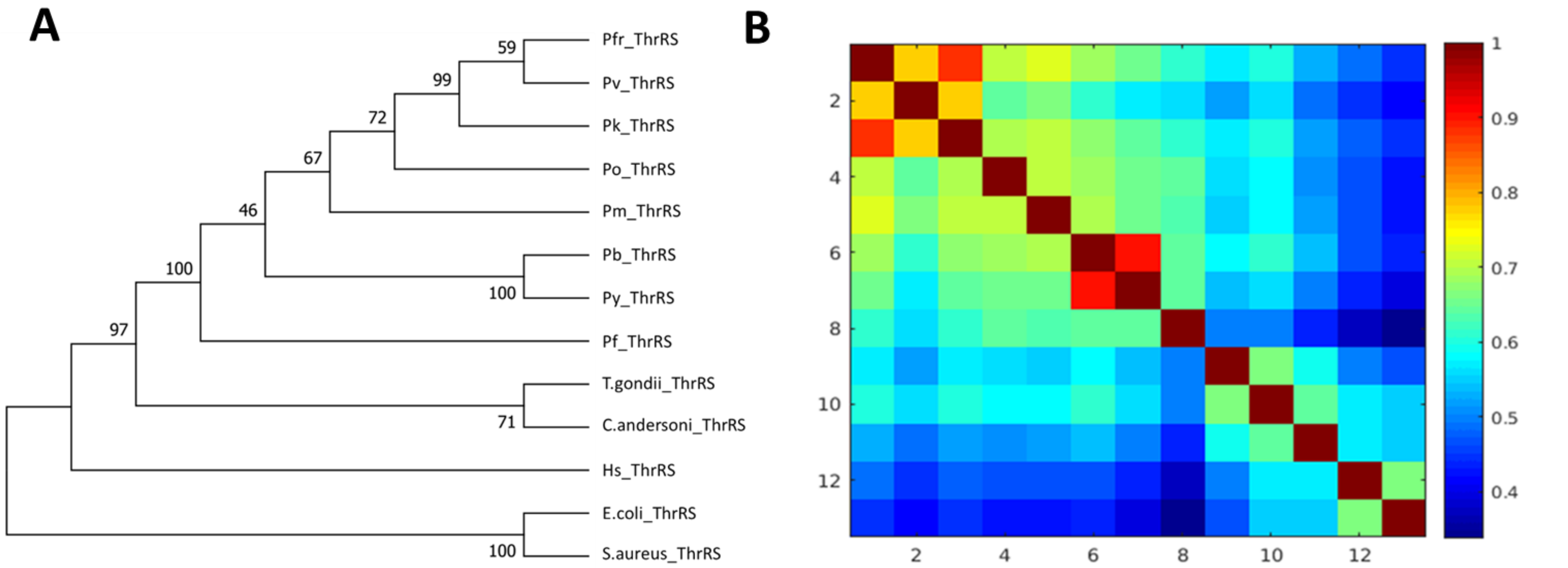

Additional file 5.20: A) ThrRS family phylogenetic tree. Maximum Likelihood method was used to infer evolutionary history using General Reverse Transcriptase model at 95% site coverage. Phylogenetic tree calculations were done using MEGA7. The tree that had the highest log likelihood (-8454.38) is shown. Initial tree(s) for the heuristic search were obtained by using BioNJ and Neighbor-Join algorithms to a matrix of pairwise distances calculated using a JTT model, and then selecting the topology with higher log likelihood value. A Gamma distribution was used to calculate evolutionary rate differences among sites (5 categories (+*G*, parameter = 1.0429)). Thirteen amino acid sequences were used for this analysis. There were 575 positions after the calculations. **B)** ThrRS pairwise sequence calculations. The sequence identity values of the sequences in the ThrRS family is shown. The heatmap shows the identity scores as a color-coded matrix for every aaRS sequence versus every aaRS sequence in this family. Conservation increases from blue to red in the heat map.
