# Supplementary material for: Aminoacyl tRNA synthetases as malarial drug targets: a comparative bioinformatics study
Source: Malar J. 2019 Feb 6;18:34. doi: 10.1186/s12936-019-2665-6 (PMC6366043; doi:10.1186/s12936-019-2665-6)
Supplement: Supplementary file 6 — Additional file 6. Mapping of unique motifs to homology models in Plasmodium ArgRS, MetRS, TrpRS, TyrRS, LysRS and ProRS families and the respective human homologues. Motif numbering for each protein is based on the MEME results. [file 12936_2019_2665_MOESM6_ESM.pdf]

Additional file 6: Mapping of discovered motif to homology models of ArgRS, MetRS, TrpRS, TyrRS, LysRS and ProRS. Motif mapping was done using a python script. Motif numbering is based on the MEME results.

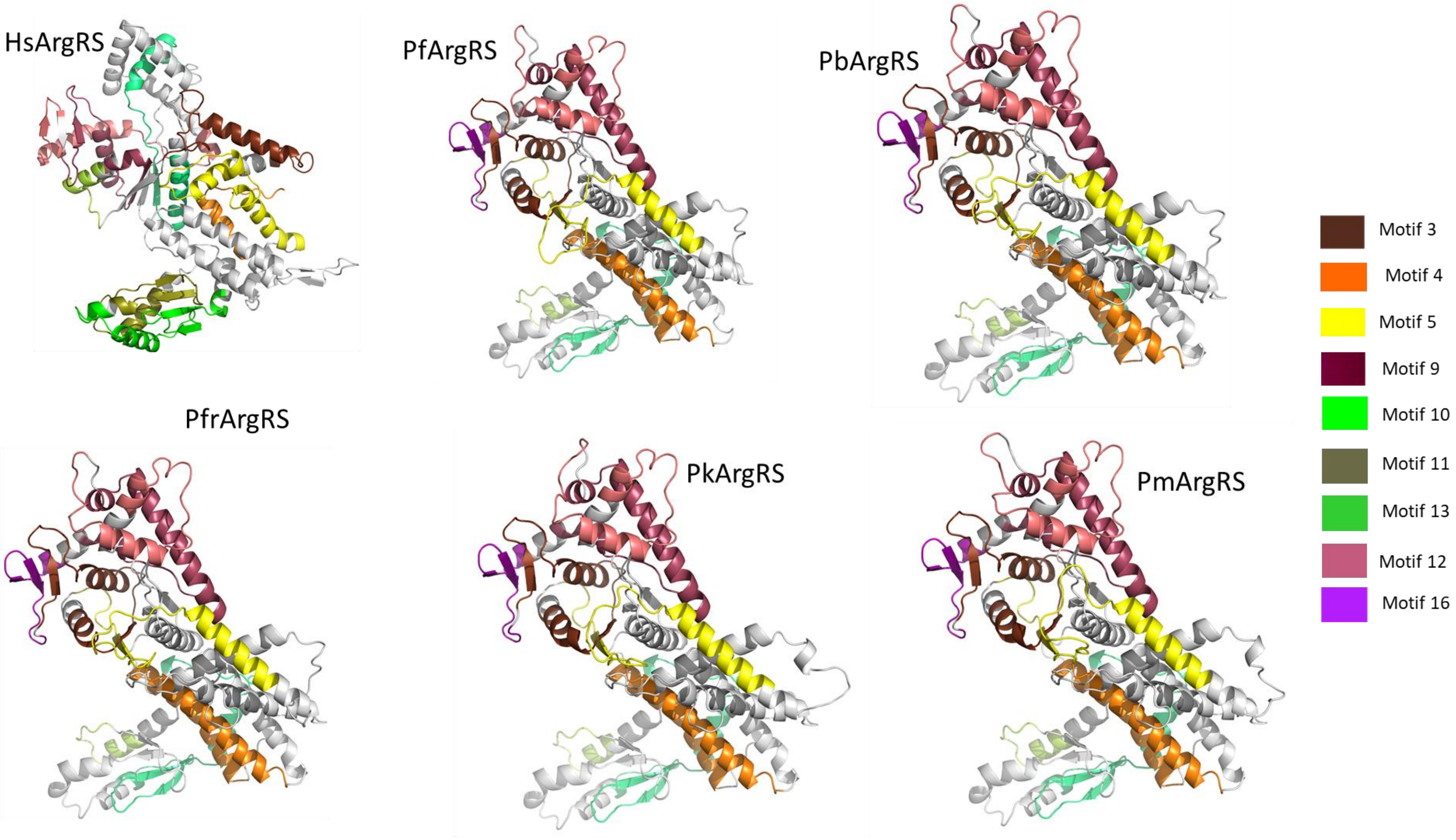

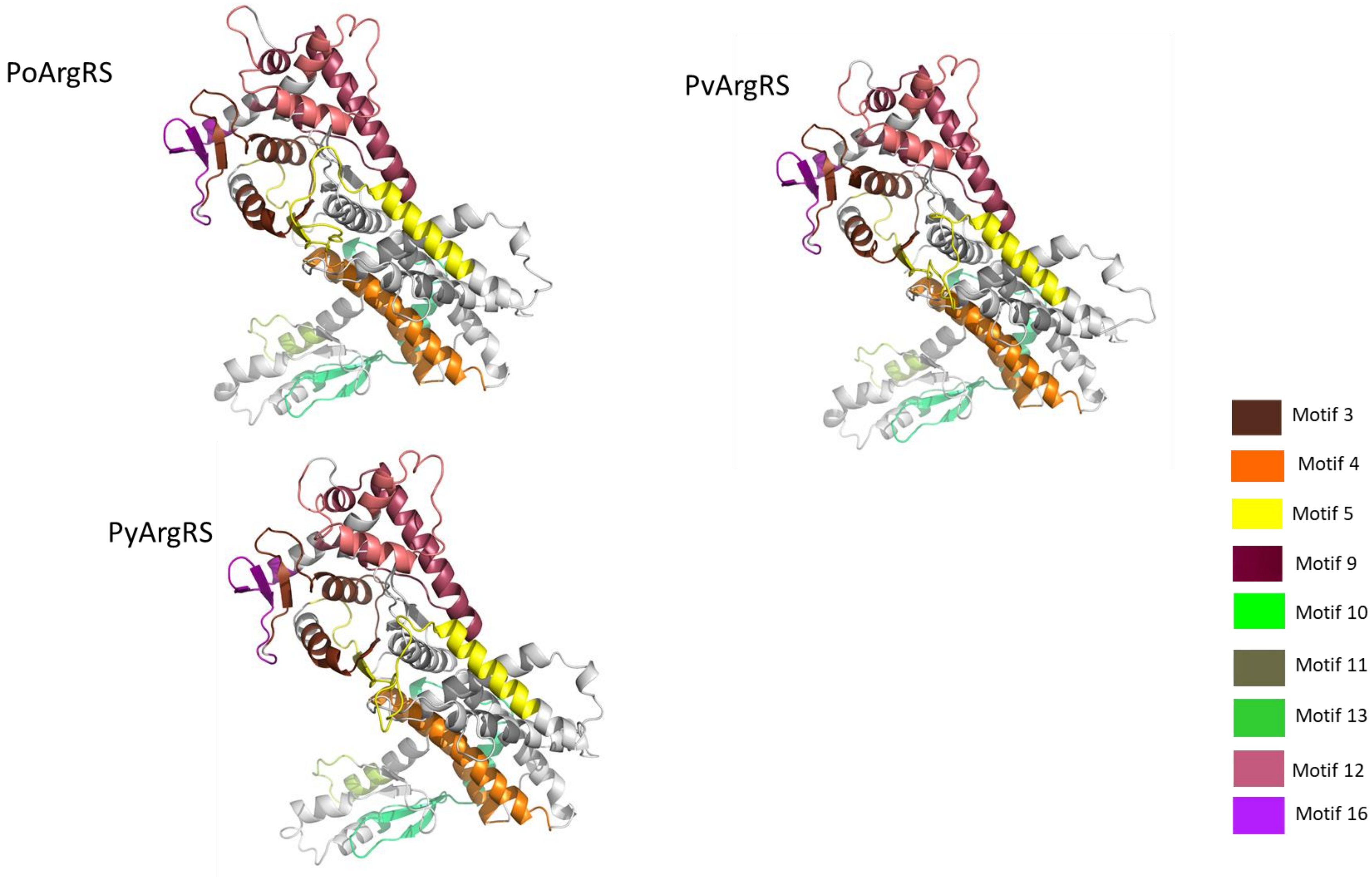

Additional file 6A: Mapping of motifs identified for ArgRS to homology models.

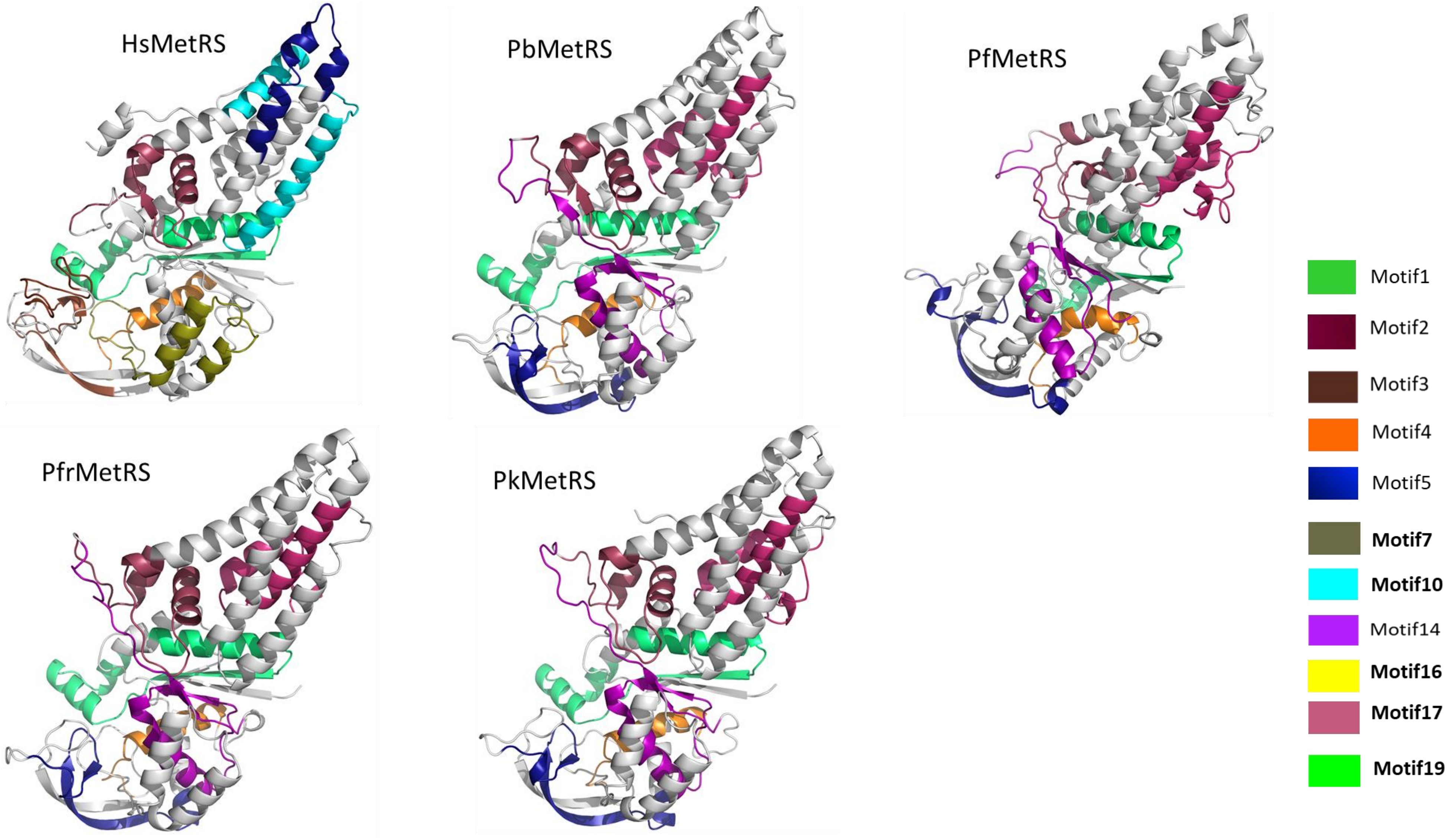
HsMetRS
PbMetRS
PfMetRS
PfrMetRS
PkMetRS
Motif1
Motif2
Motif3
Motif4
Motif5
Motif7
Motif10
Motif14
Motif16
Motif17
Motif19

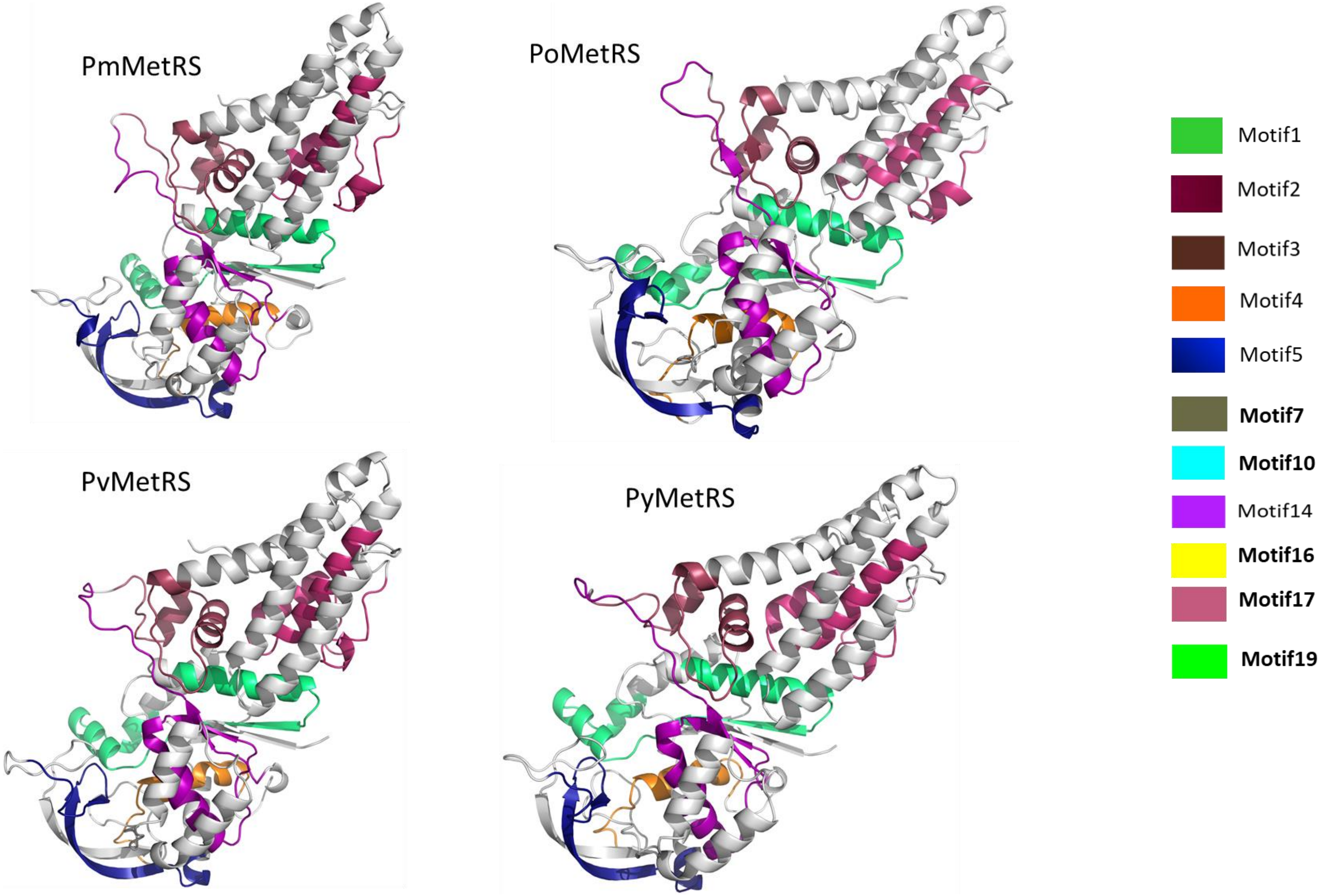

Additional file 6B: Mapping of motifs identified for MetRS to homology models.

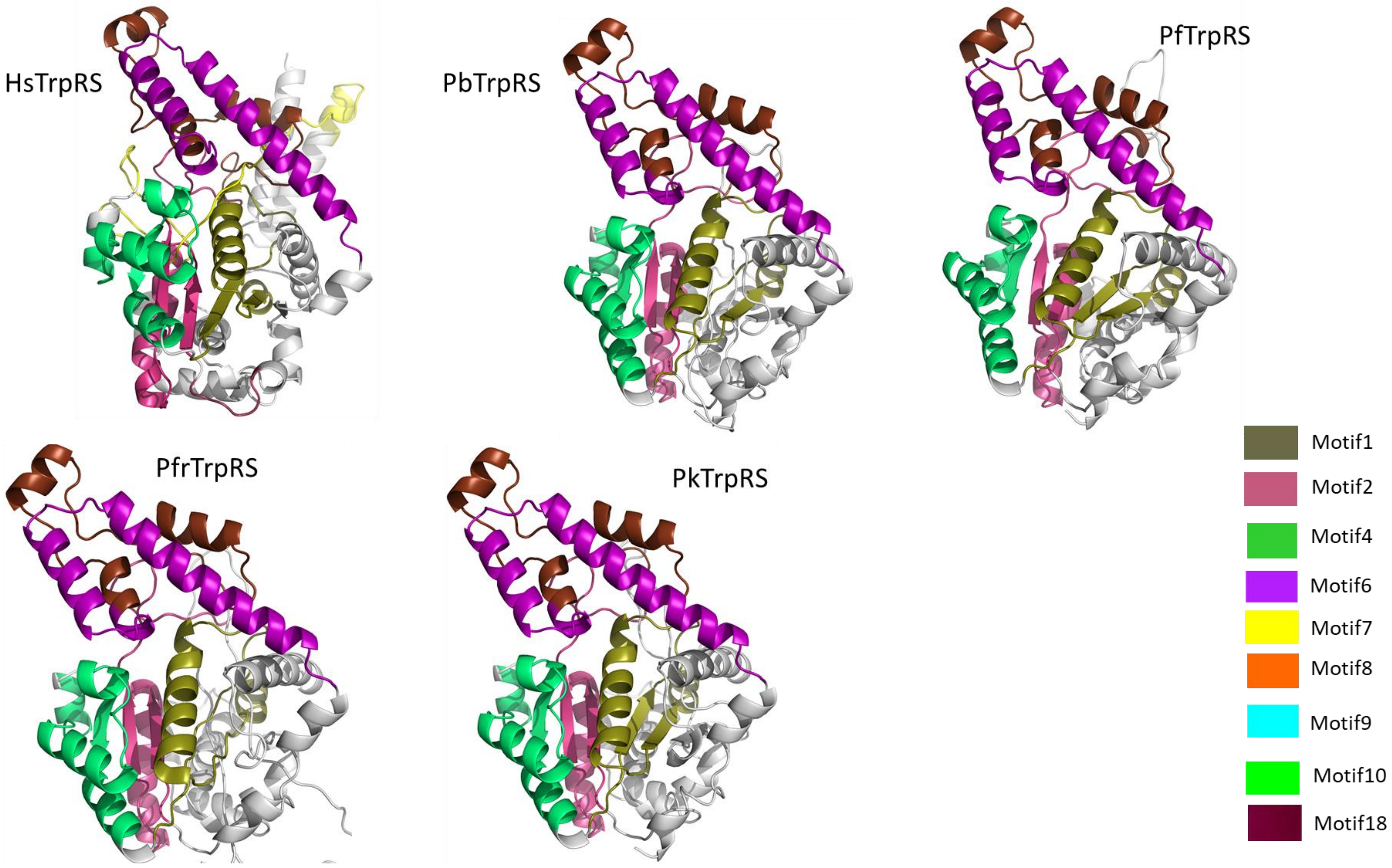
HsTrpRS
PbTrpRS
PfTrpRS
PfrTrpRS
PkTrpRS
Motif1
Motif2
Motif4
Motif6
Motif7
Motif8
Motif9
Motif10
Motif18

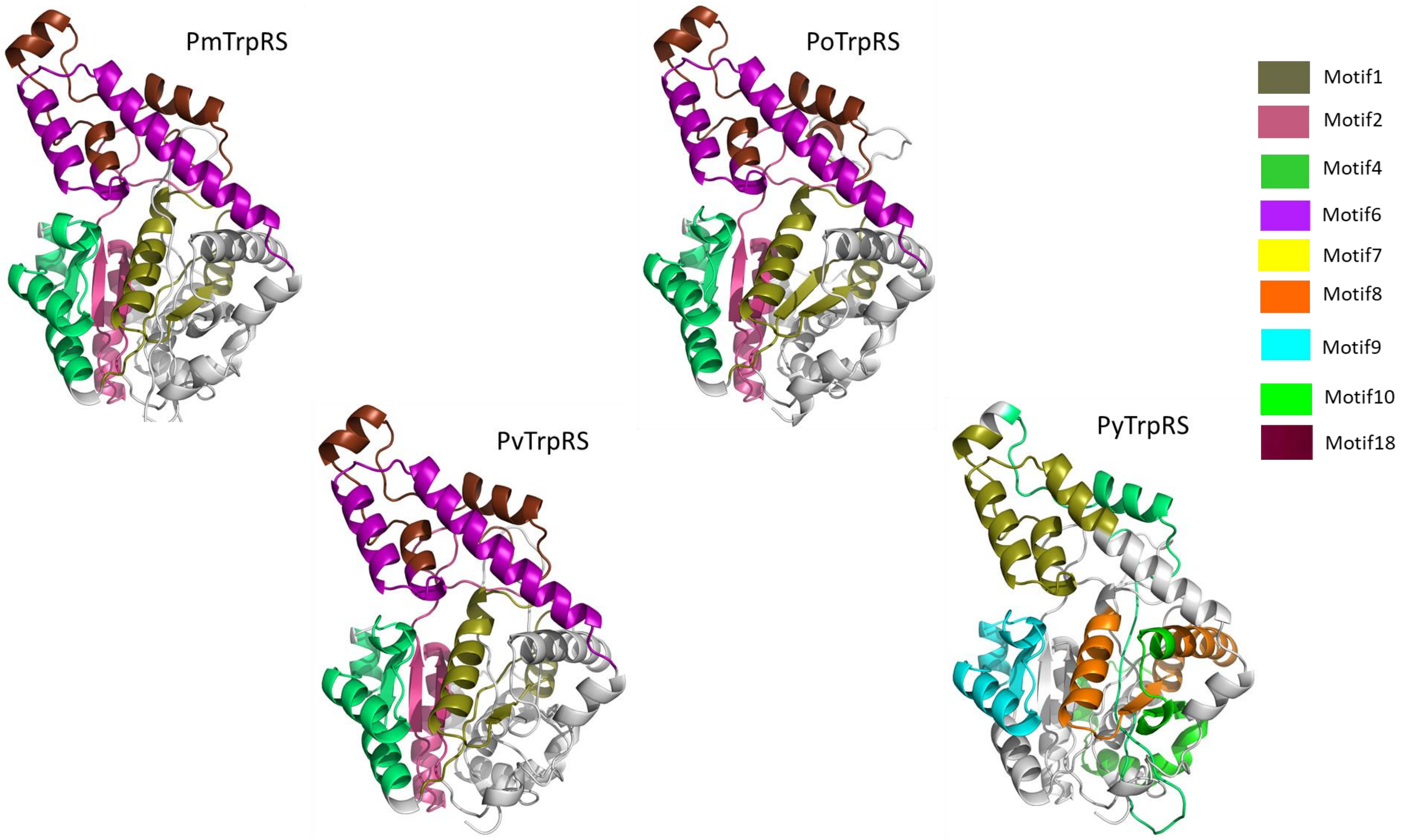

Additional file 6C: Mapping of motifs identified for TrpRS to homology models.

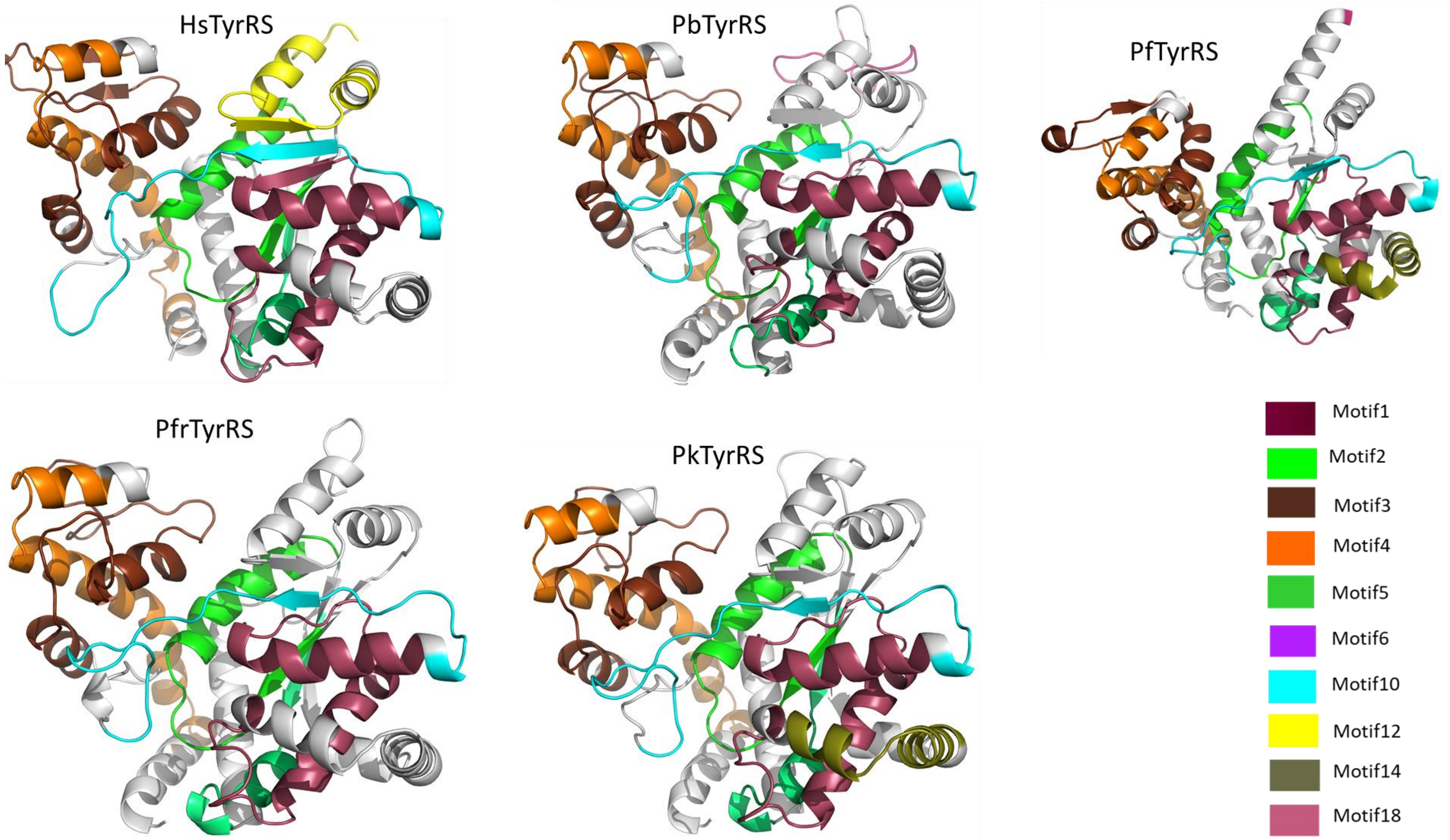
HsTyrRS
PbTyrRS
PfTyrRS
PfrTyrRS
PkTyrRS
Motif1
Motif2
Motif3
Motif4
Motif5
Motif6
Motif10
Motif12
Motif14
Motif18

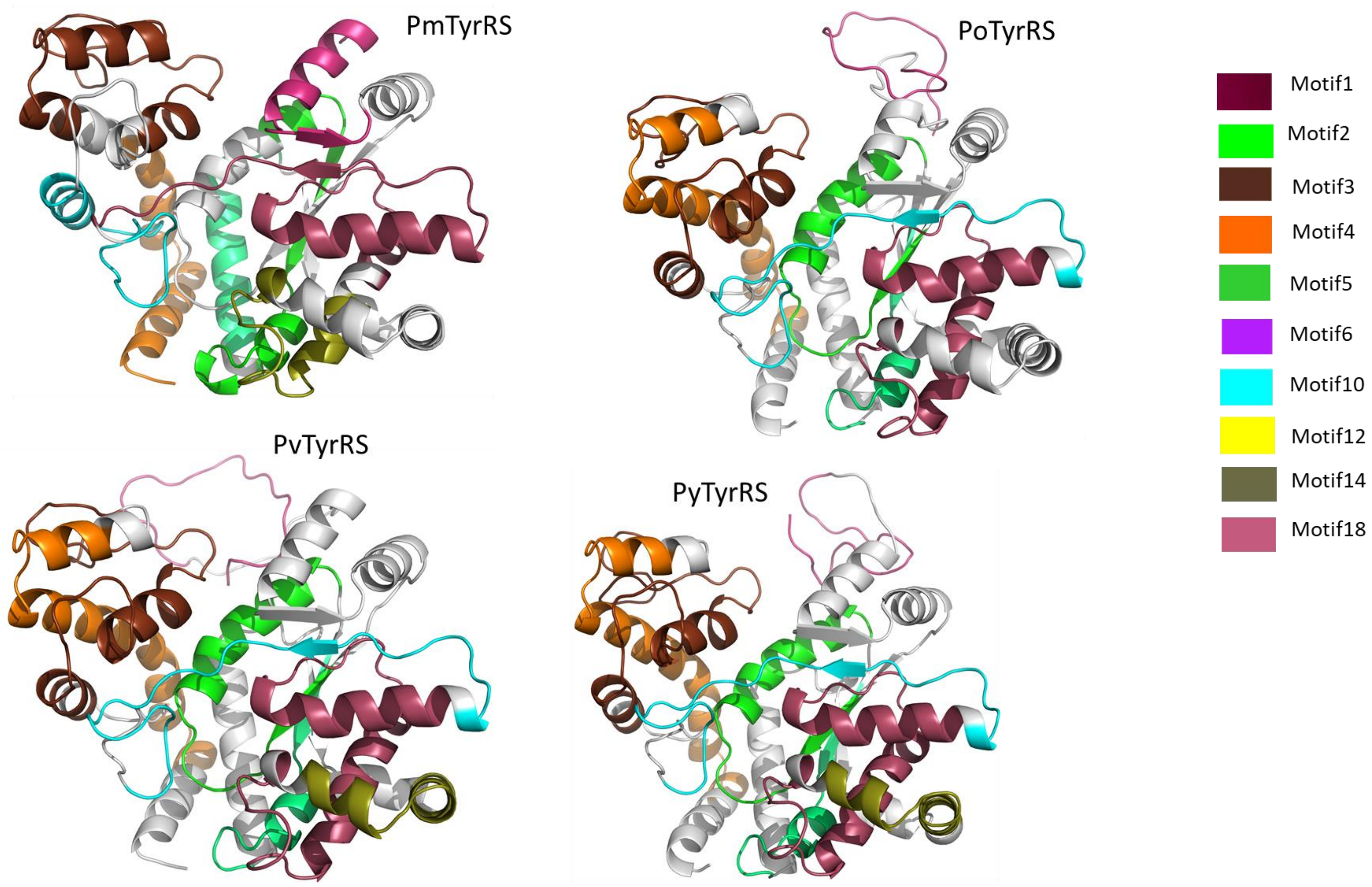

Additional file 6D: Mapping of motifs identified for TyrRS to homology models.

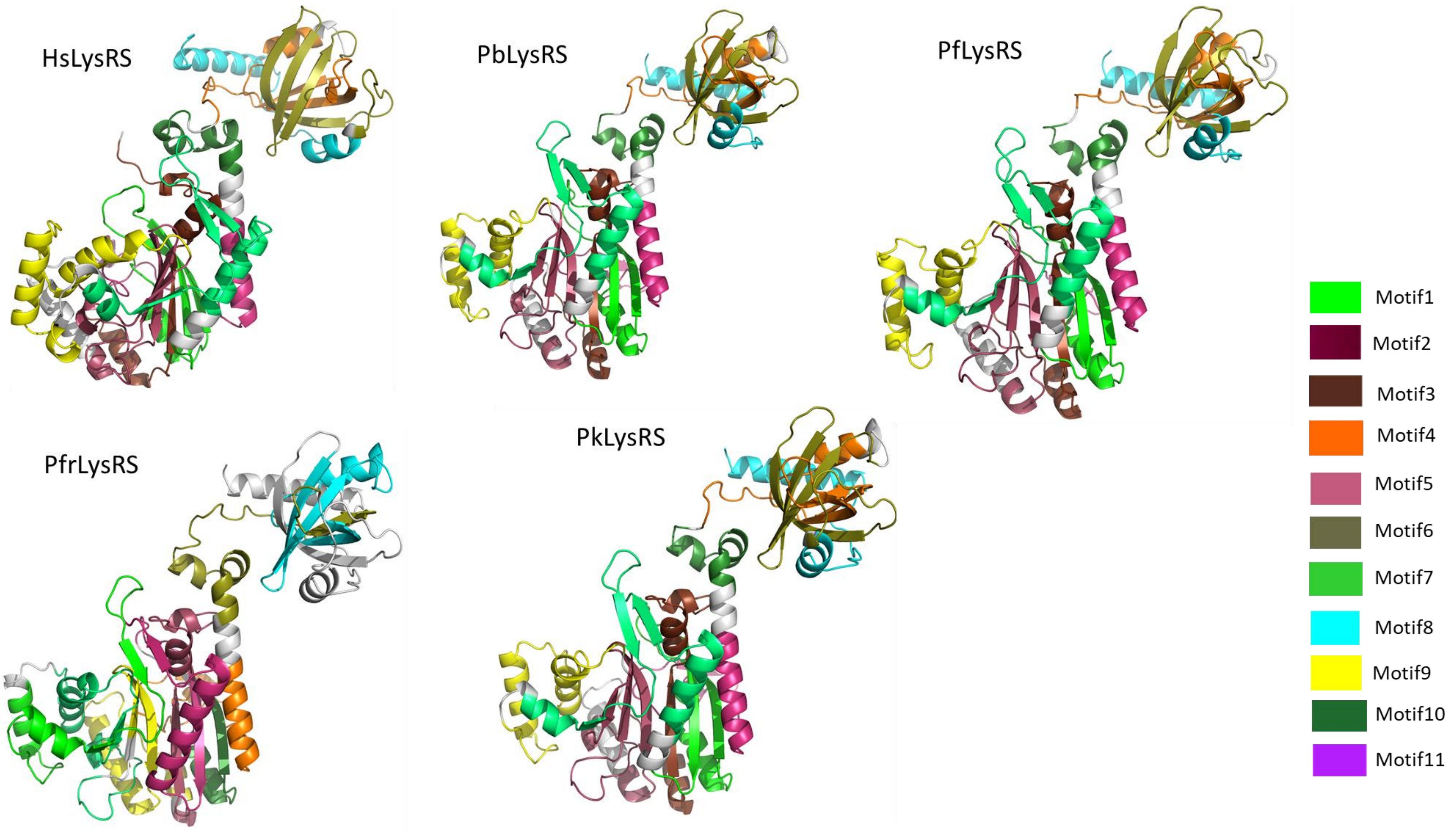
HsLysRS
PbLysRS
PfLysRS
PfrLysRS
PkLysRS
Motif1
Motif2
Motif3
Motif4
Motif5
Motif6
Motif7
Motif8
Motif9
Motif10
Motif11

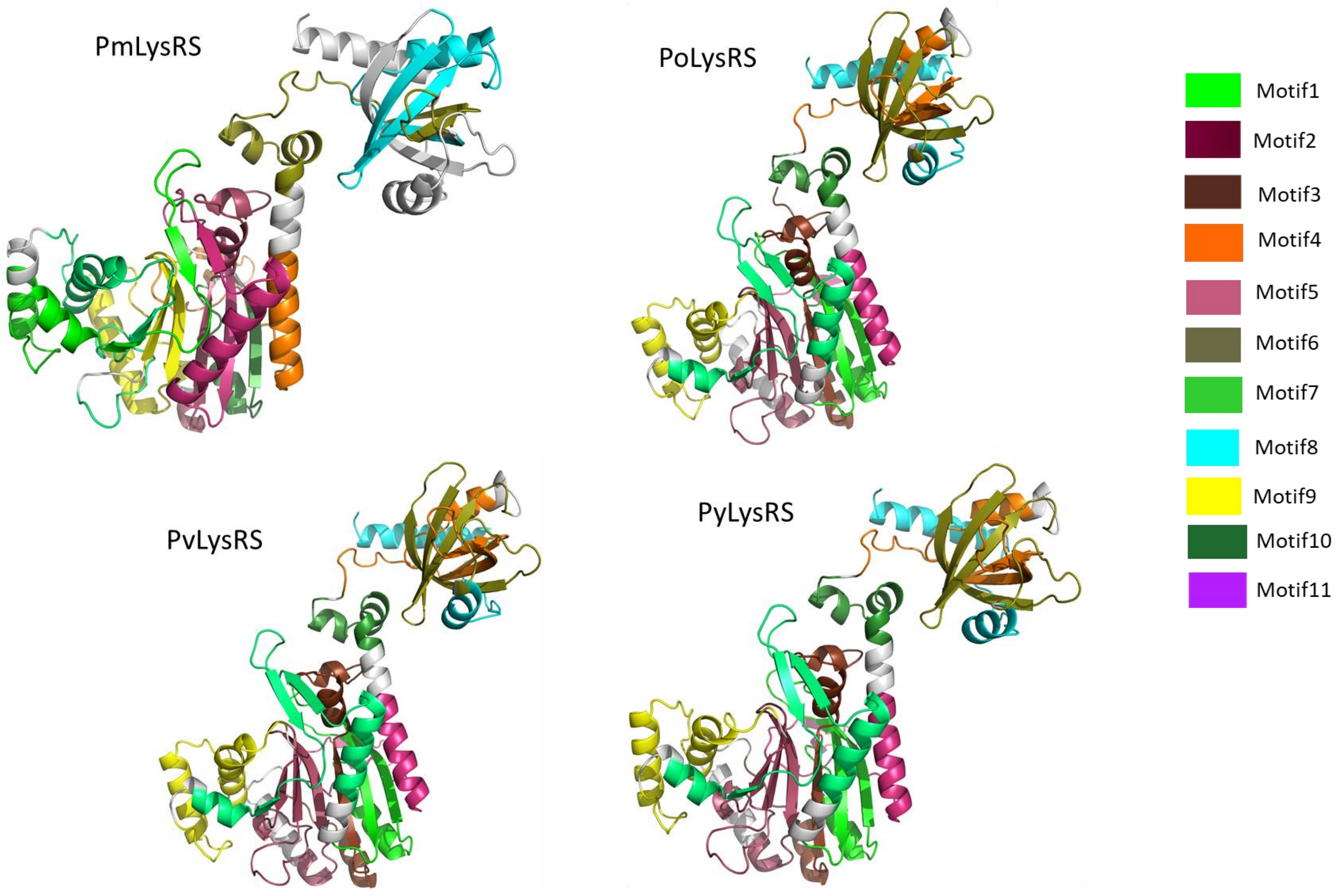

Additional file 6E: Mapping of motifs identified for LysRS to homology models.

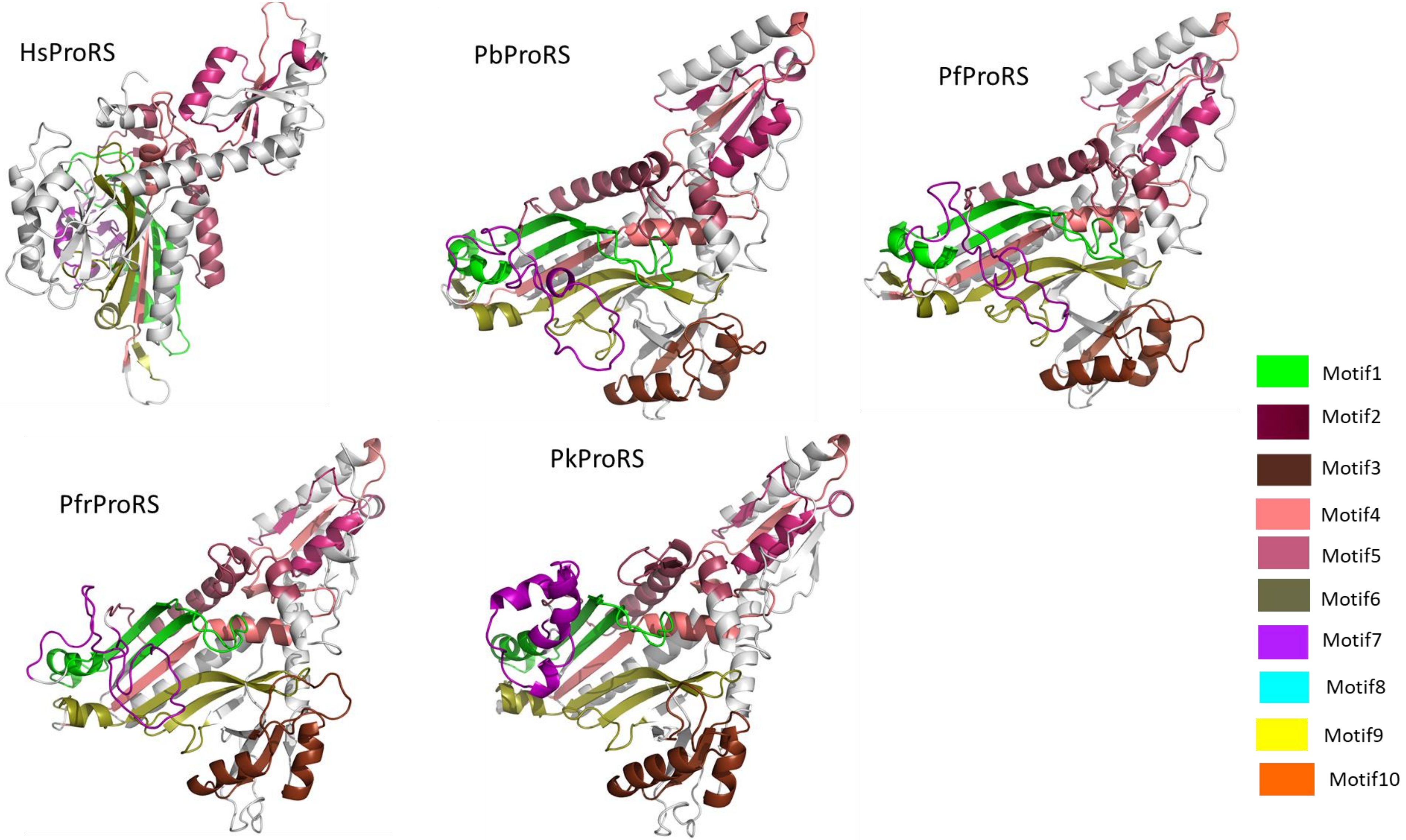
HsProRS
PbProRS
PfProRS
PfrProRS
PkProRS
Motif1
Motif2
Motif3
Motif4
Motif5
Motif6
Motif7
Motif8
Motif9
Motif10

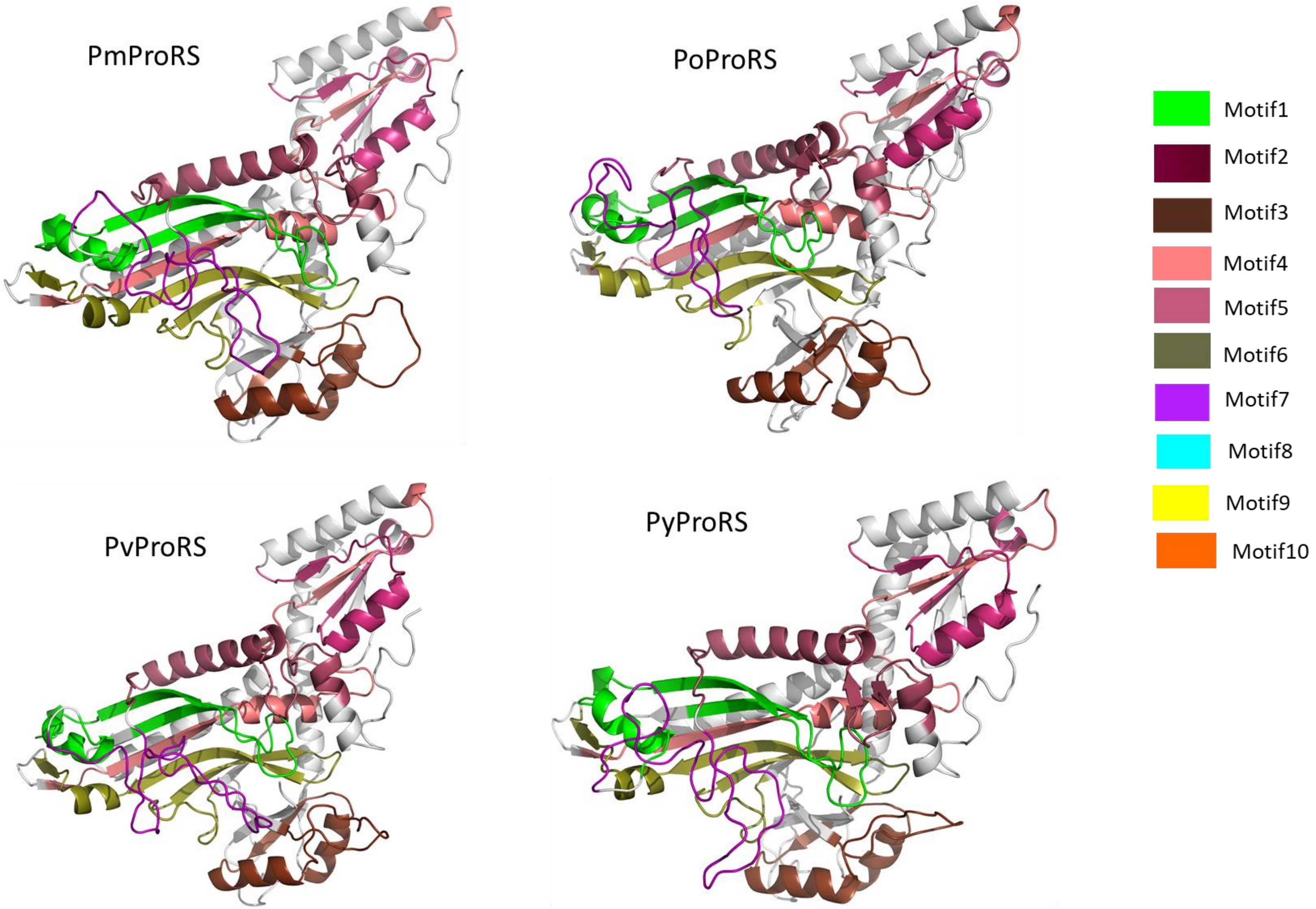

Additional file 6F: Mapping of motifs identified for ProRS to homology models.
